# Supplementary figures and images for: Autonomous Reaction Network Exploration in Homogeneous and Heterogeneous Catalysis
Source: Top Catal. 2022 Jan 13;65(1-4):6–39. doi: 10.1007/s11244-021-01543-9 (PMC8816766; doi:10.1007/s11244-021-01543-9)

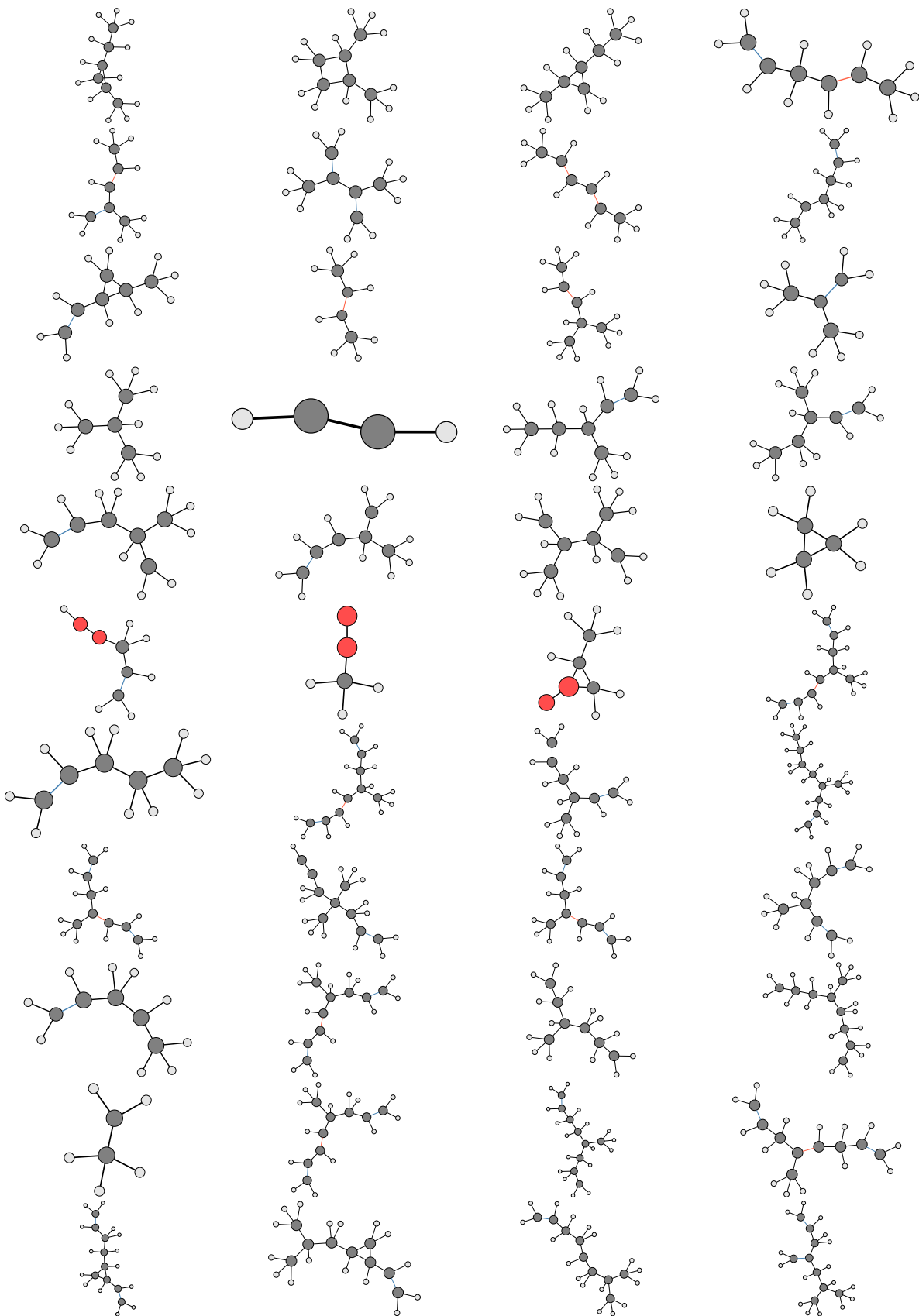

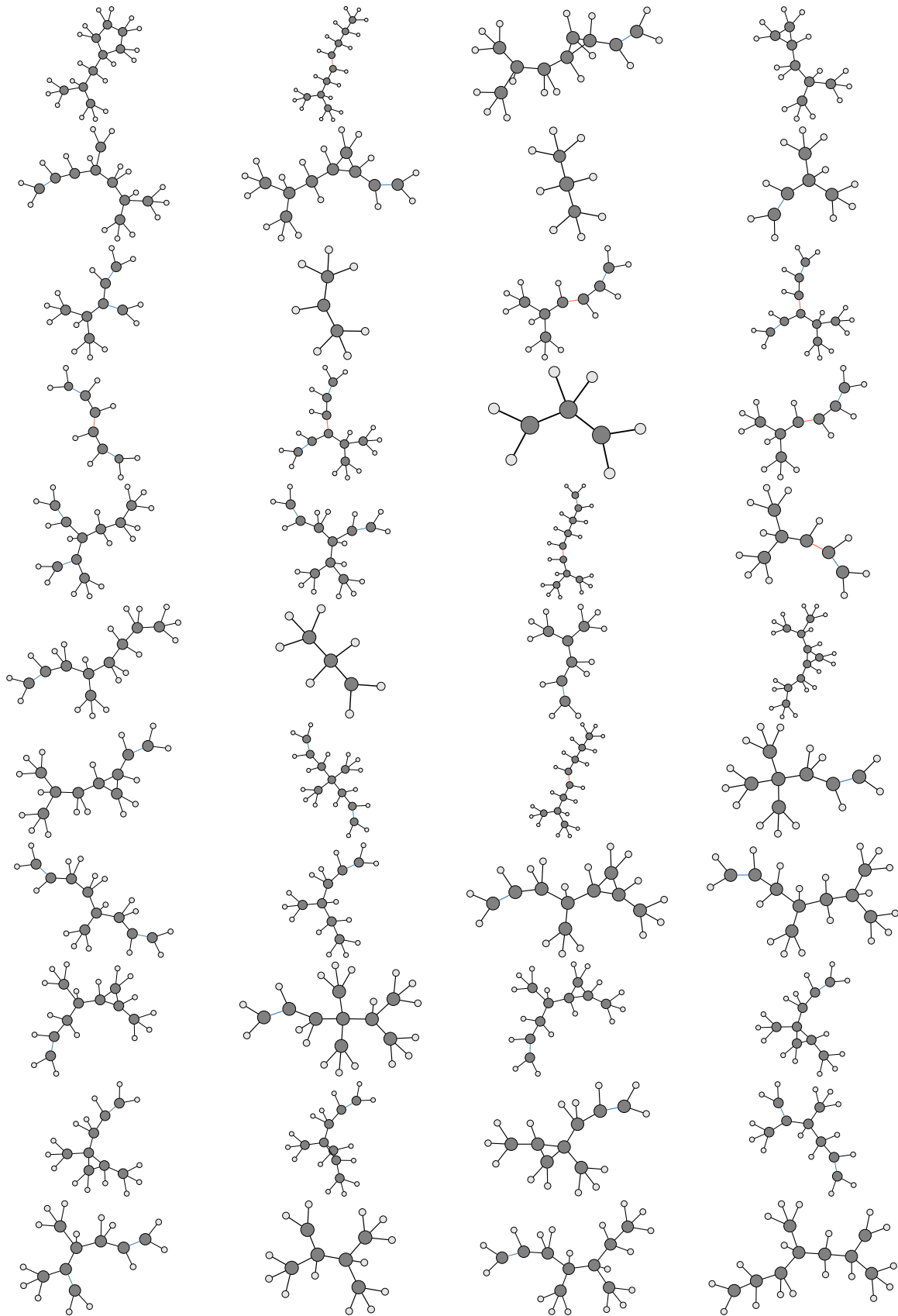

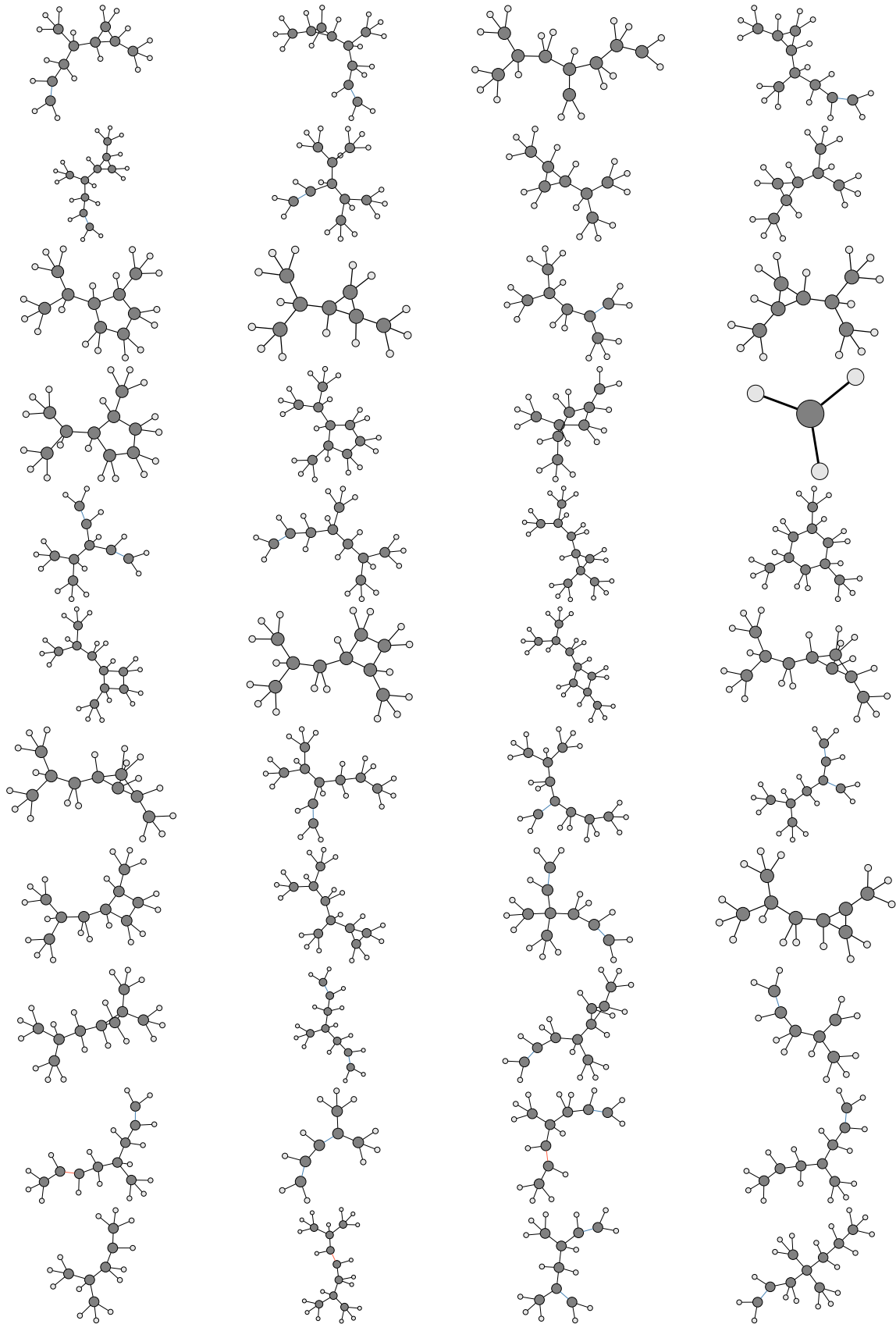

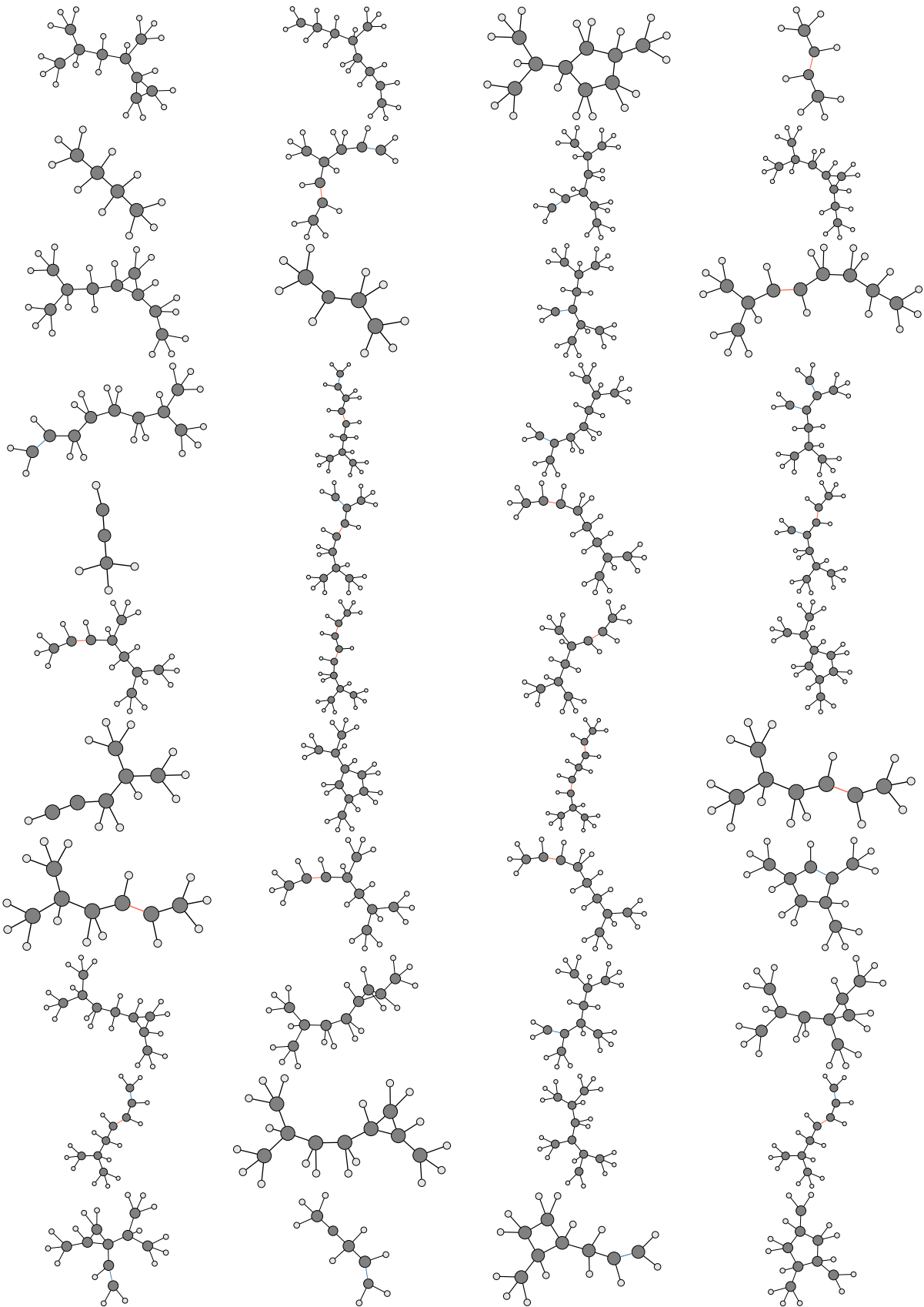



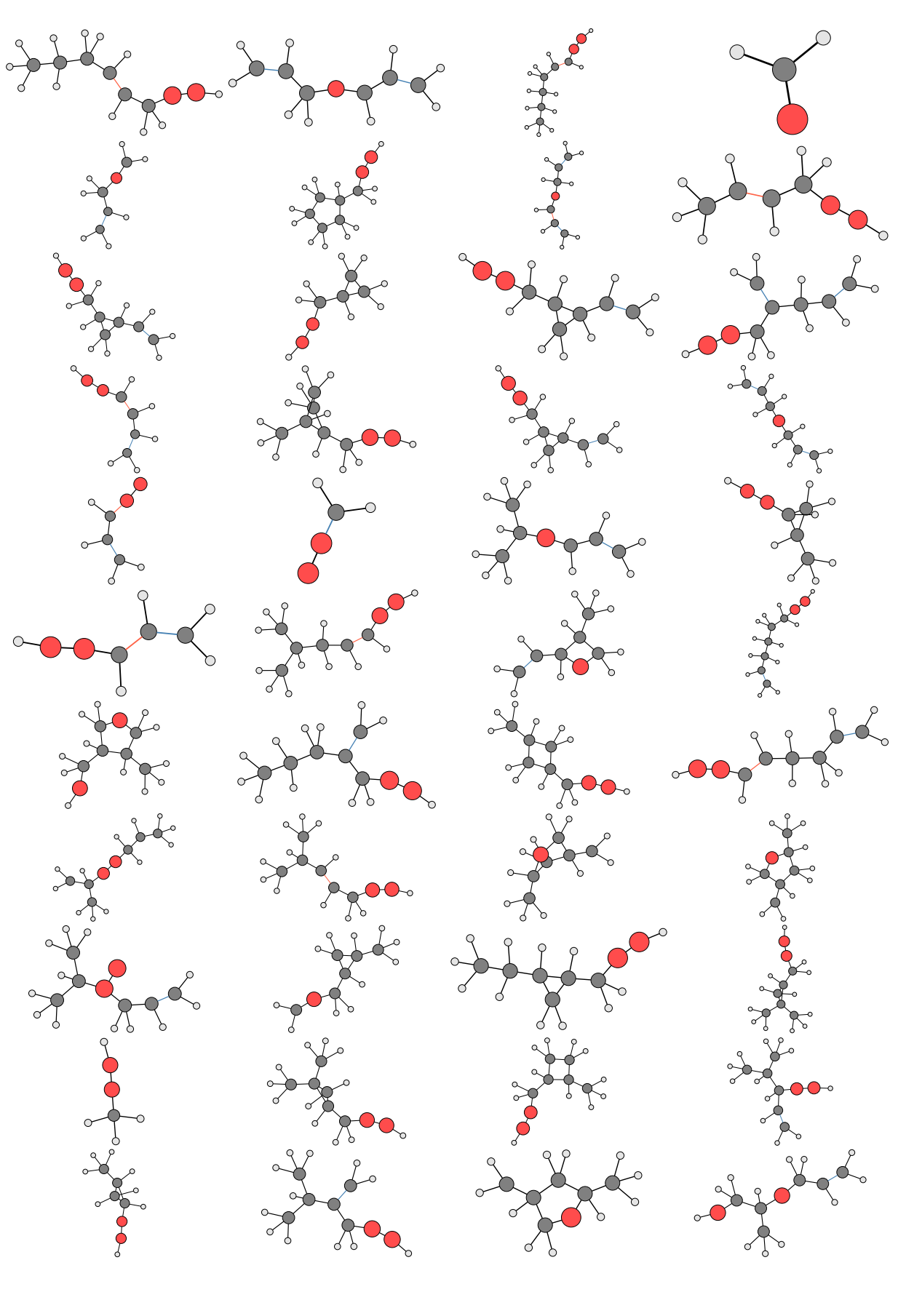

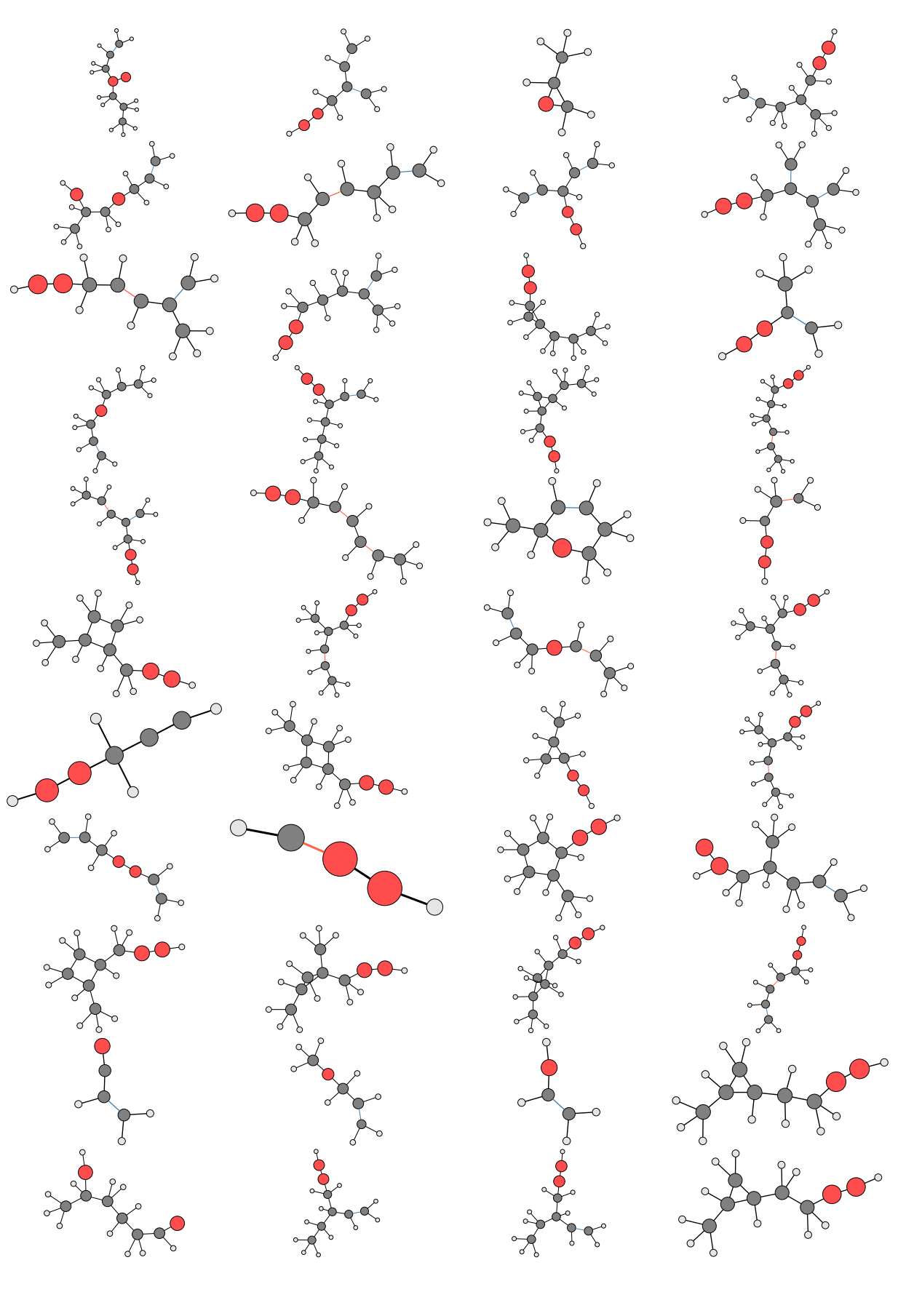

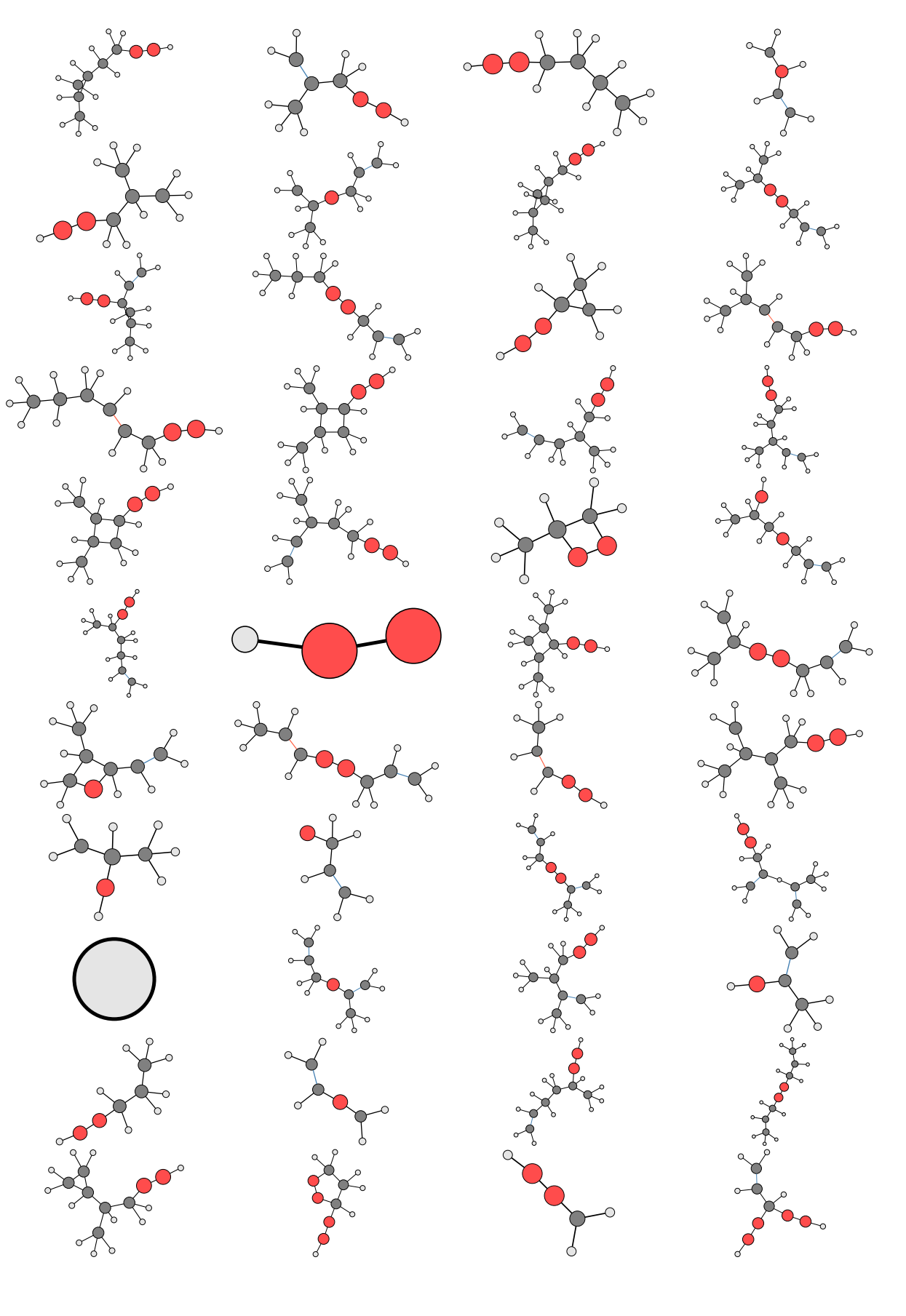

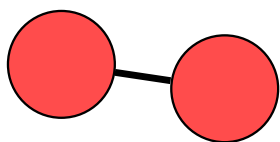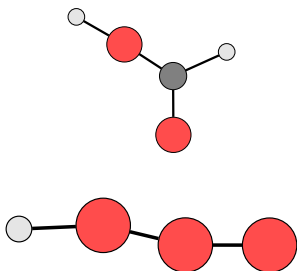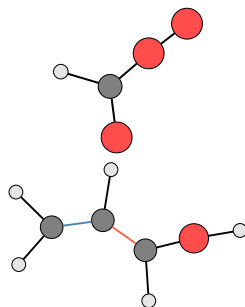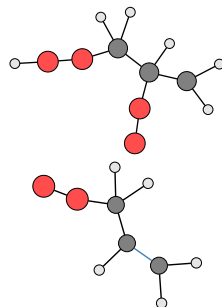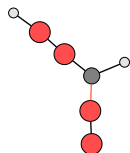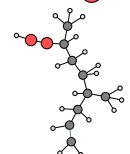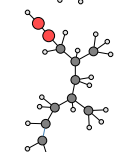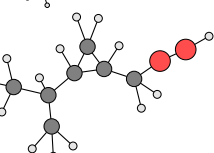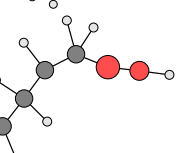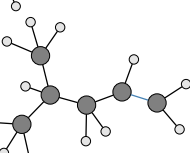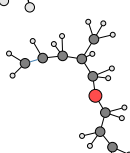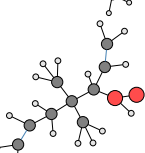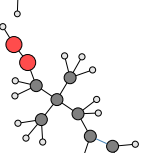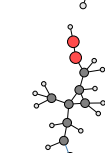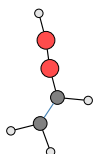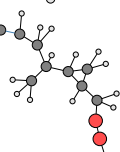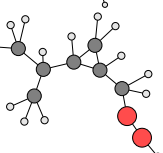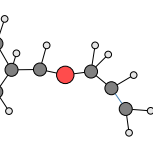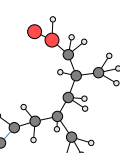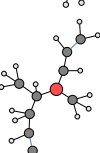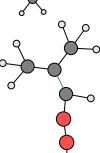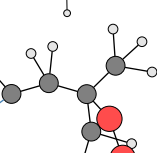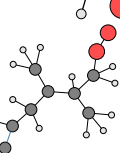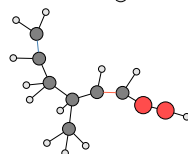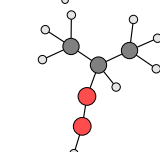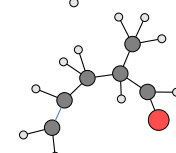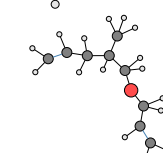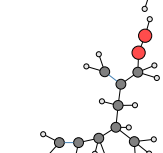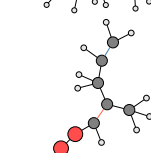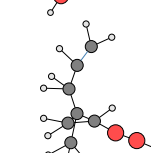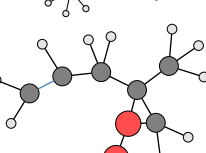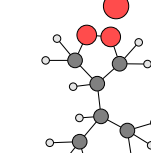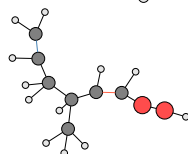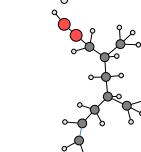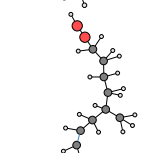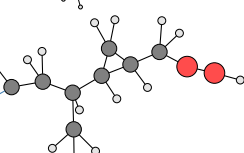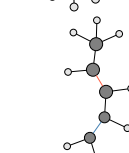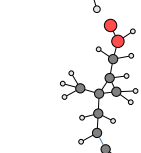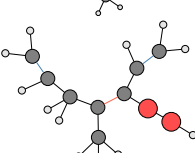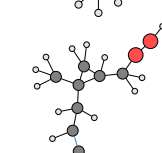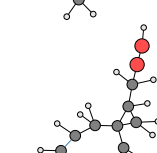

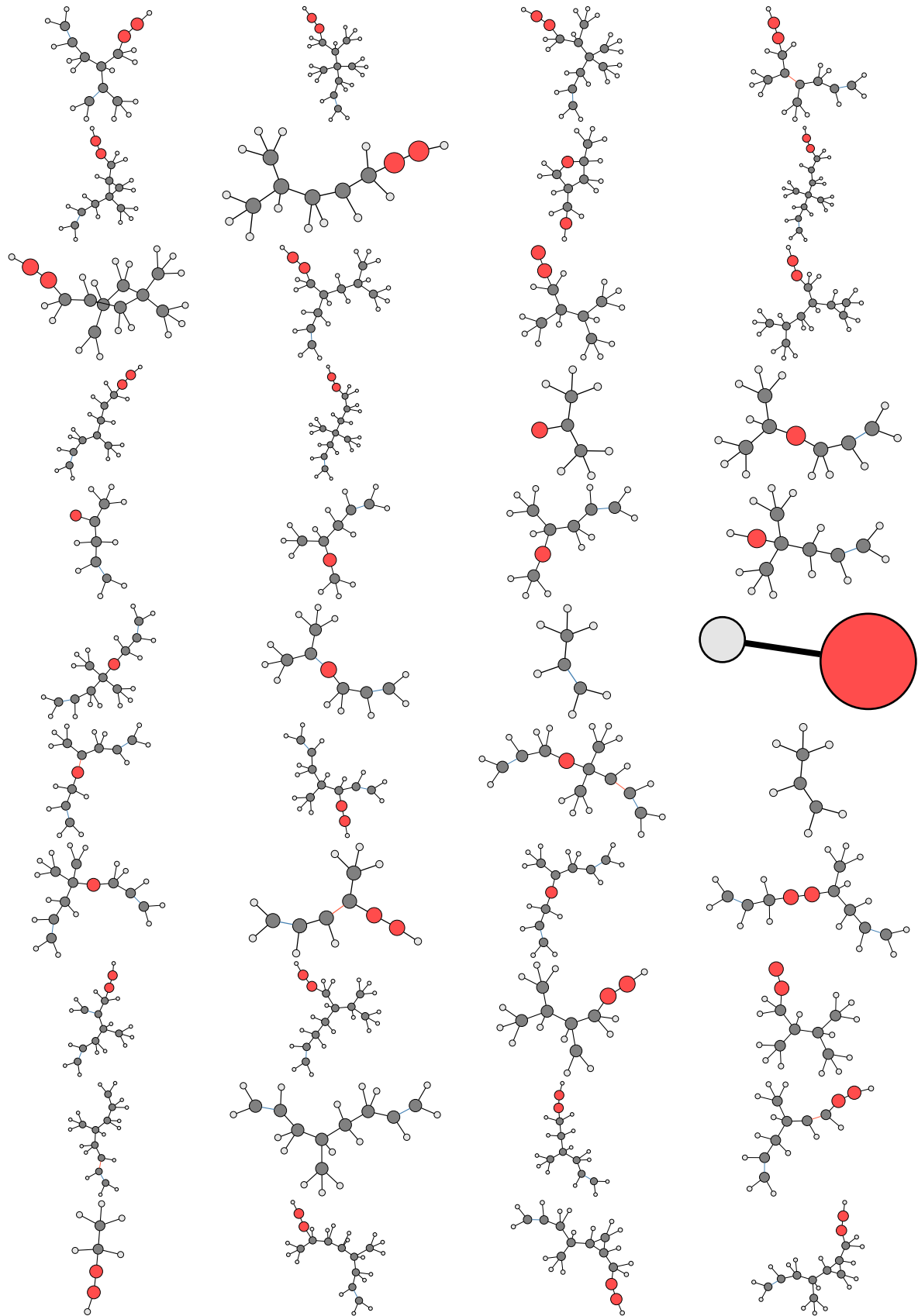

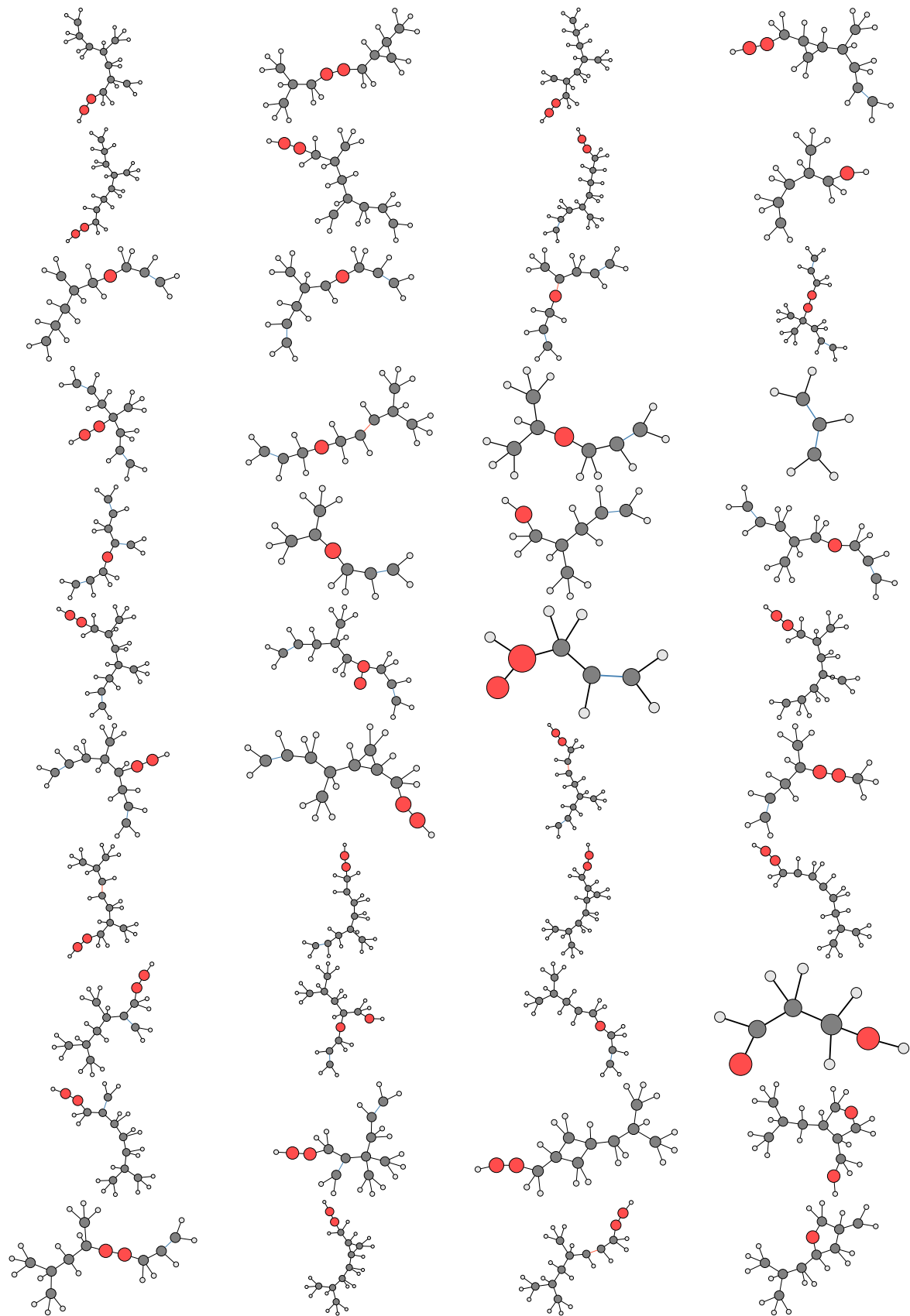

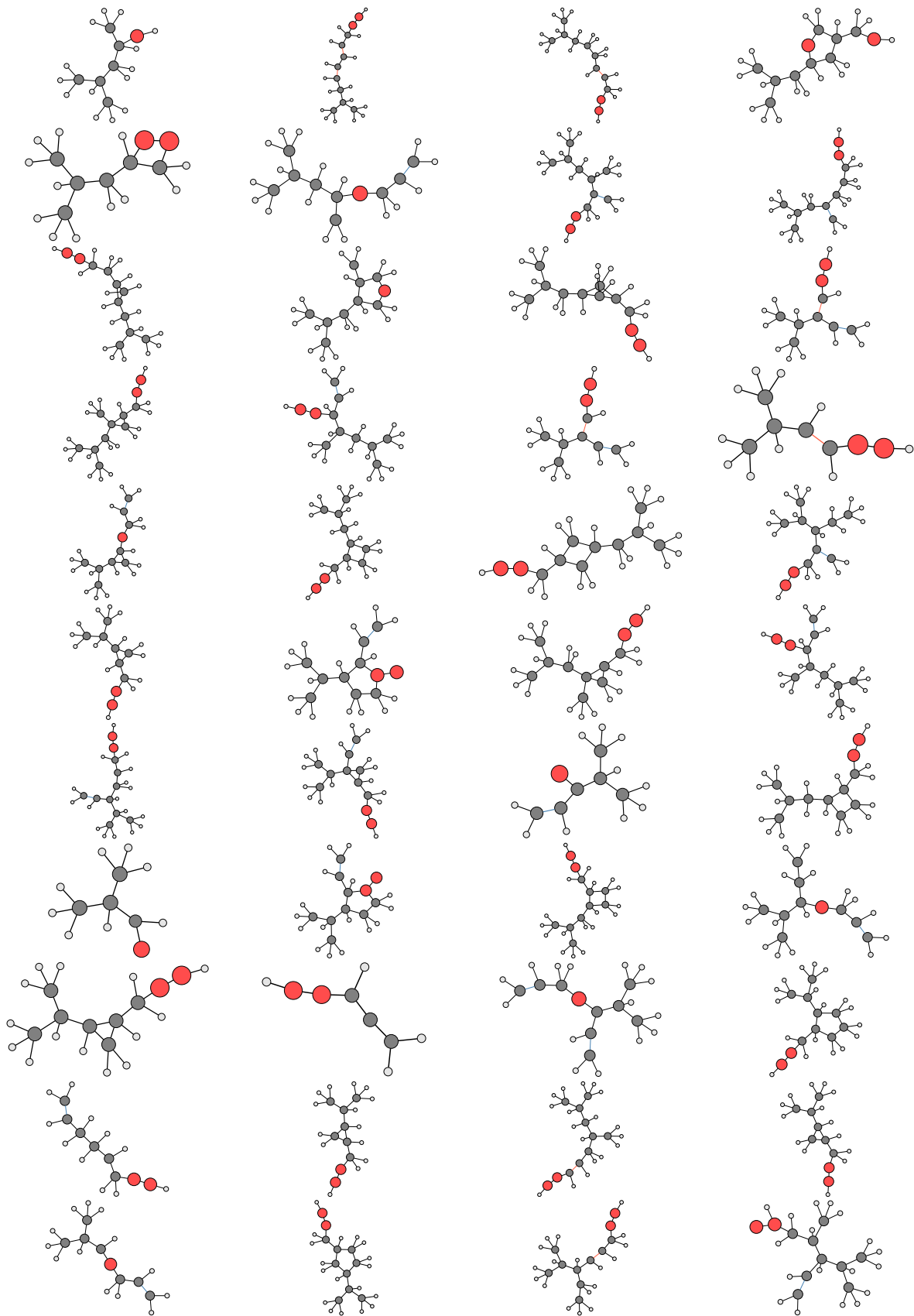

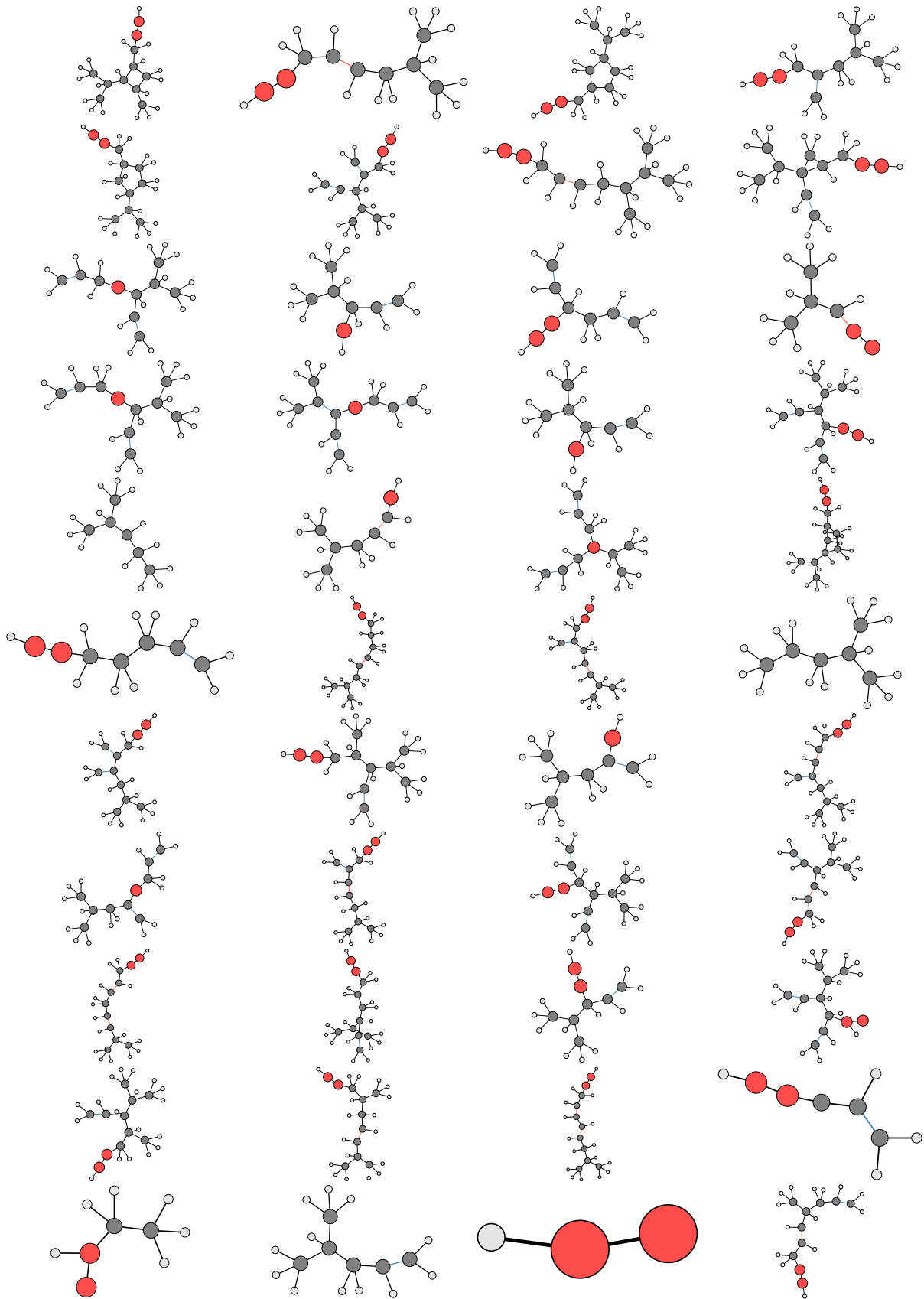

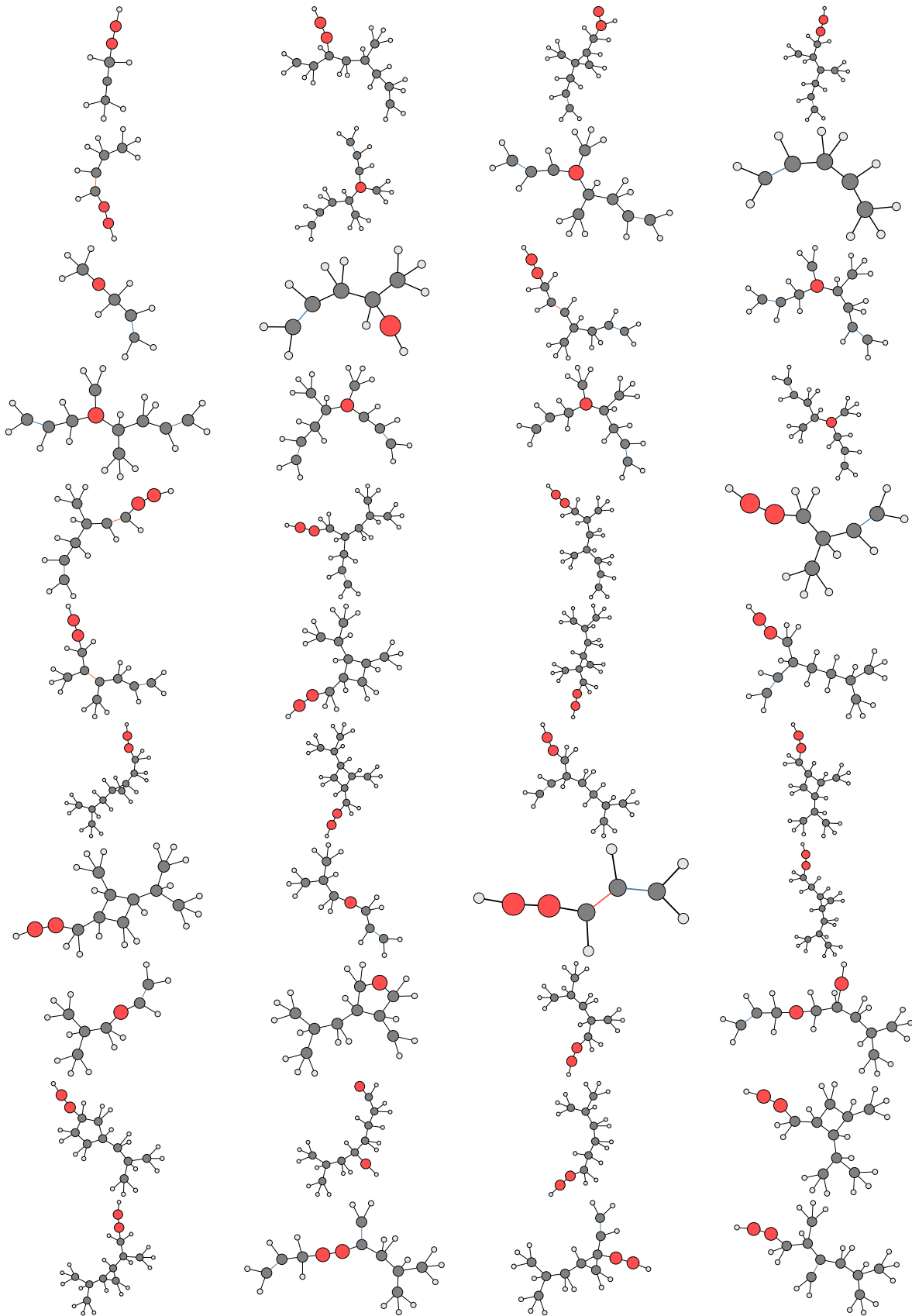

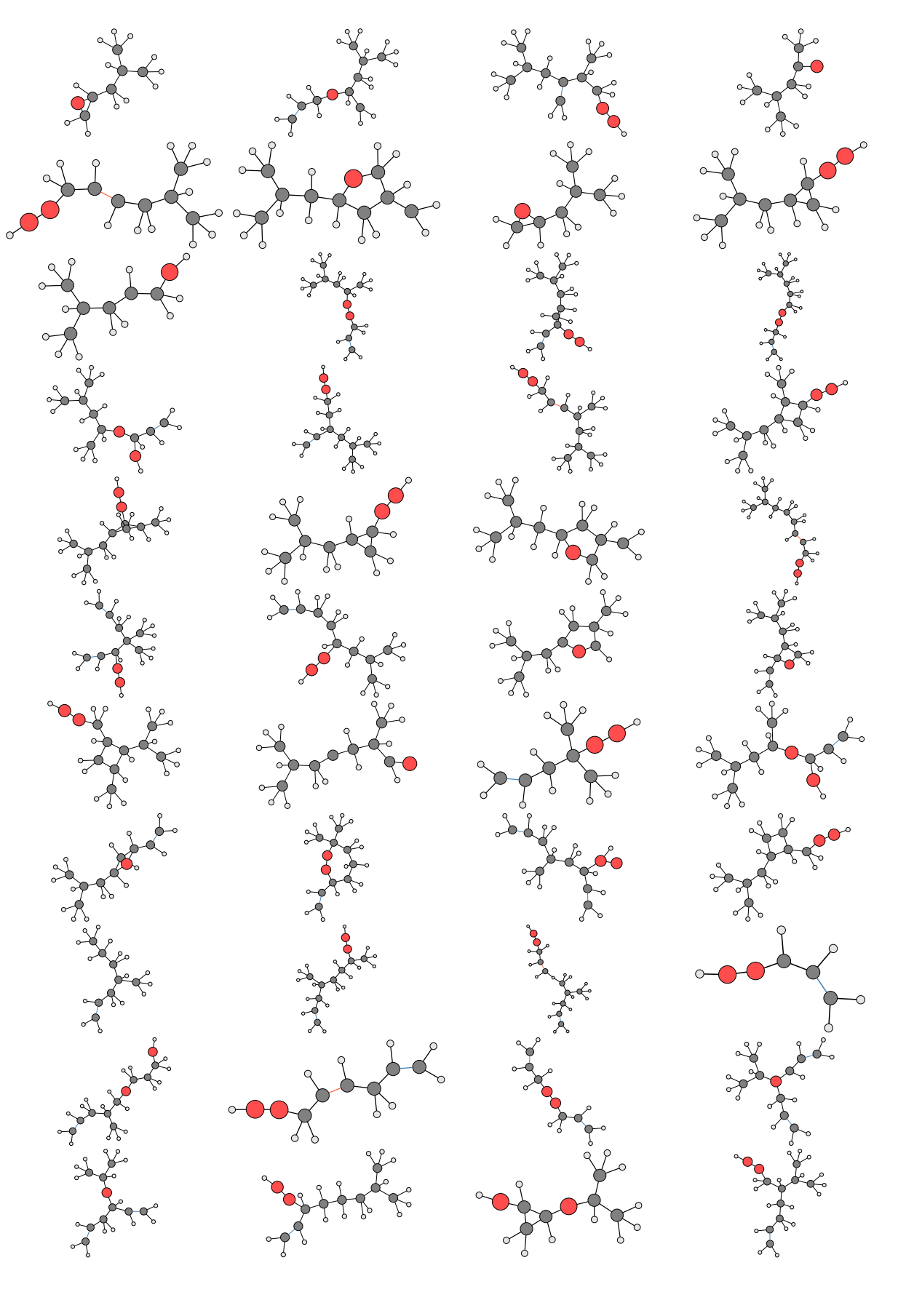

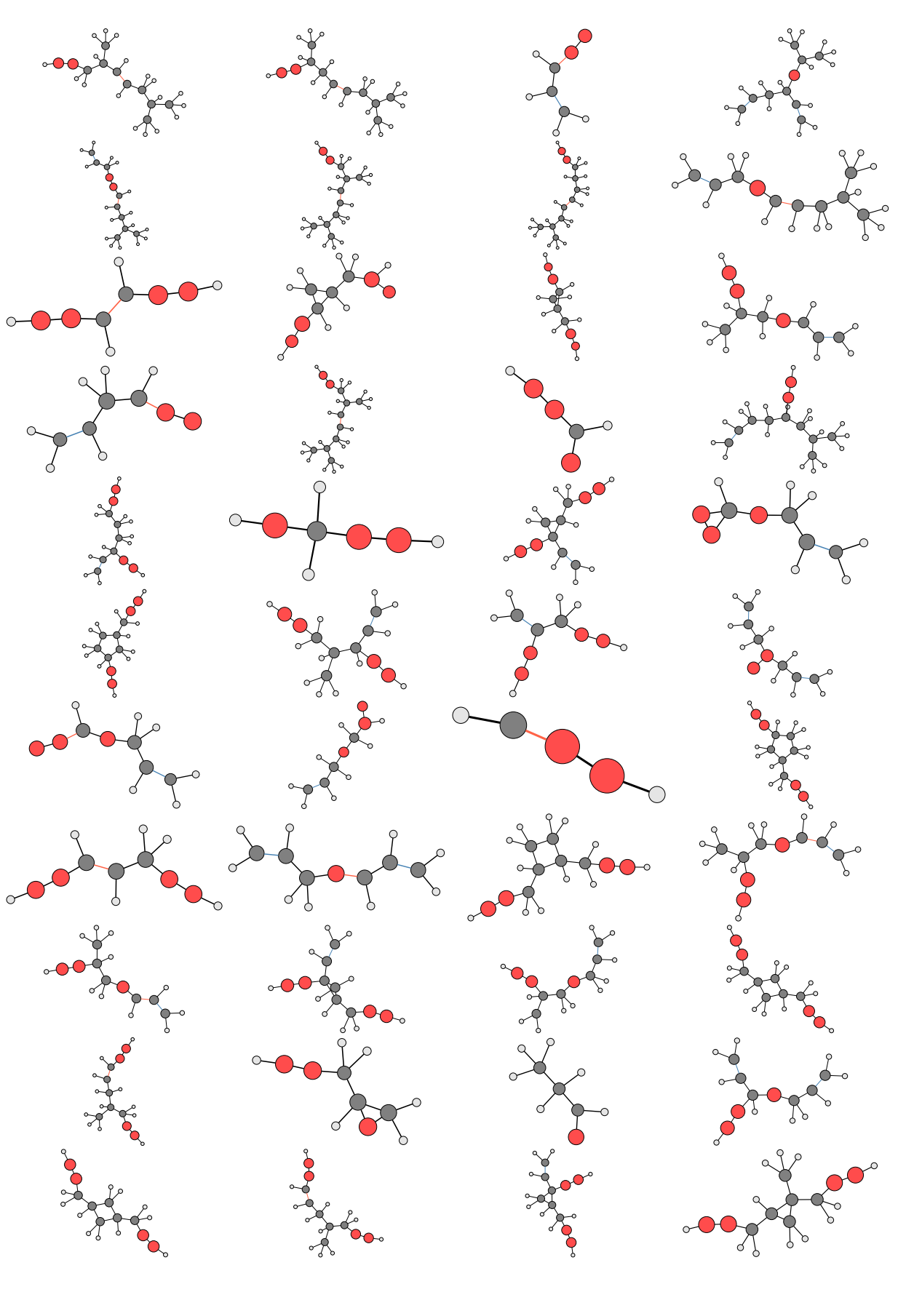

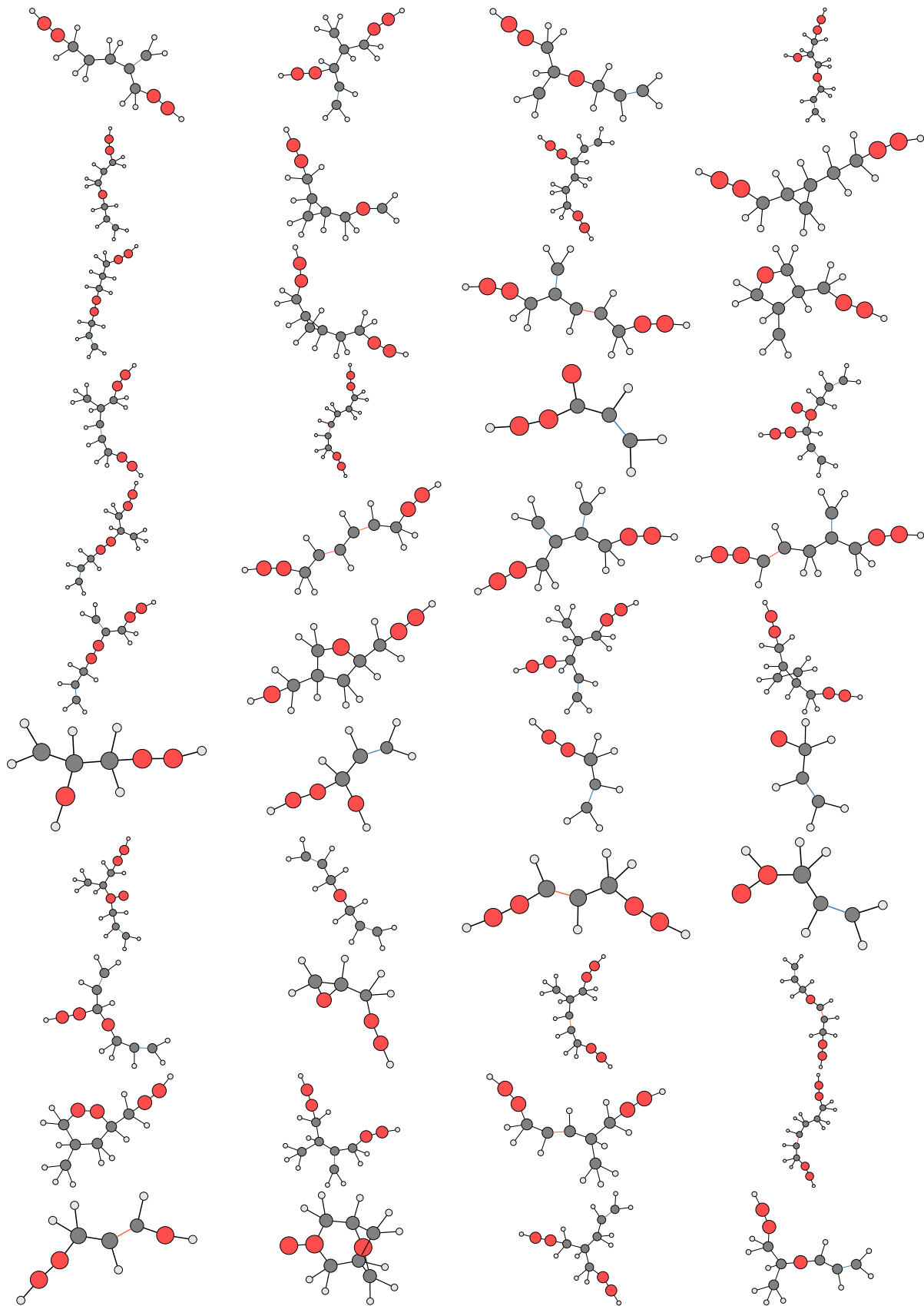

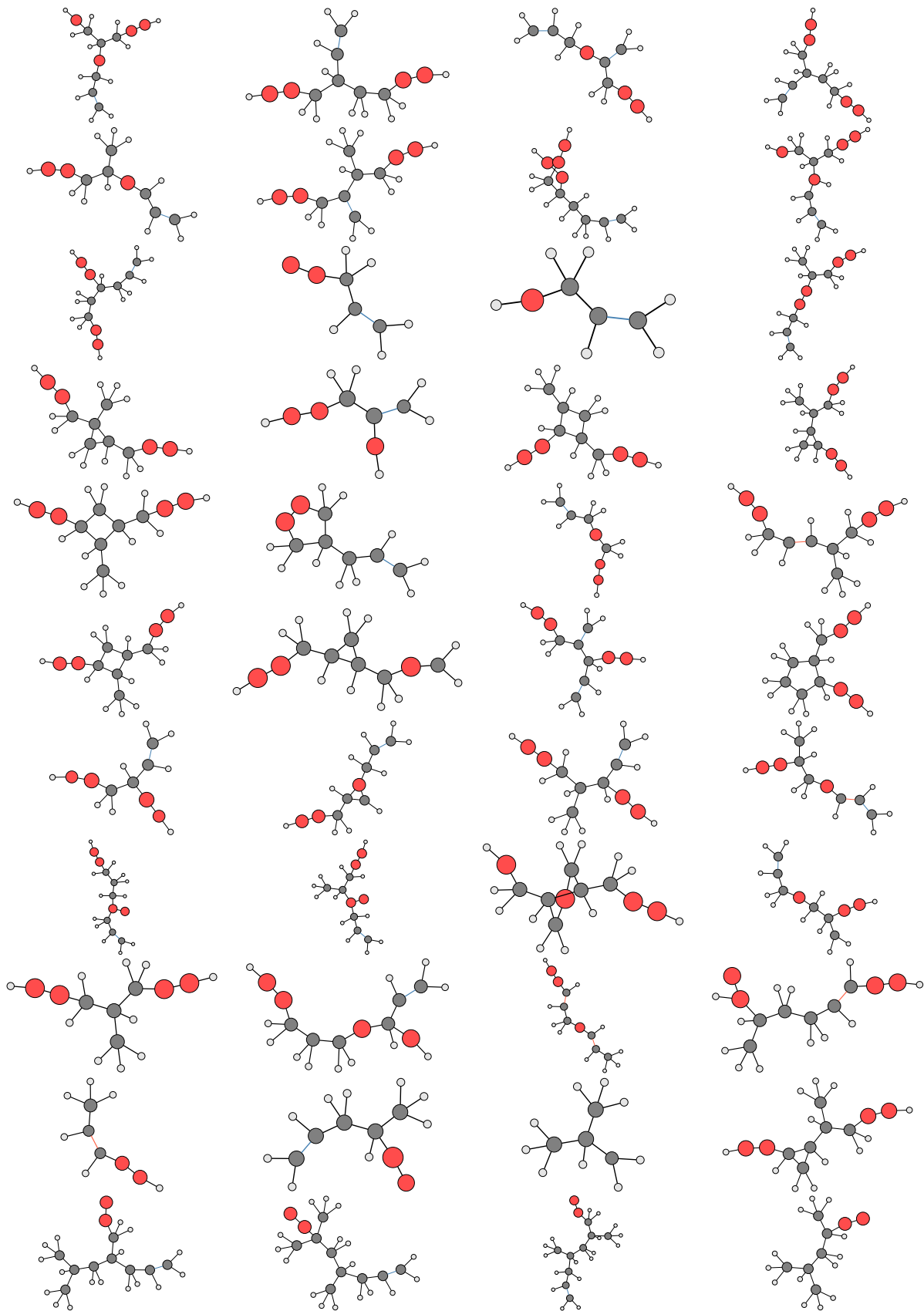

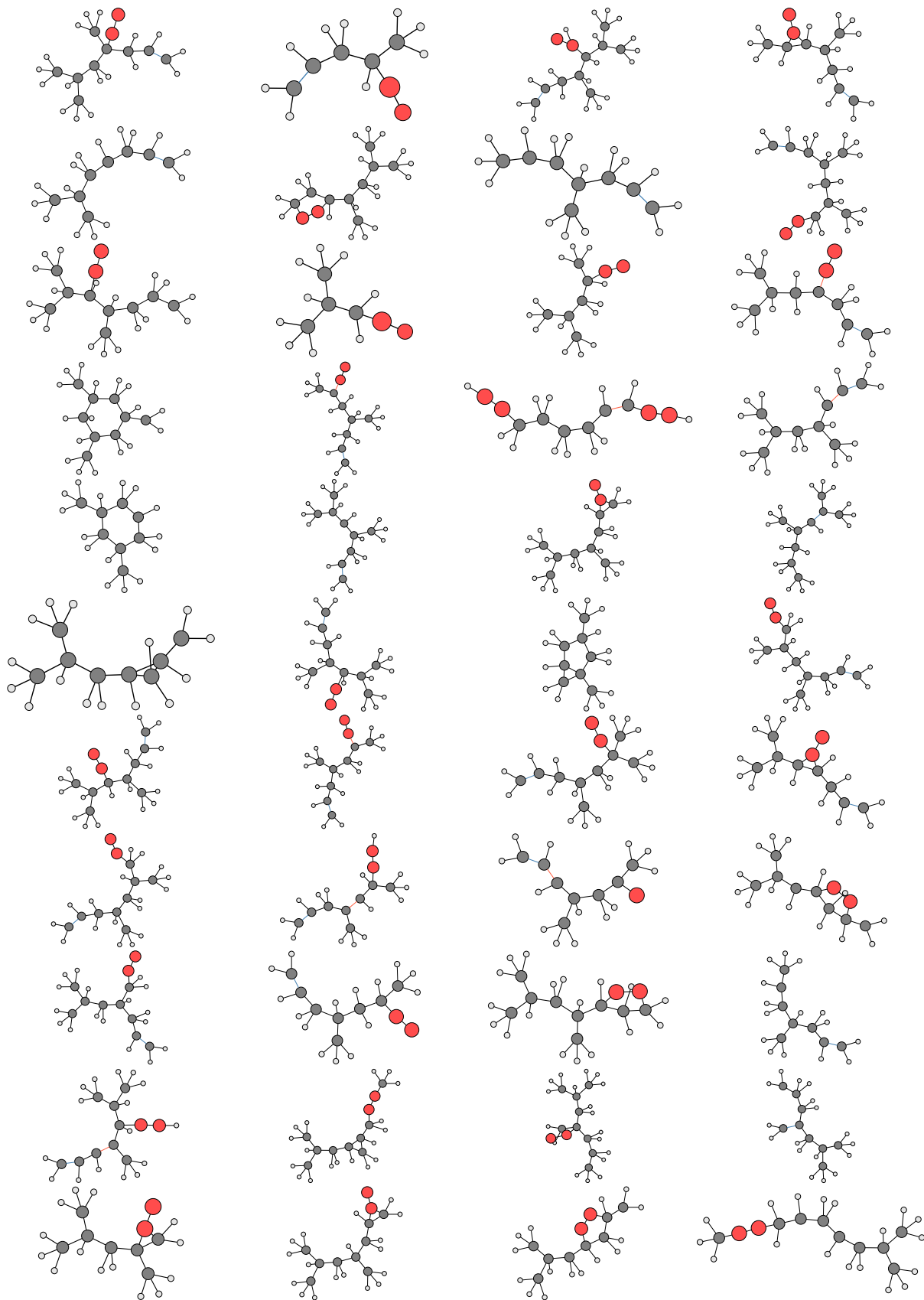

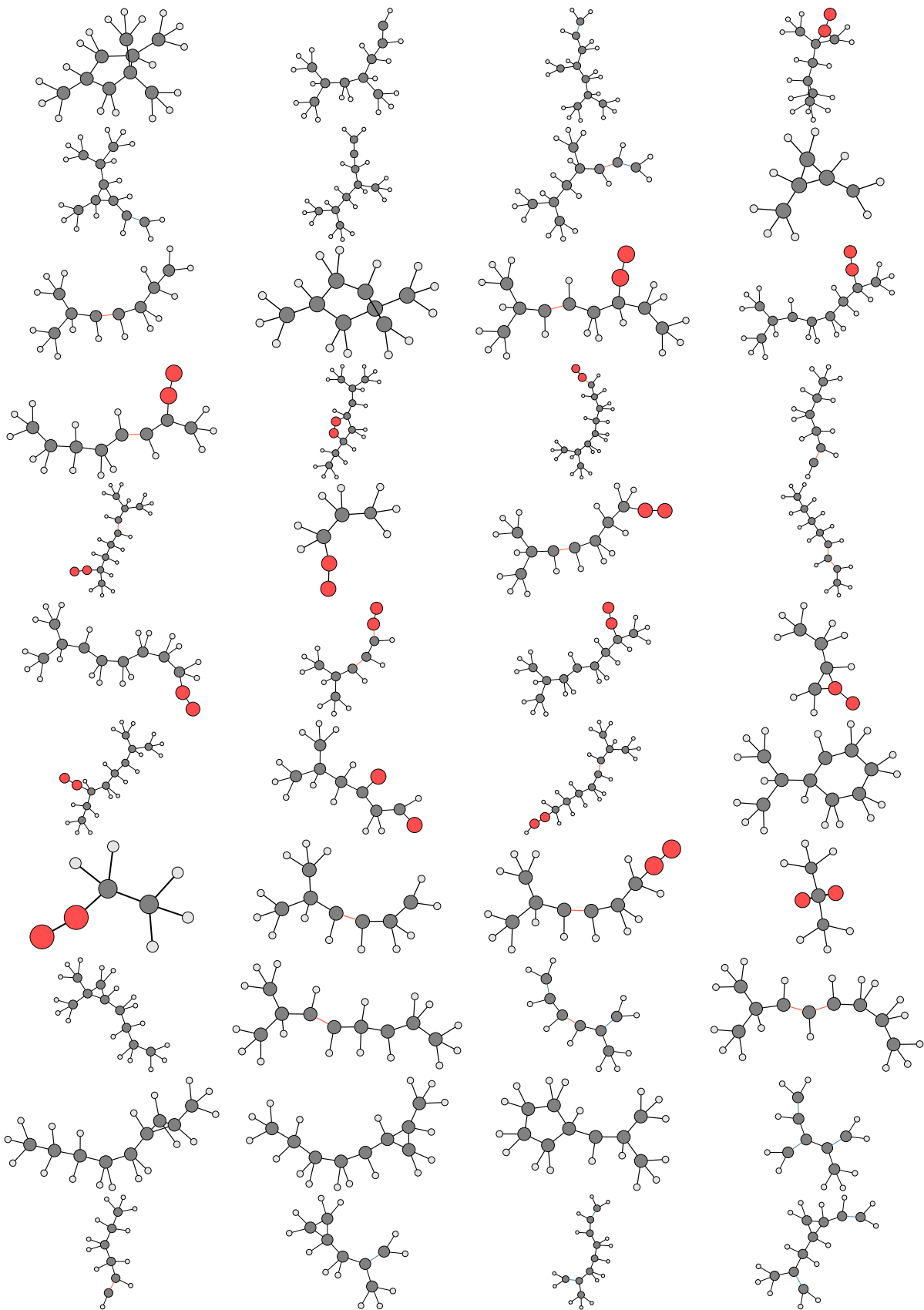

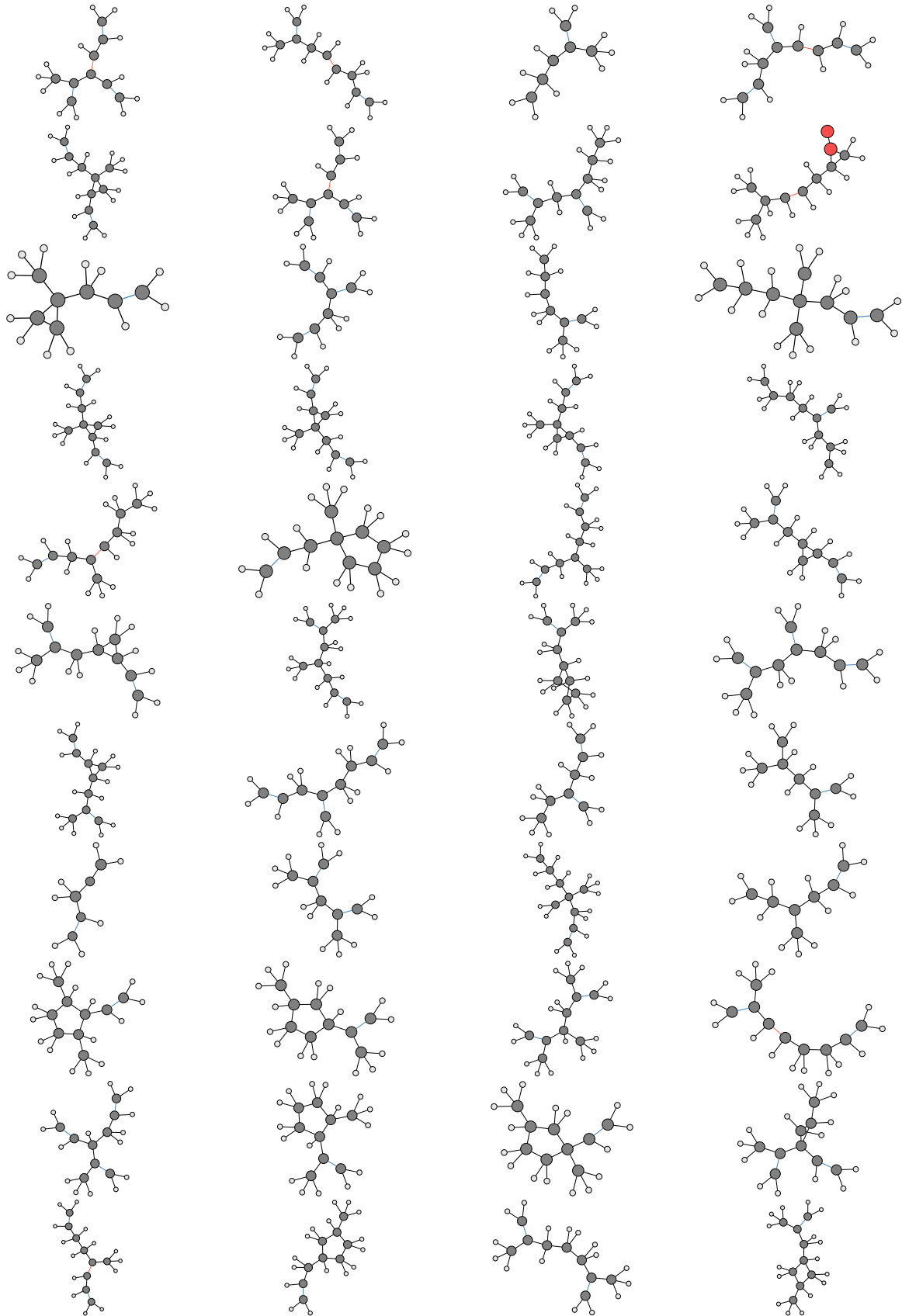

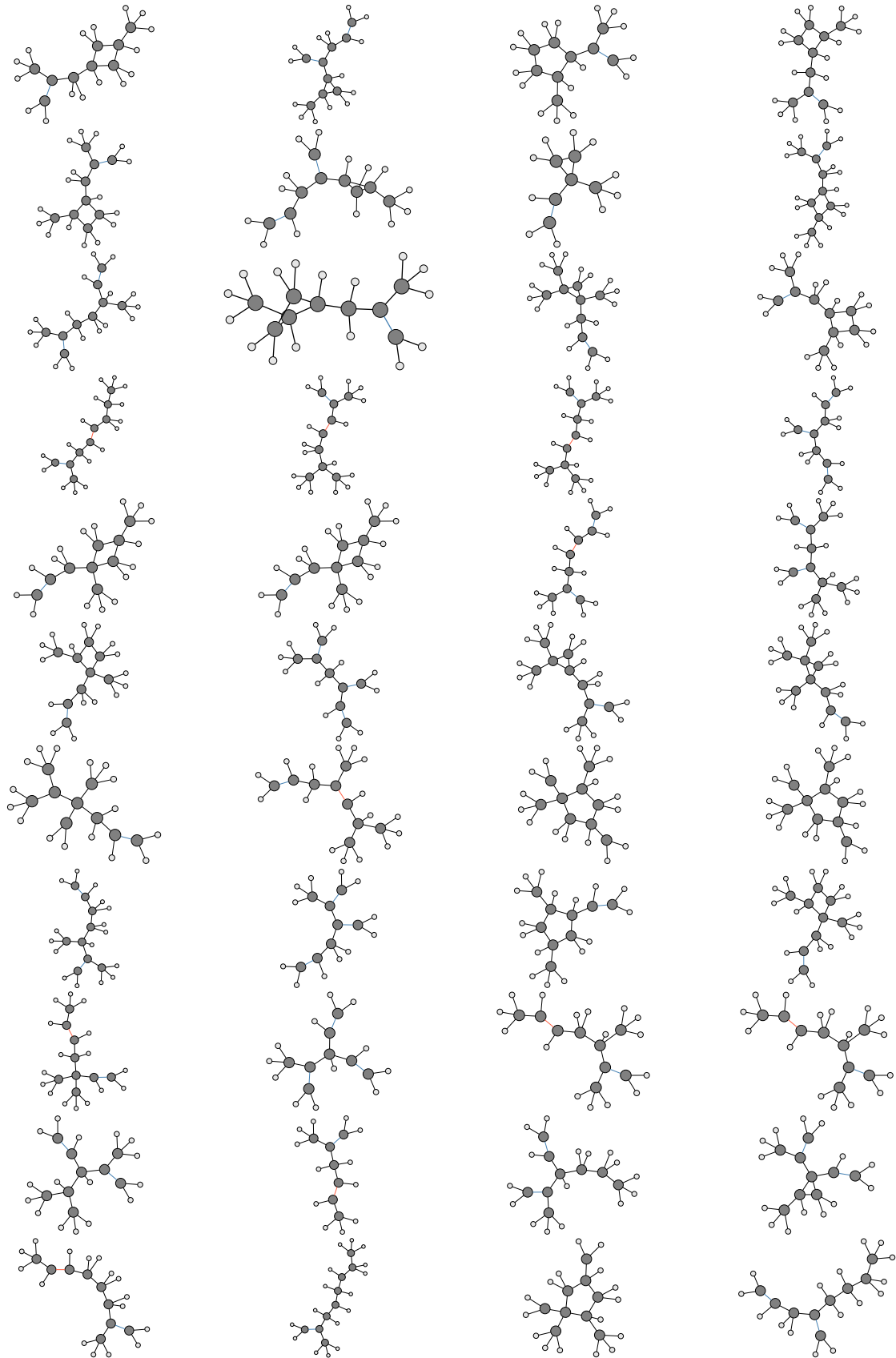

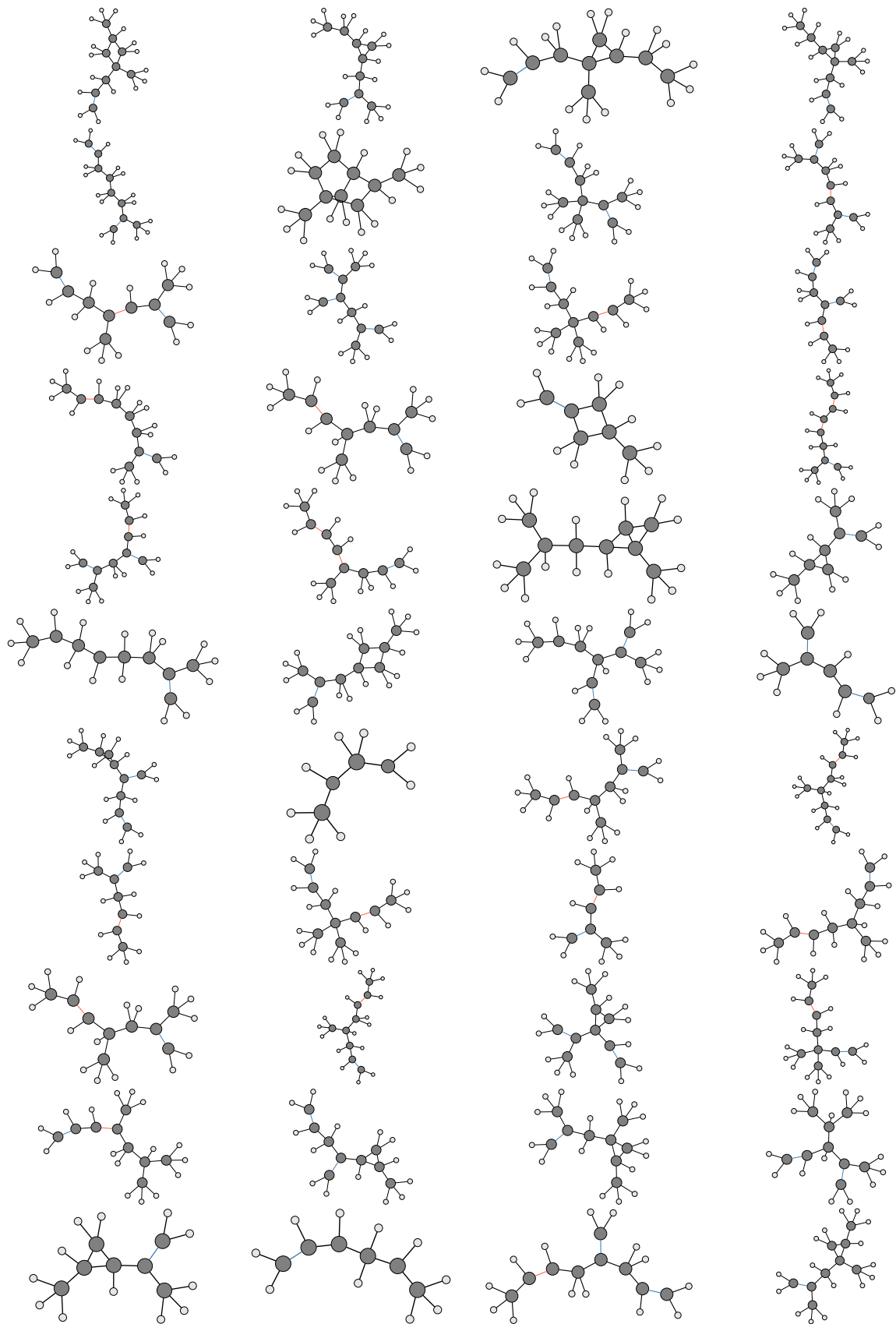

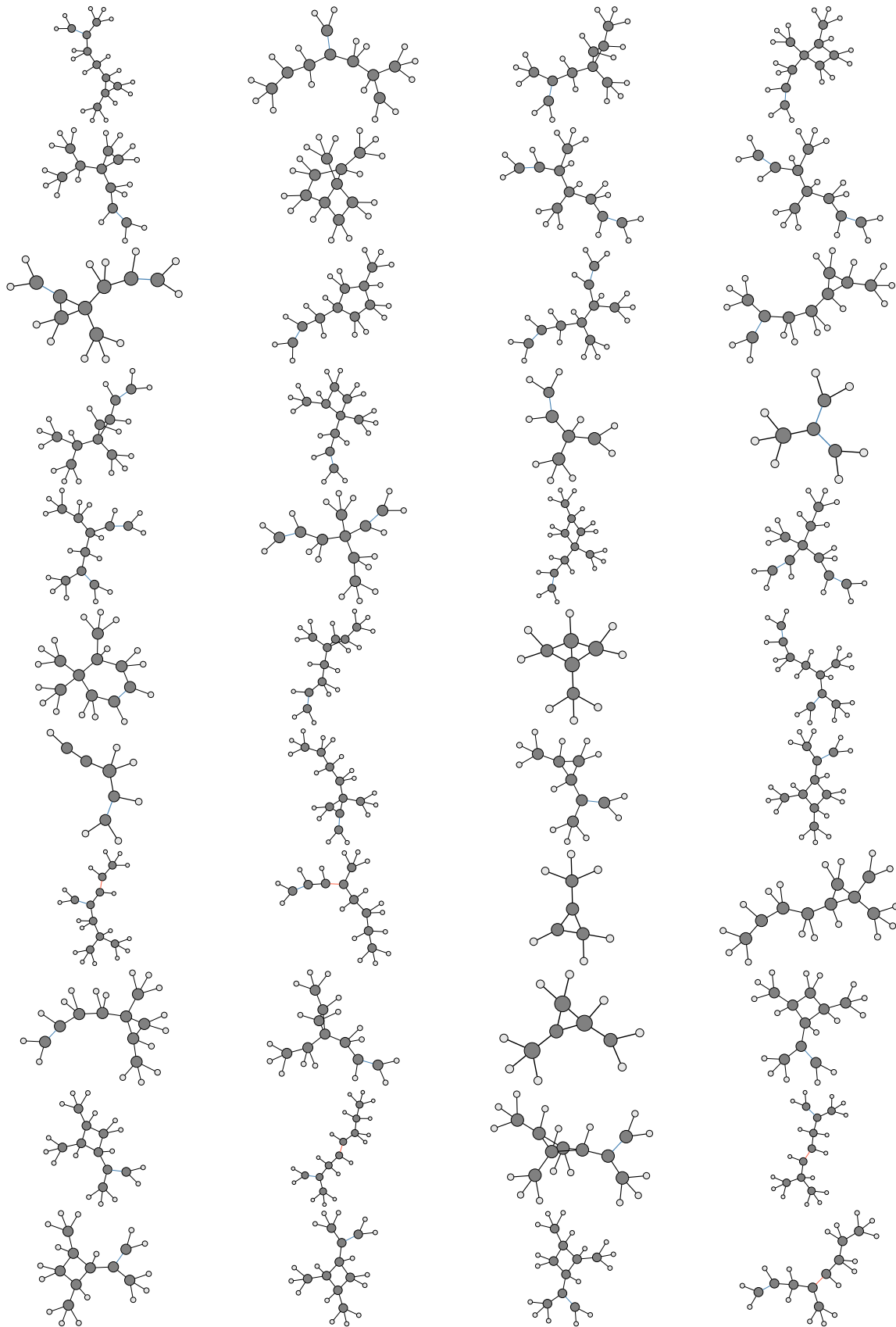

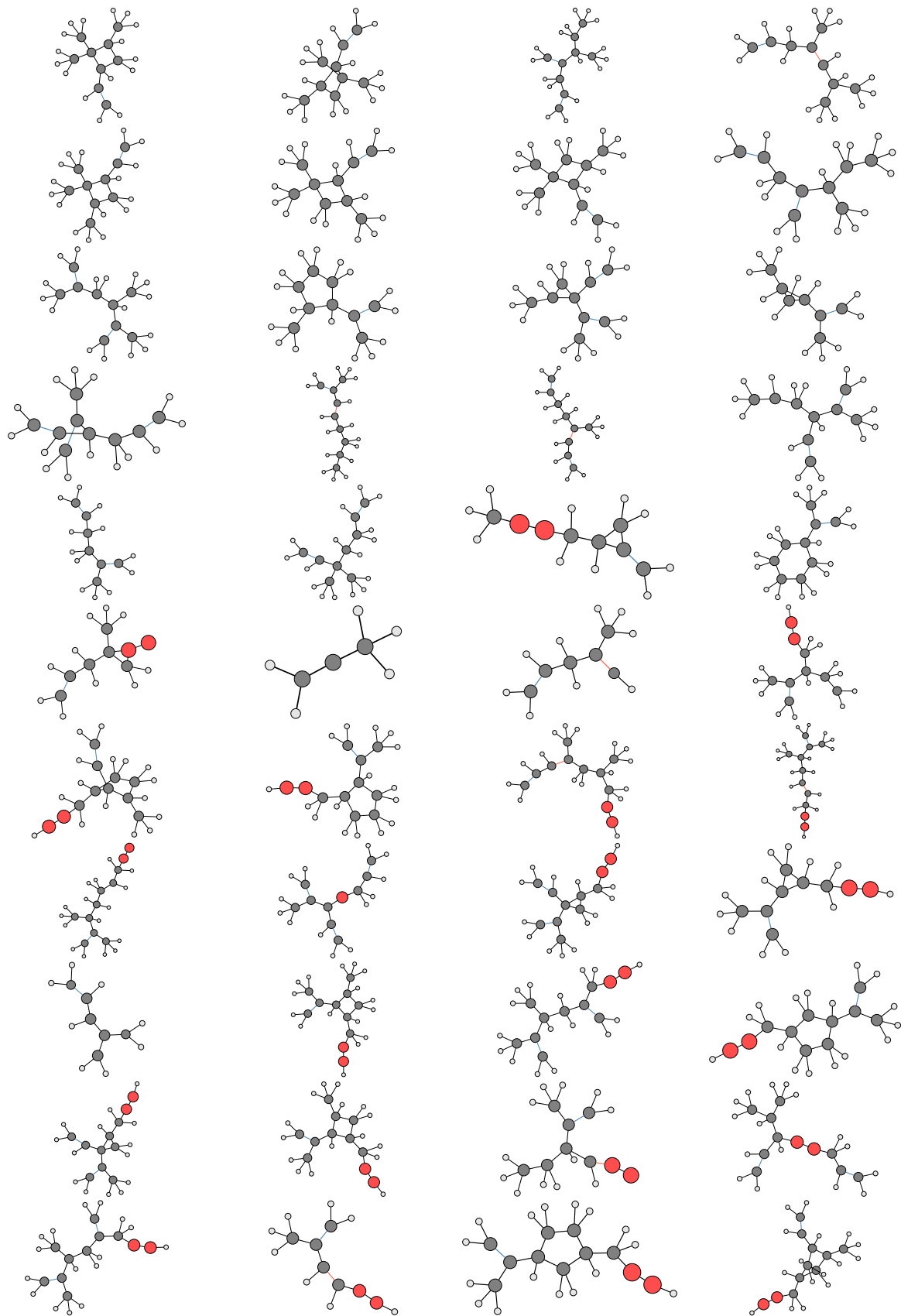

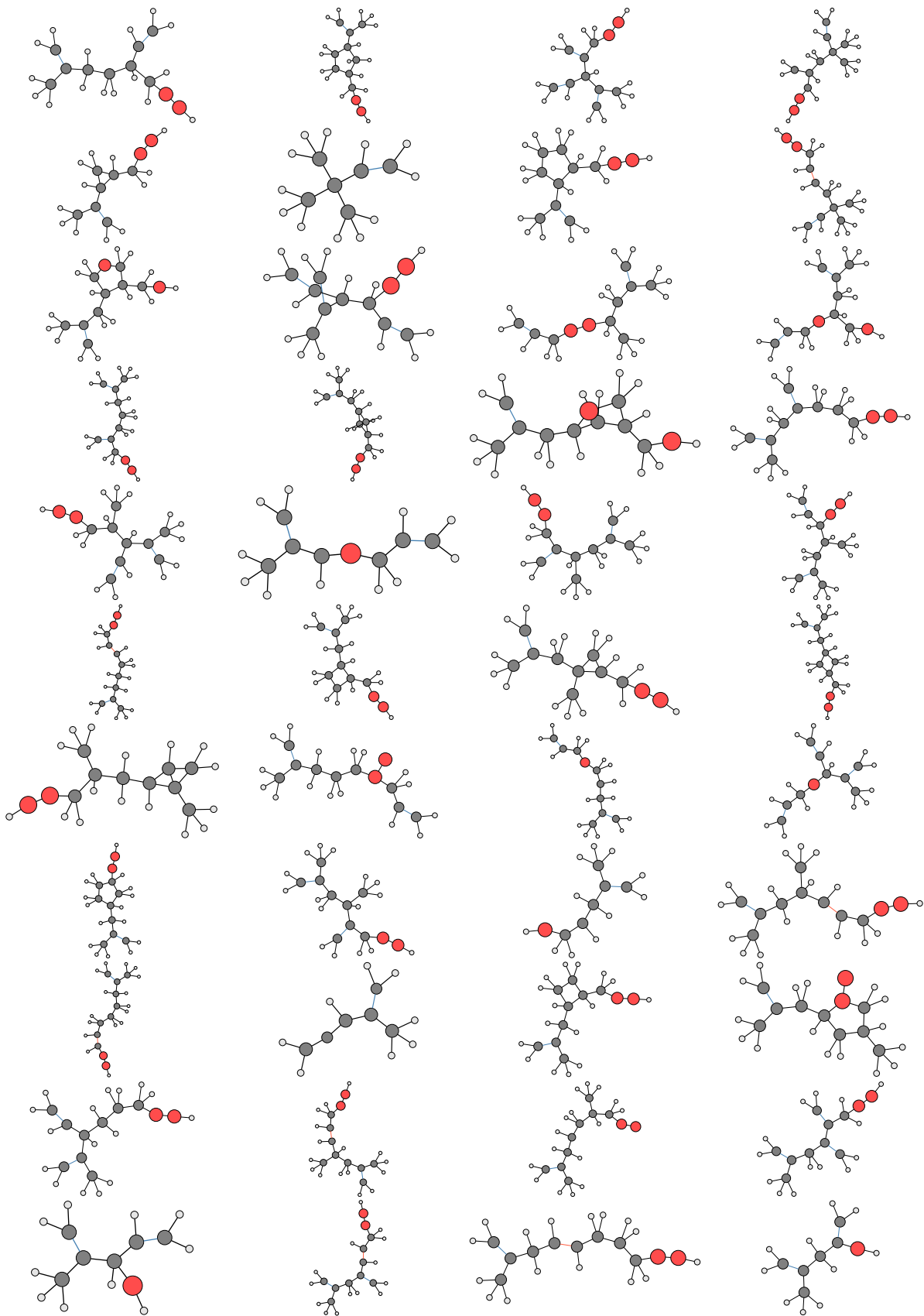

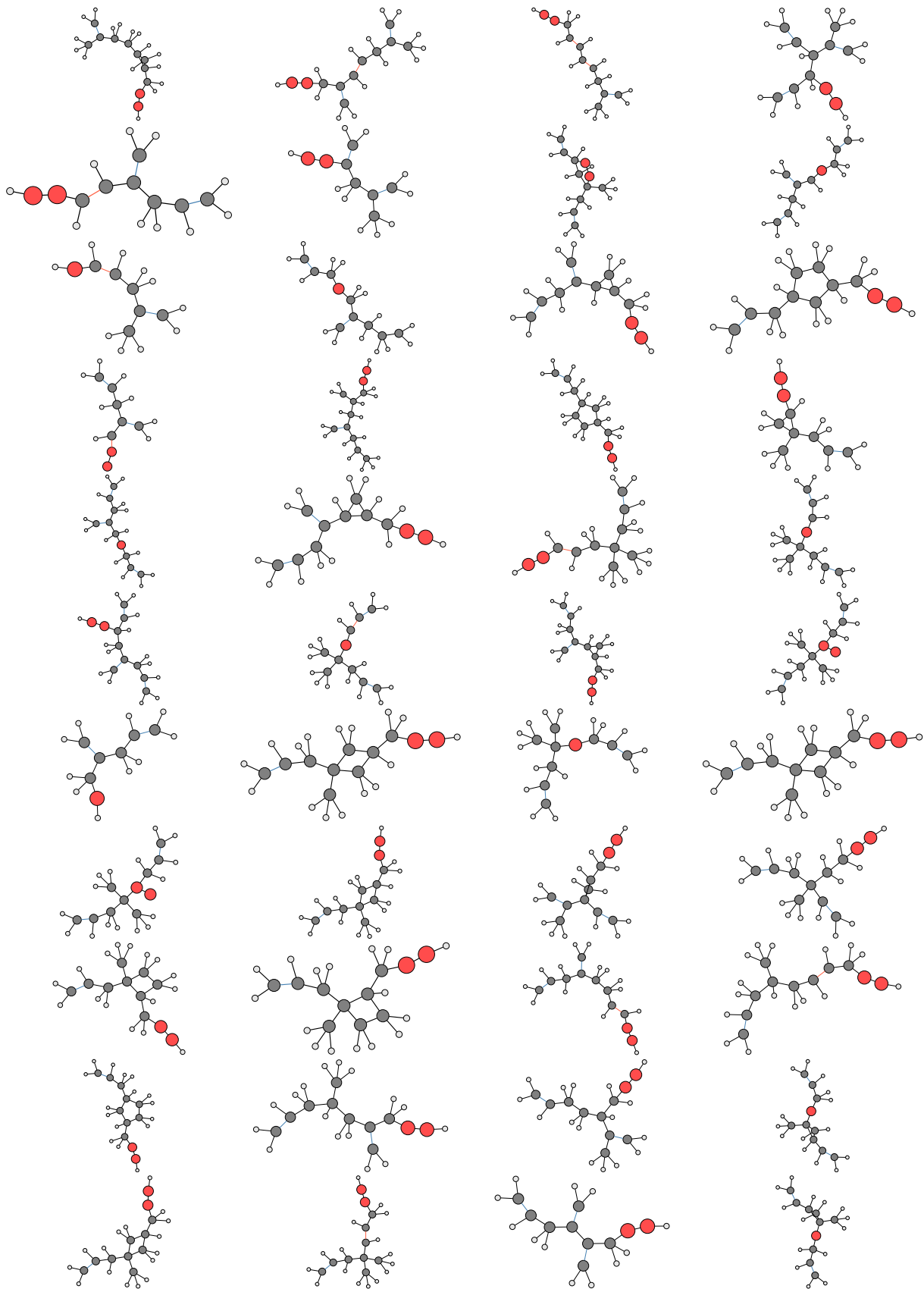

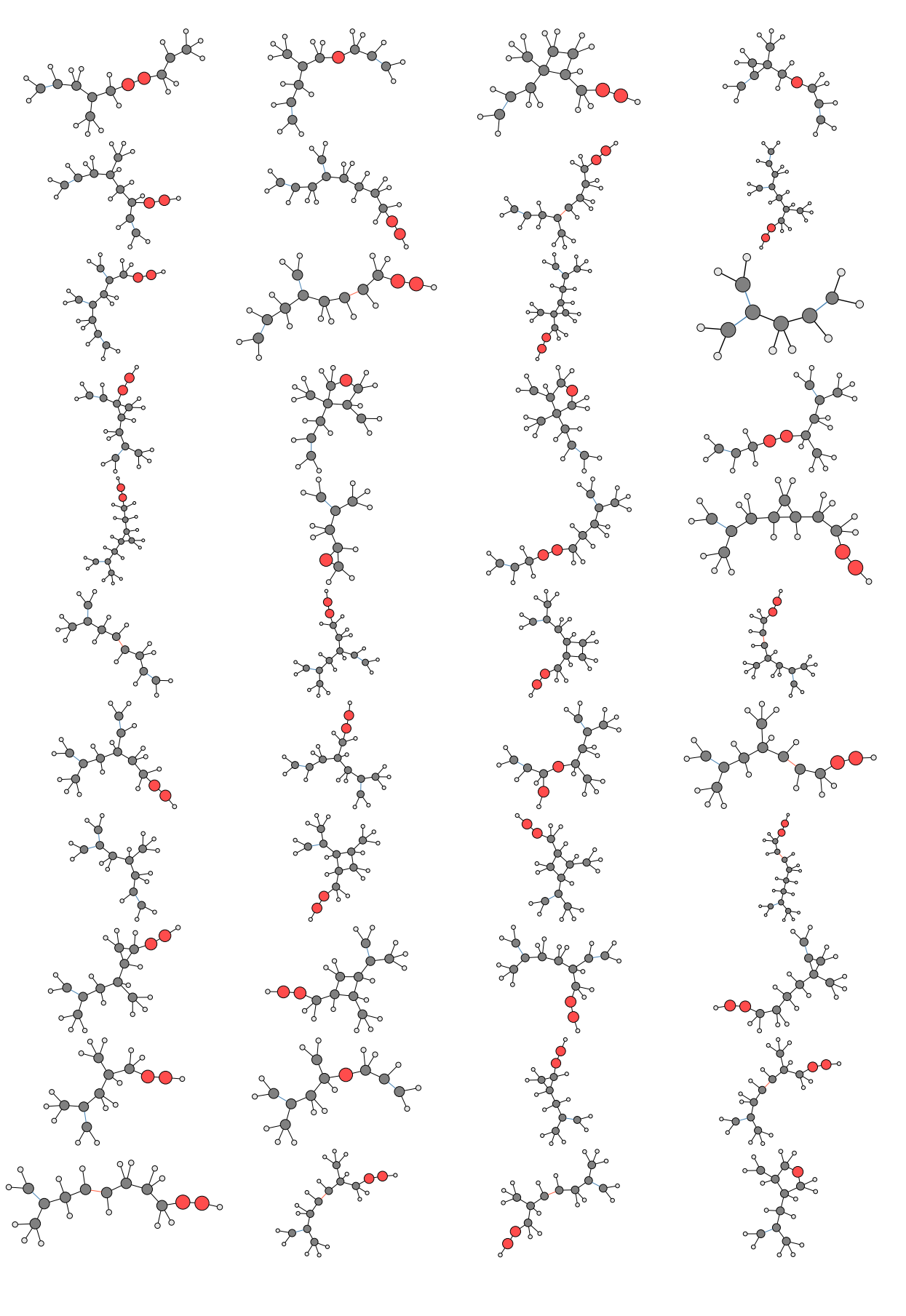

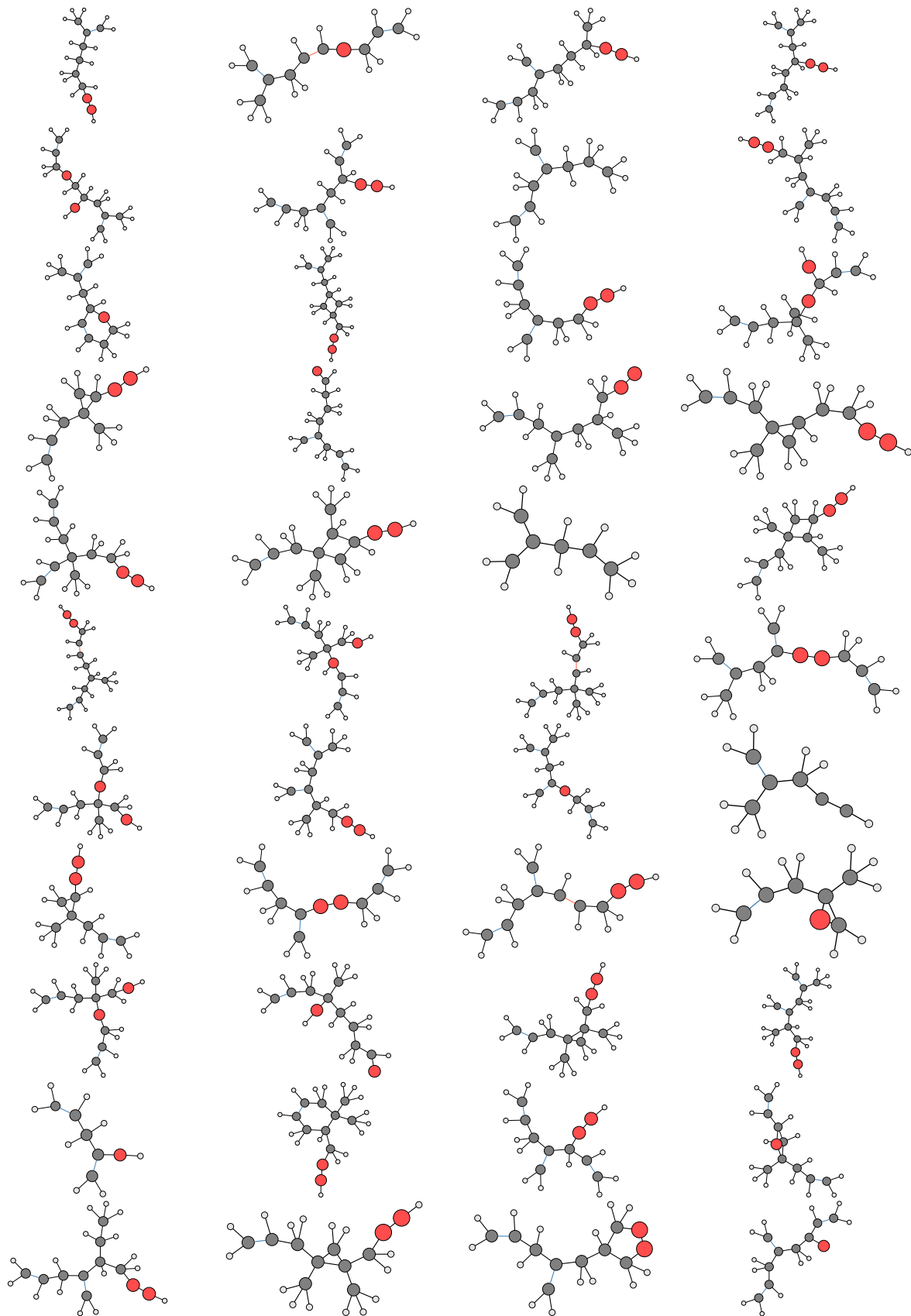

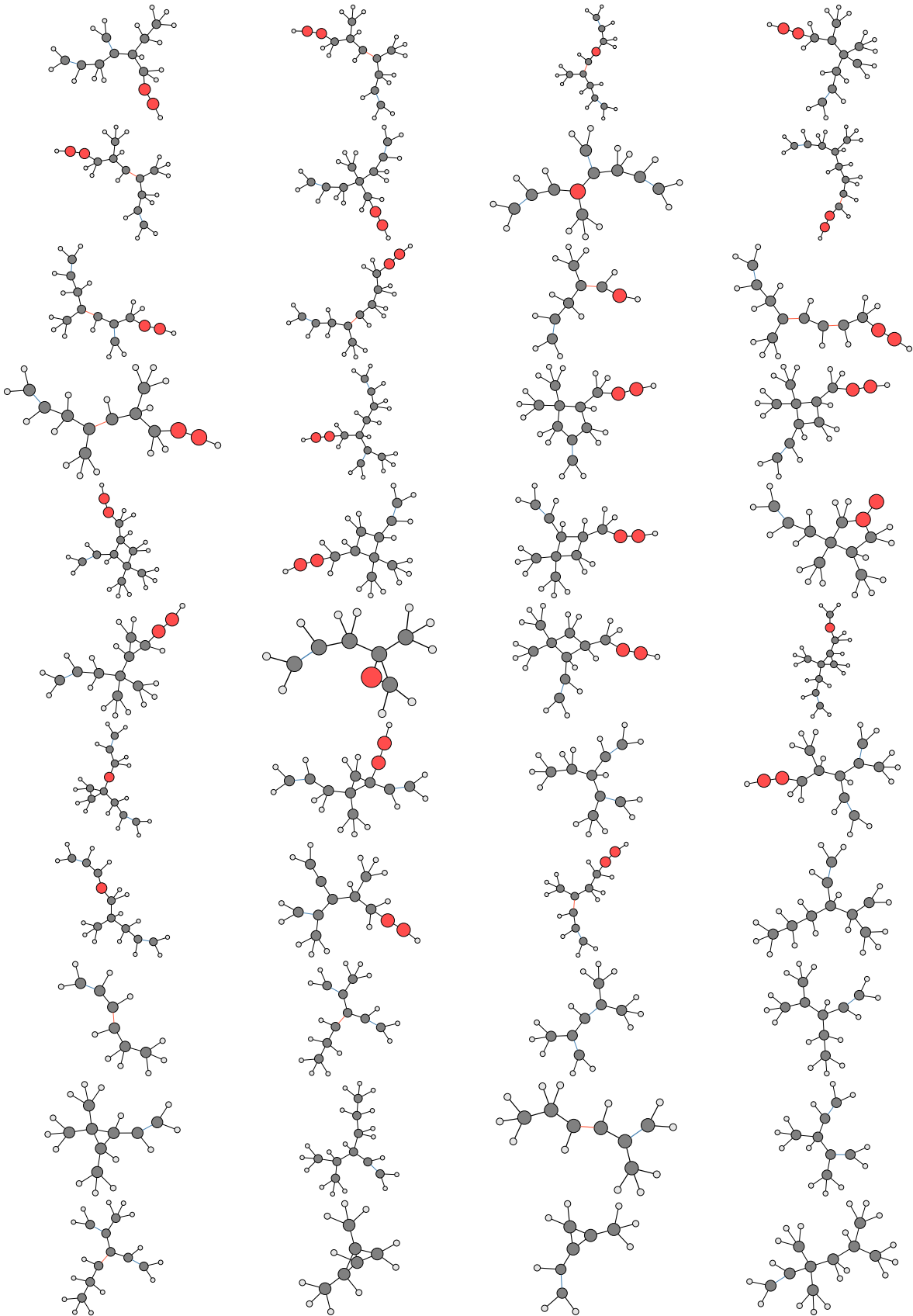

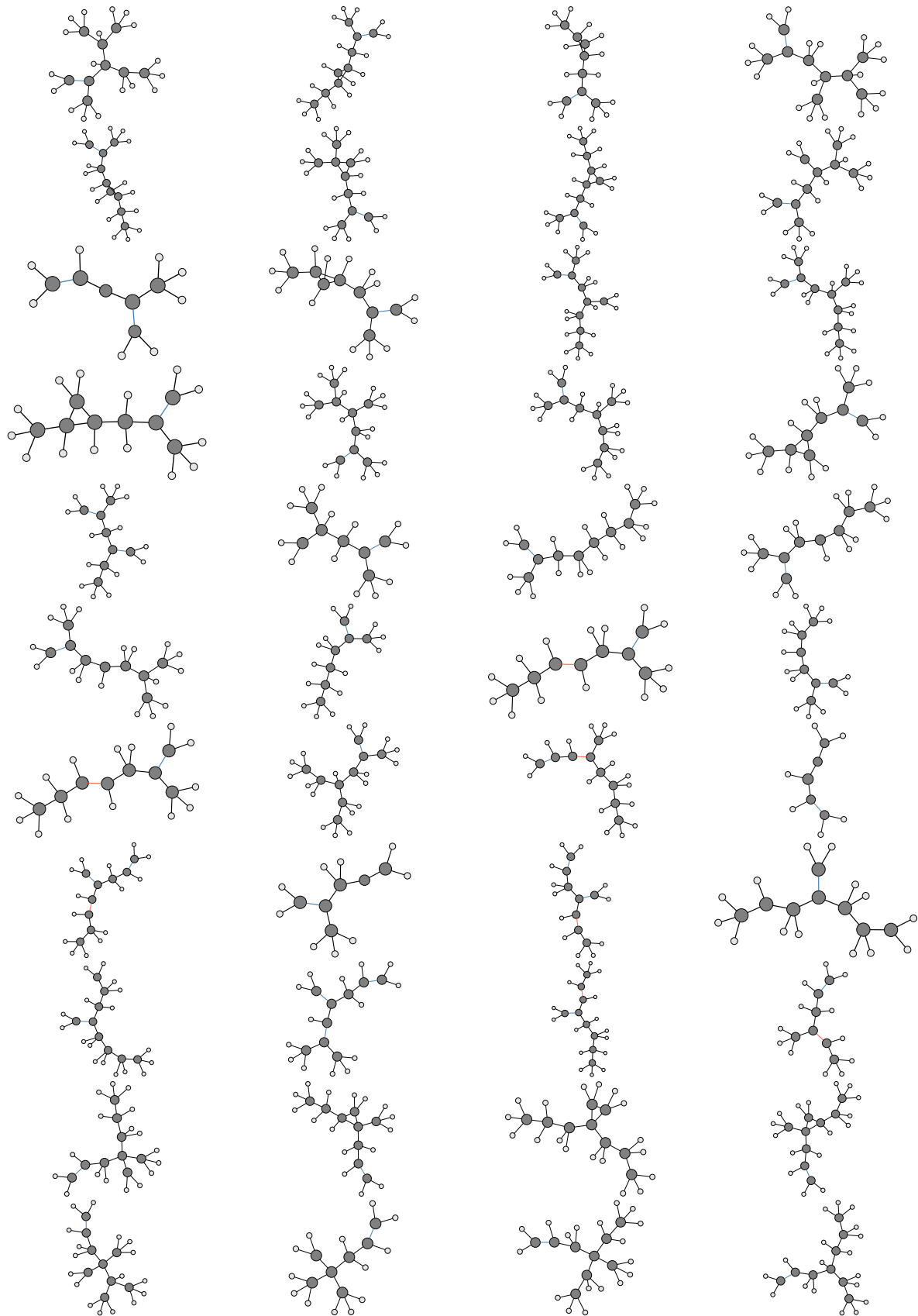

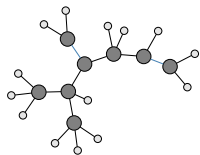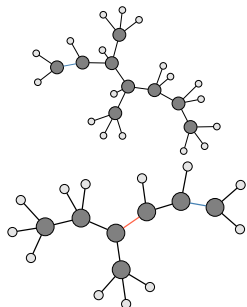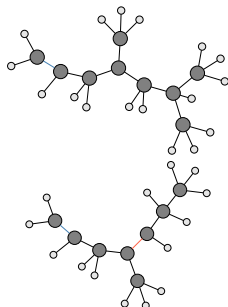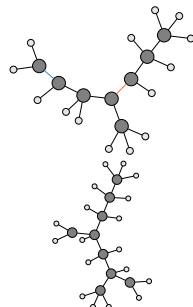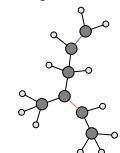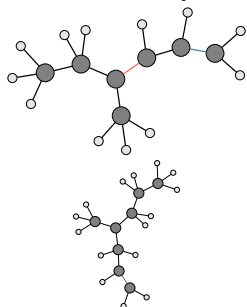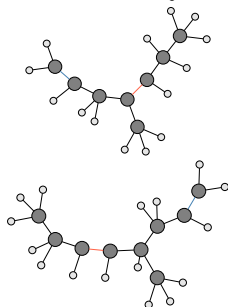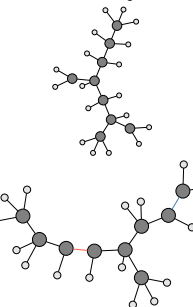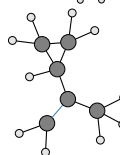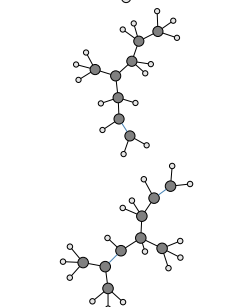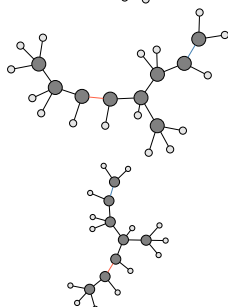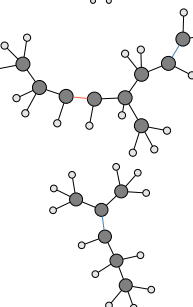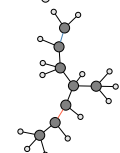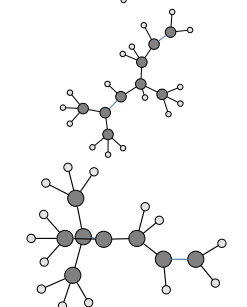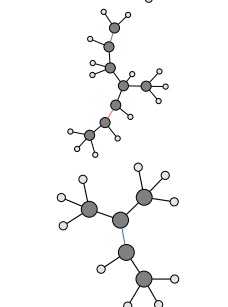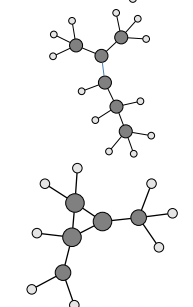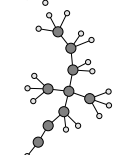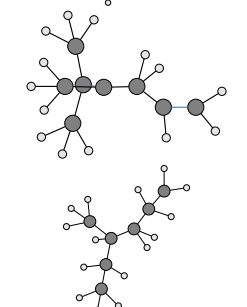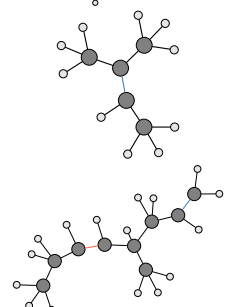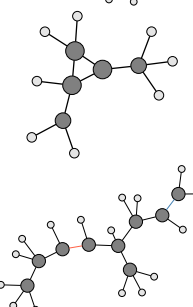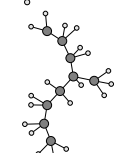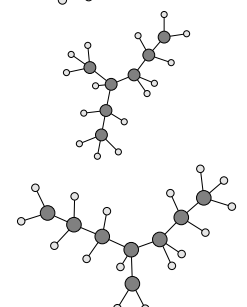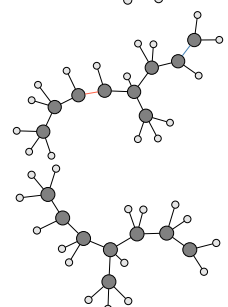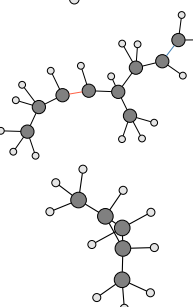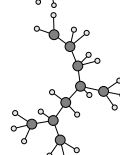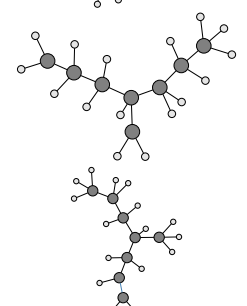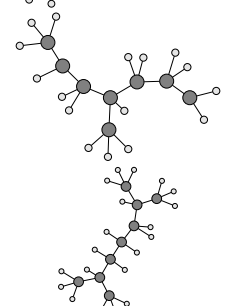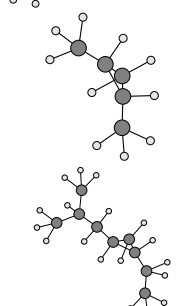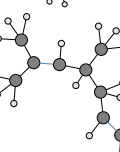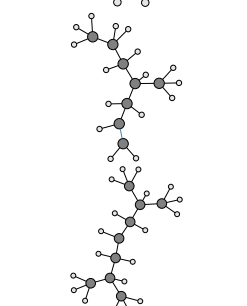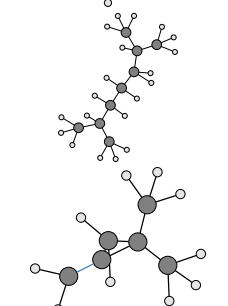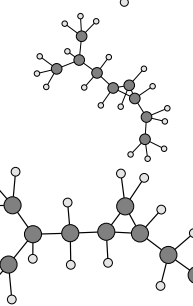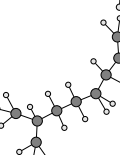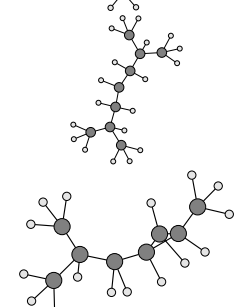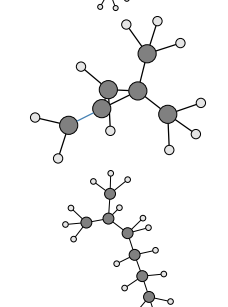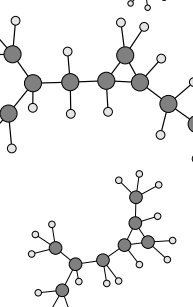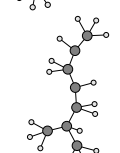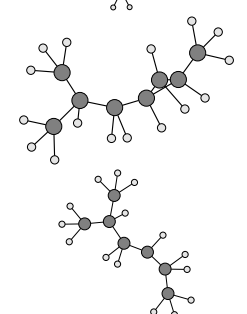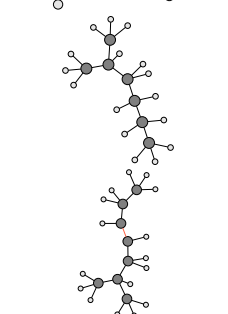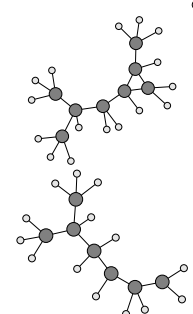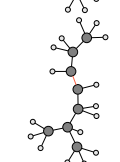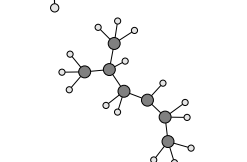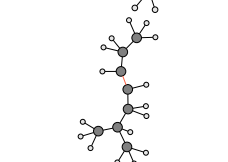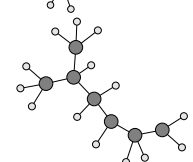

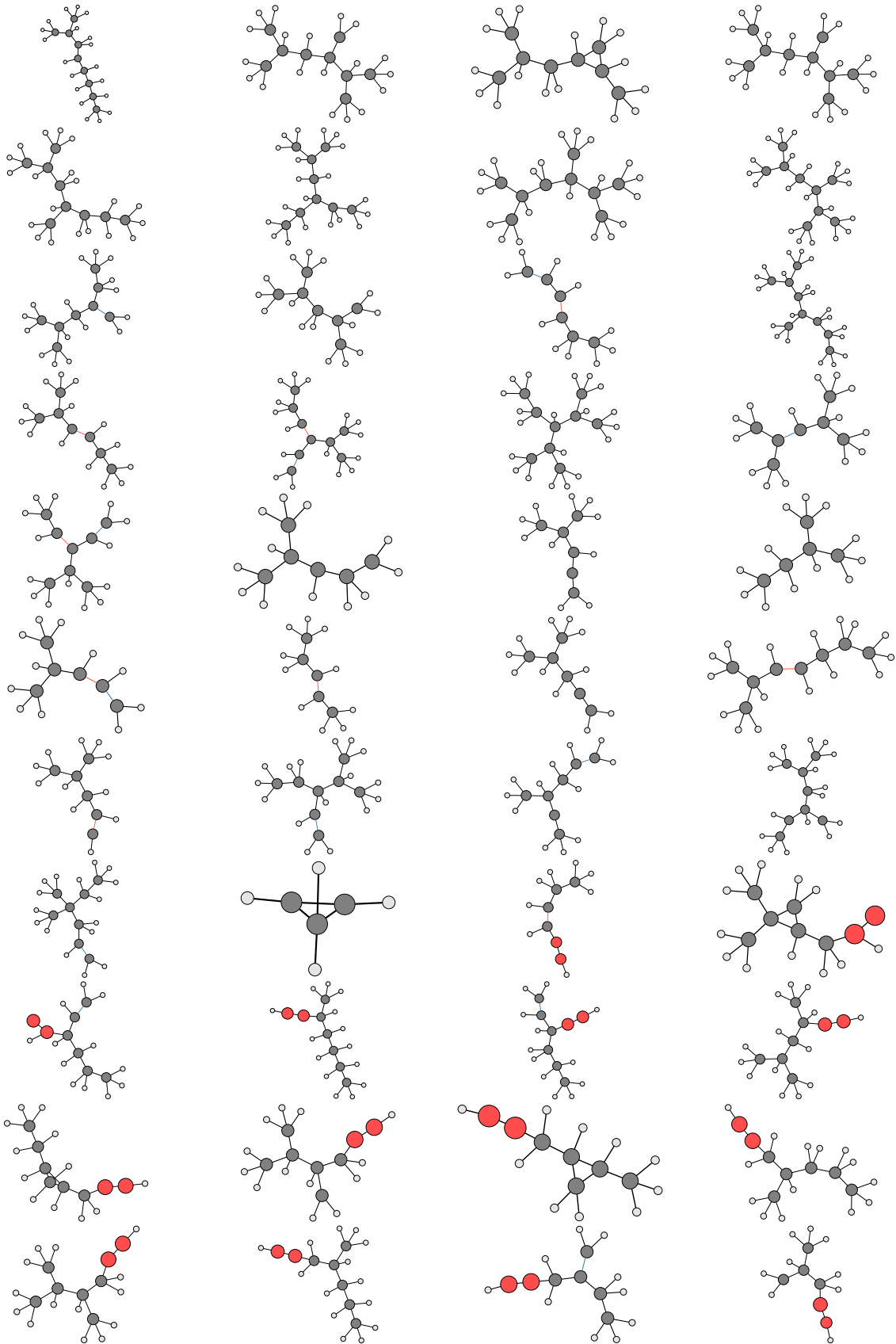

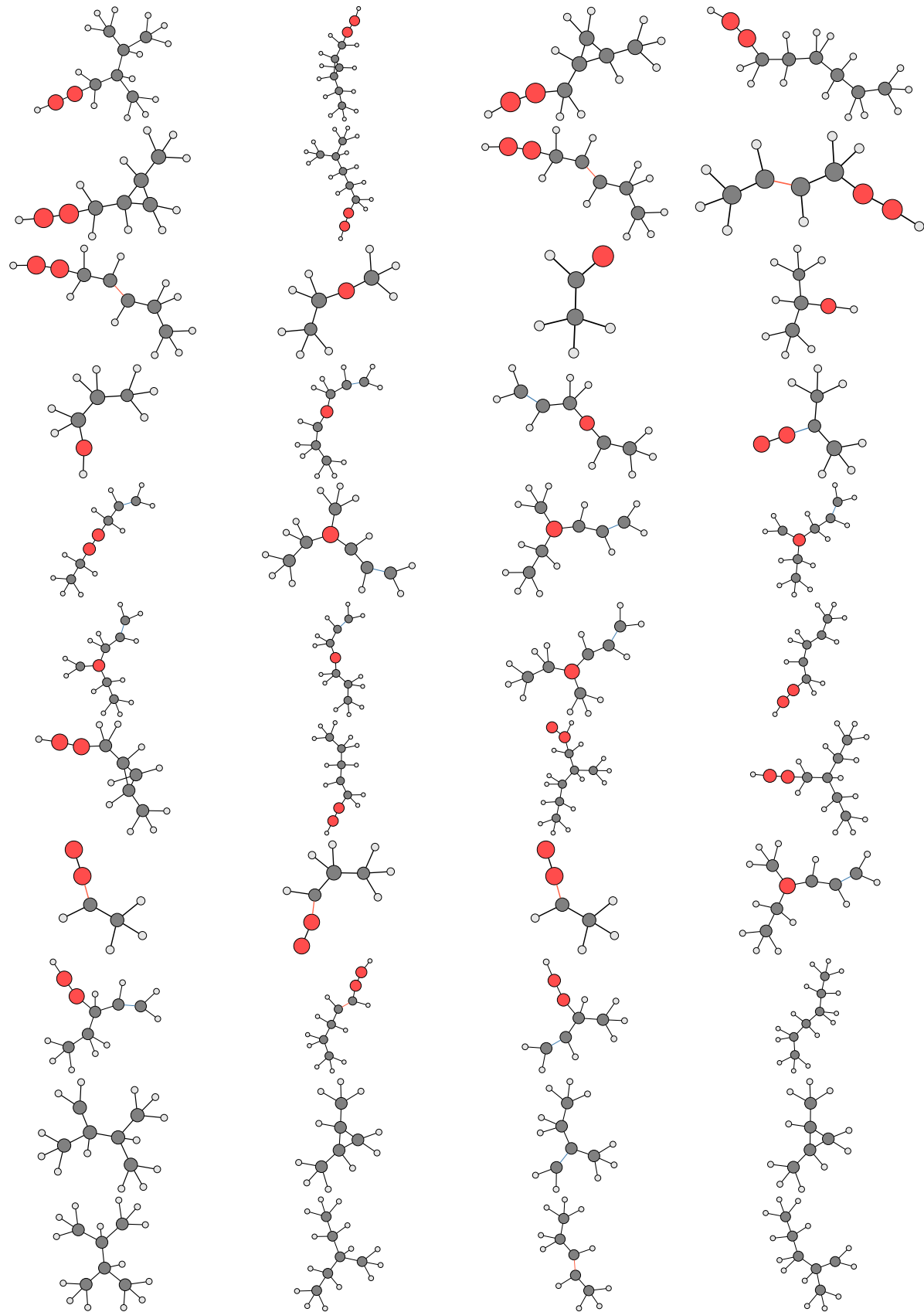

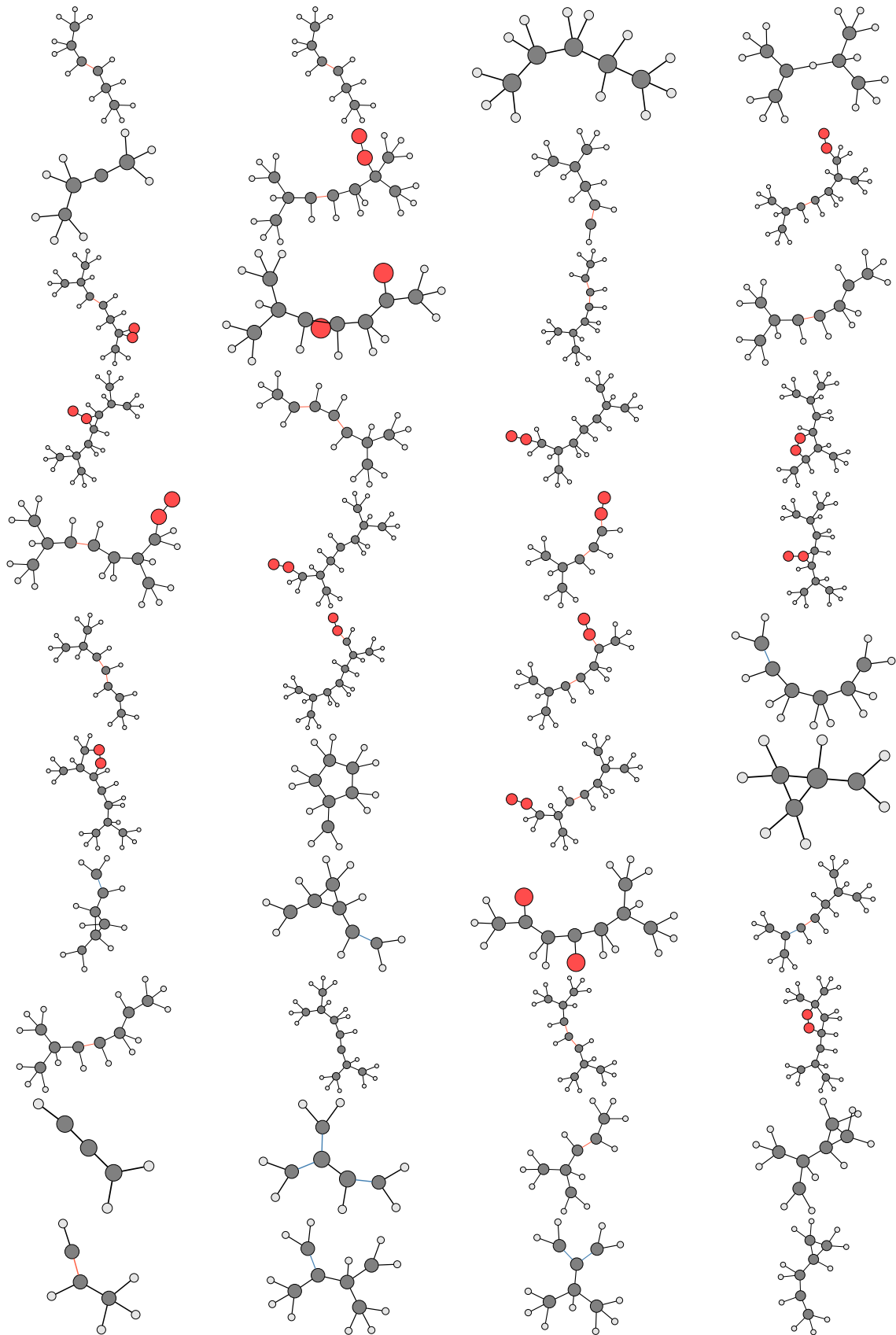

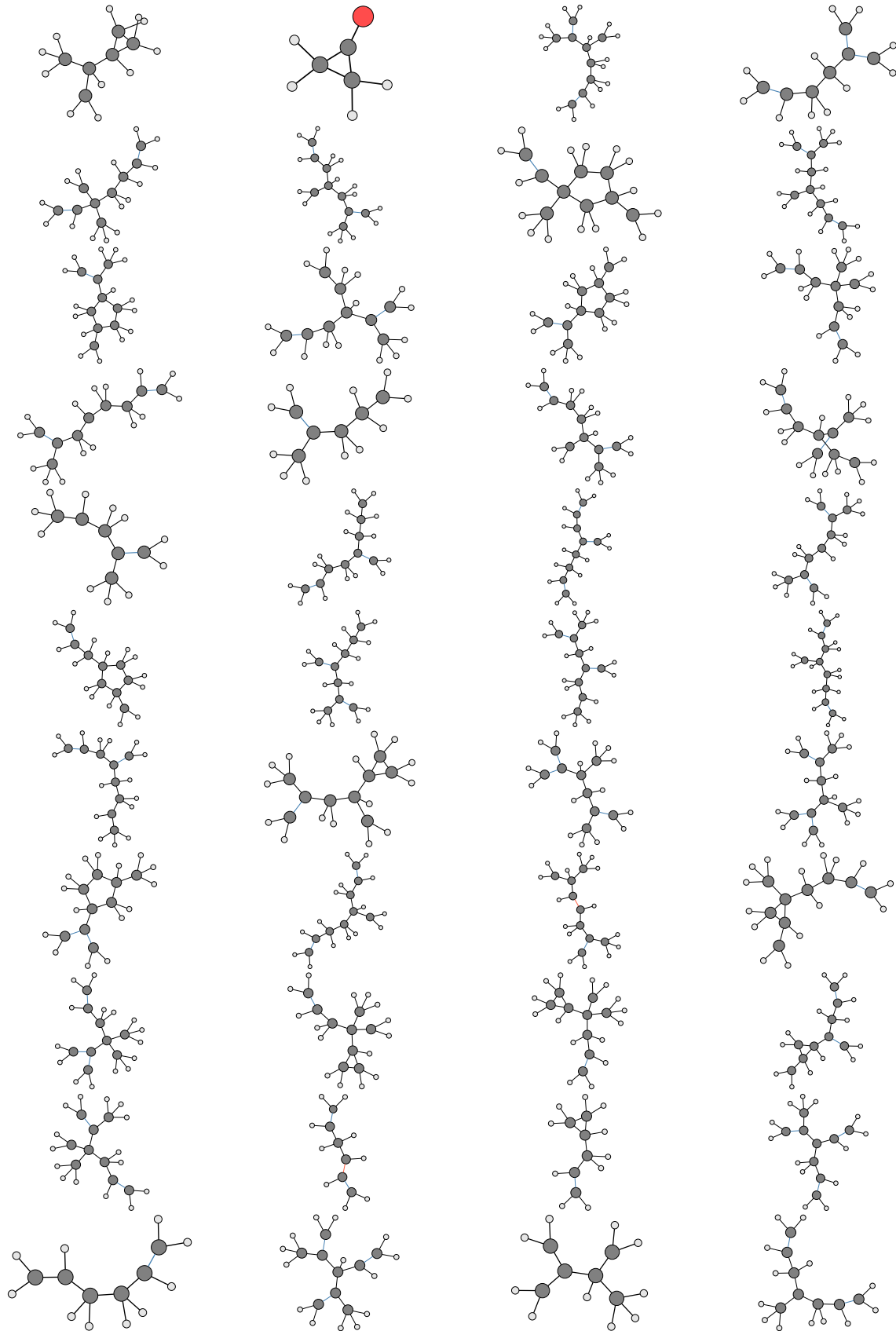

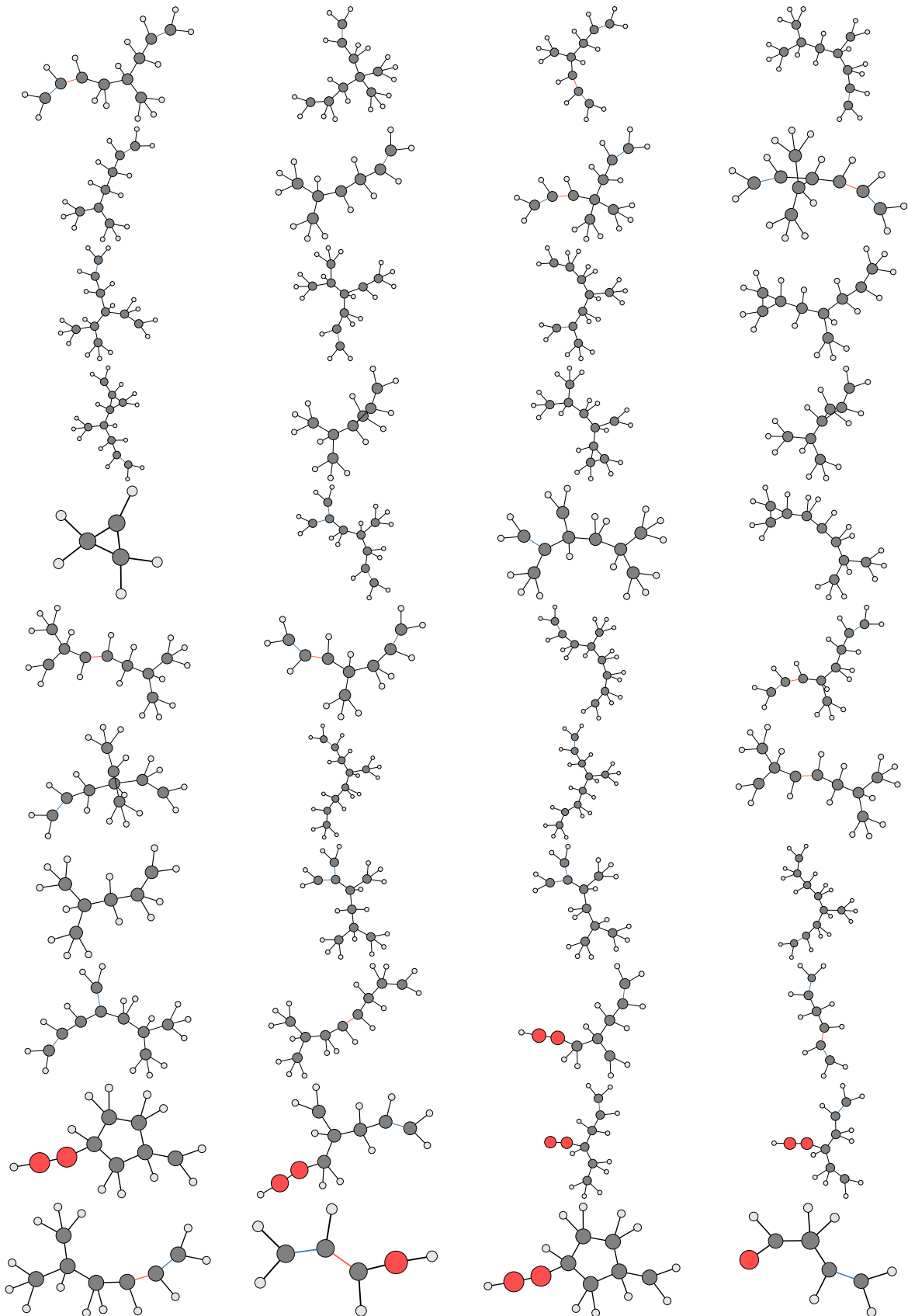

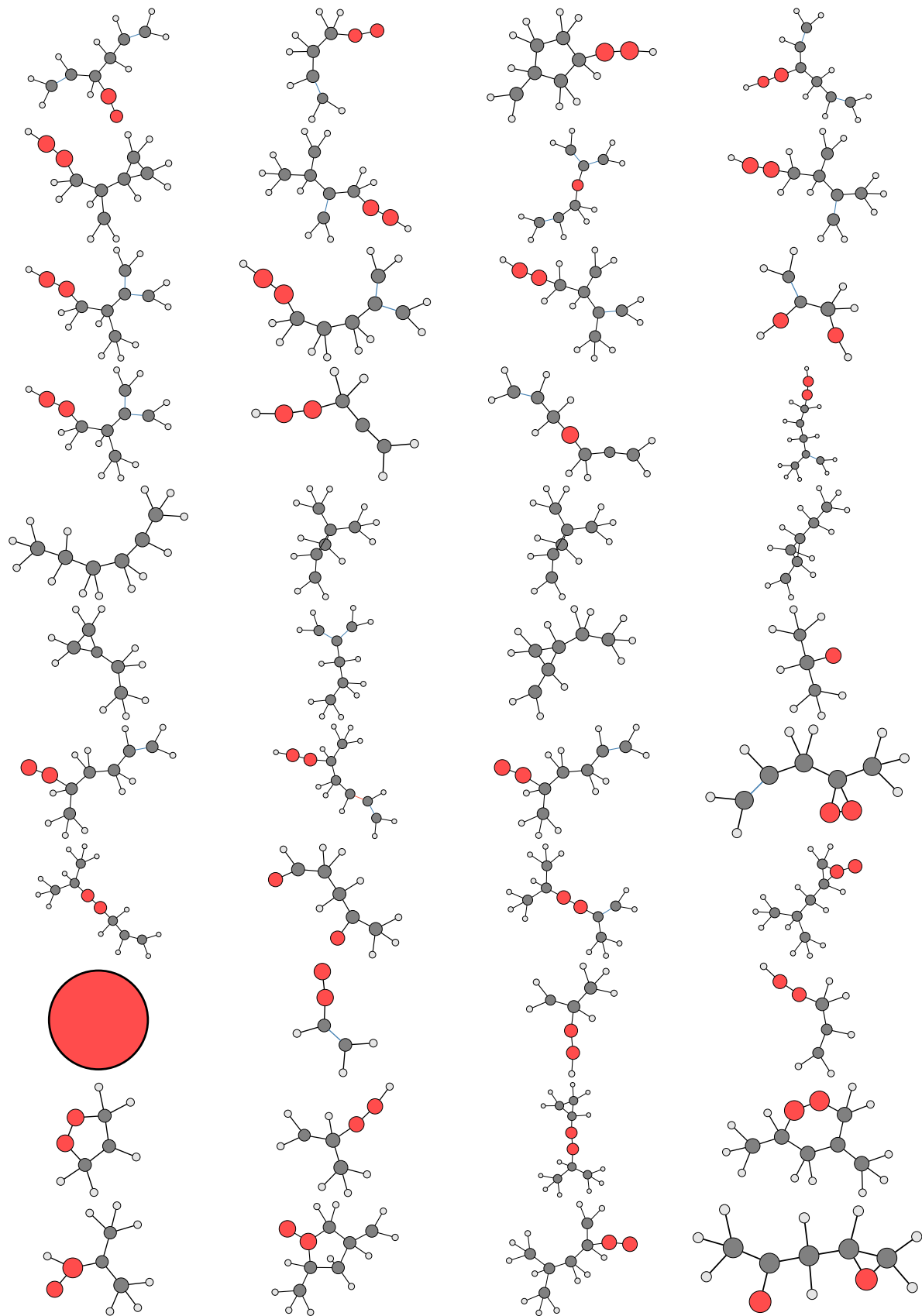

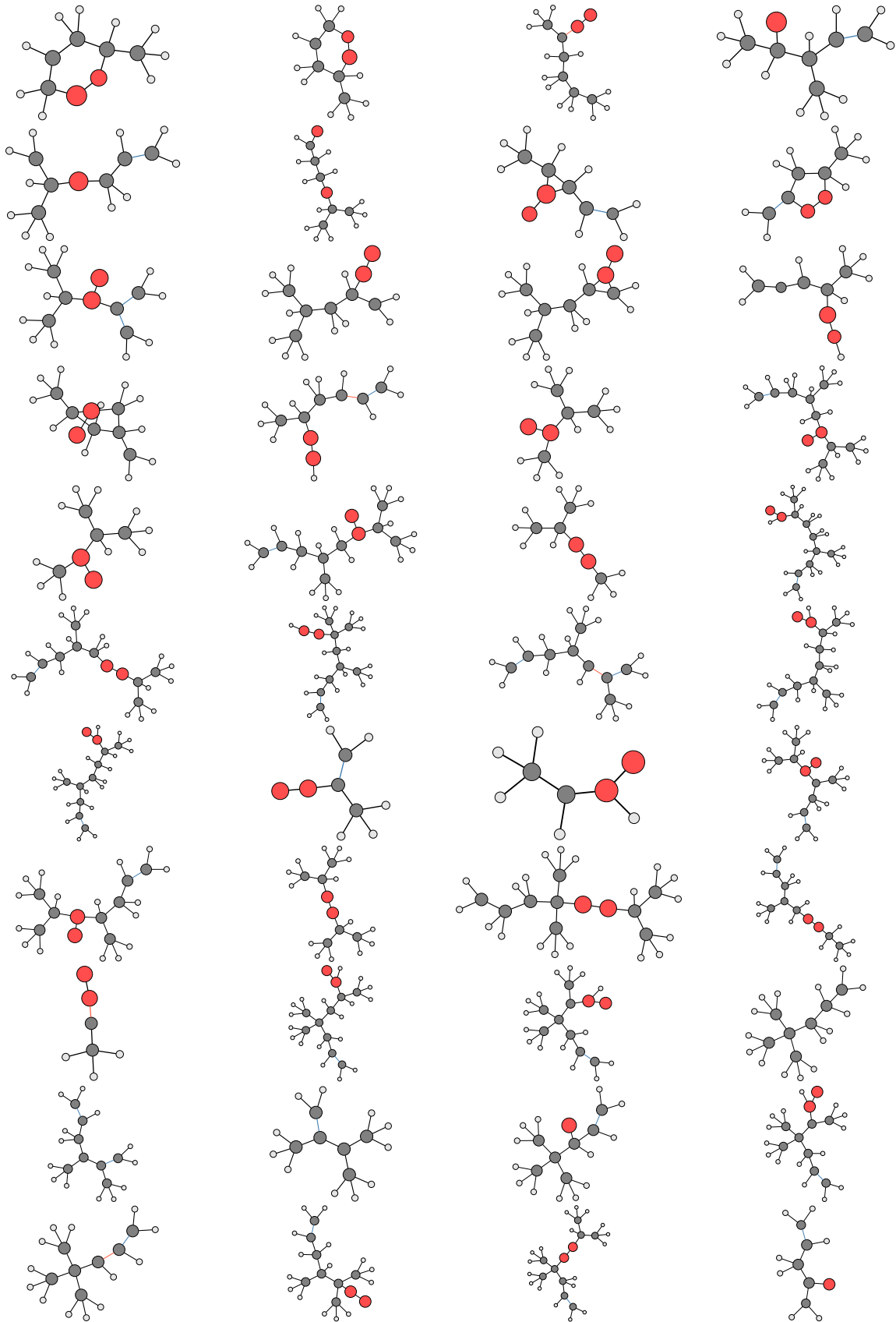

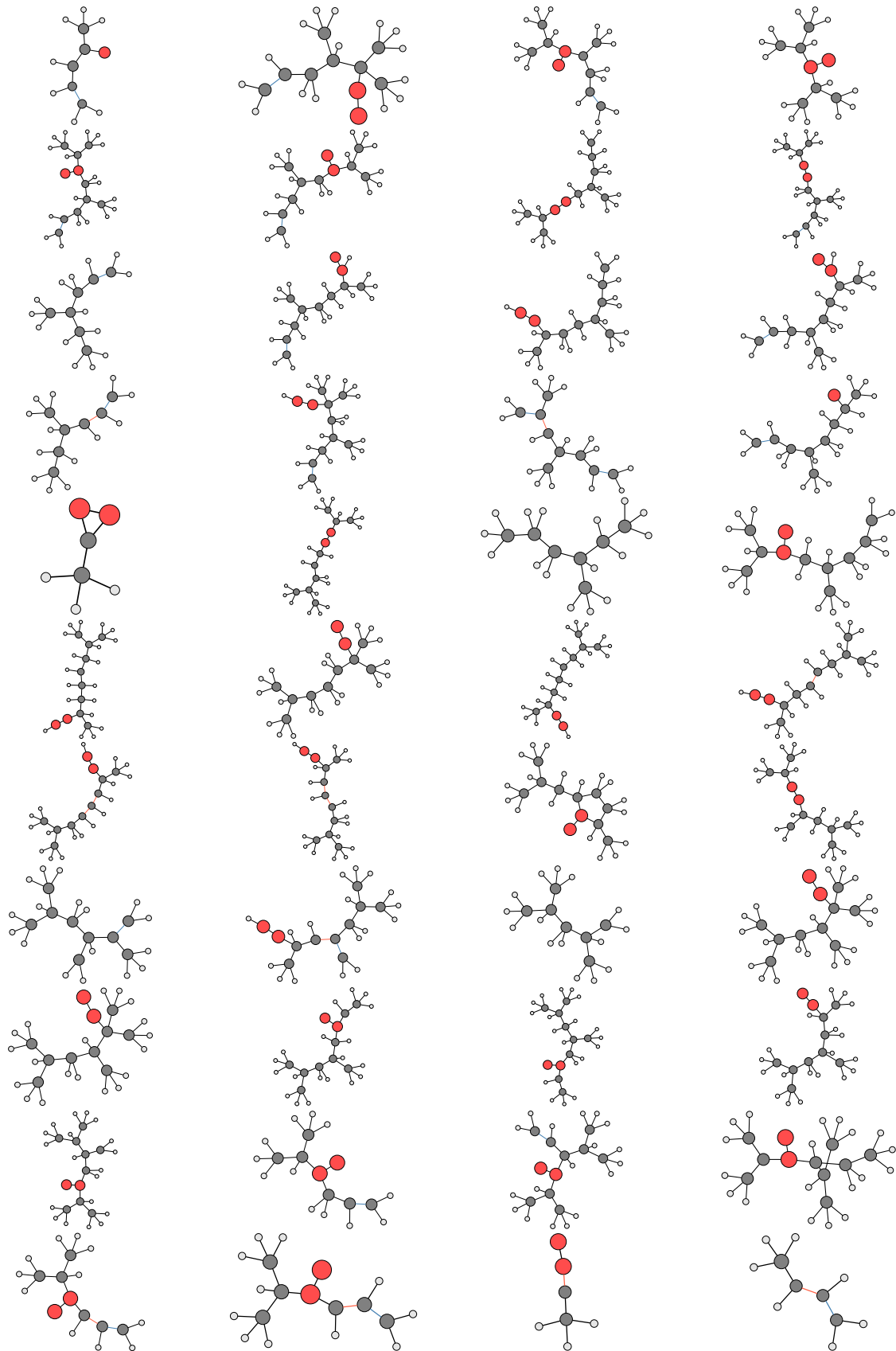

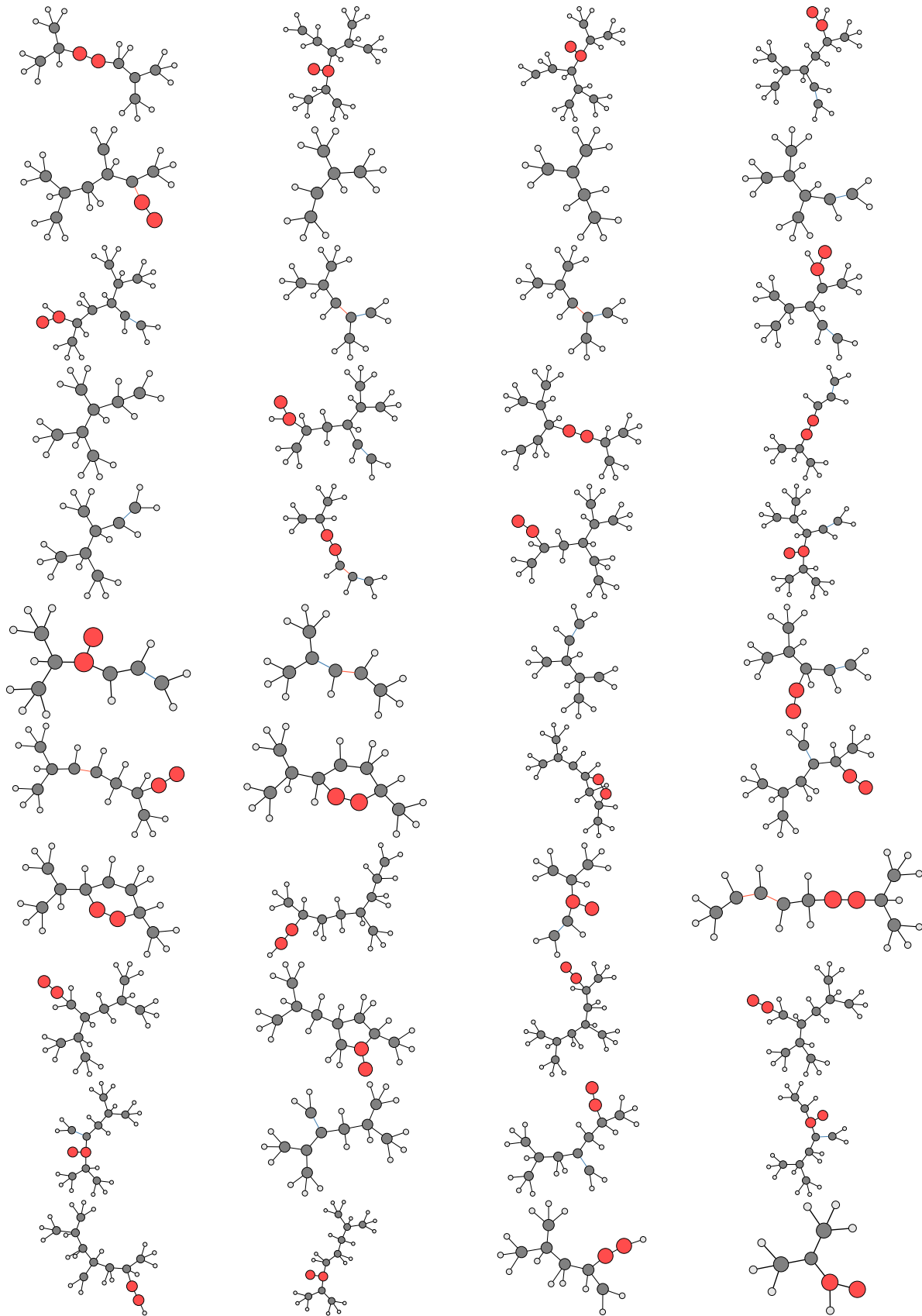

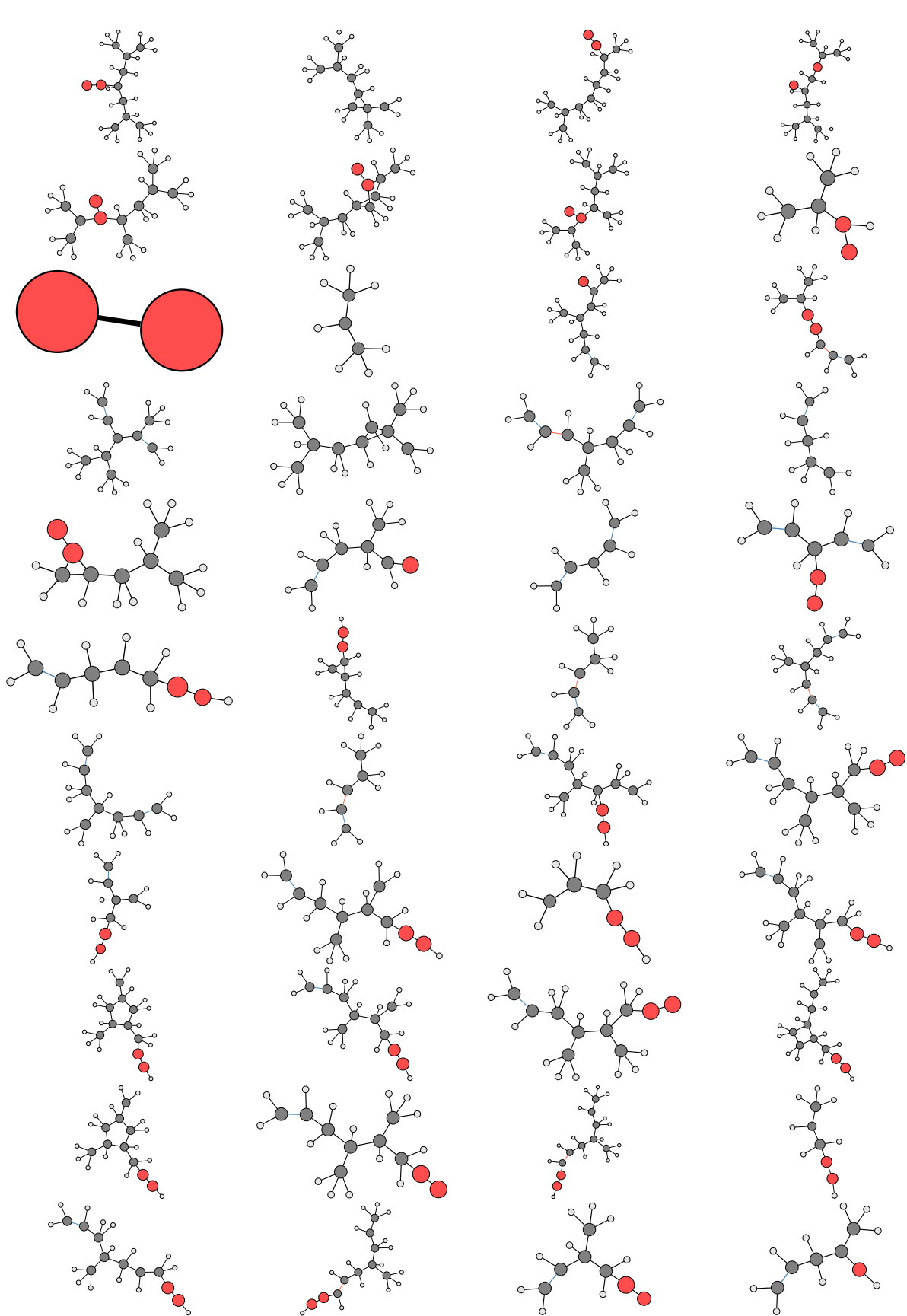

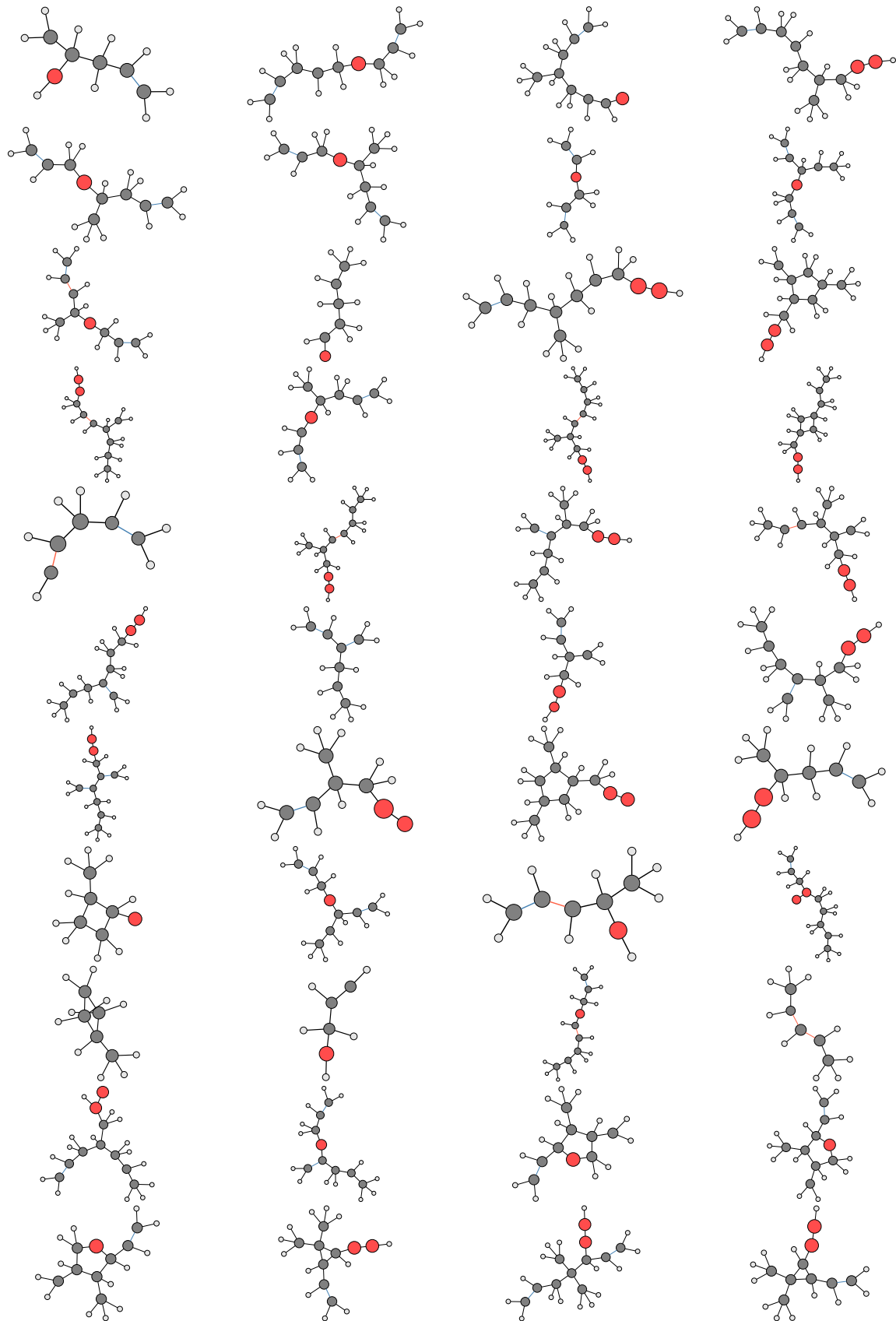

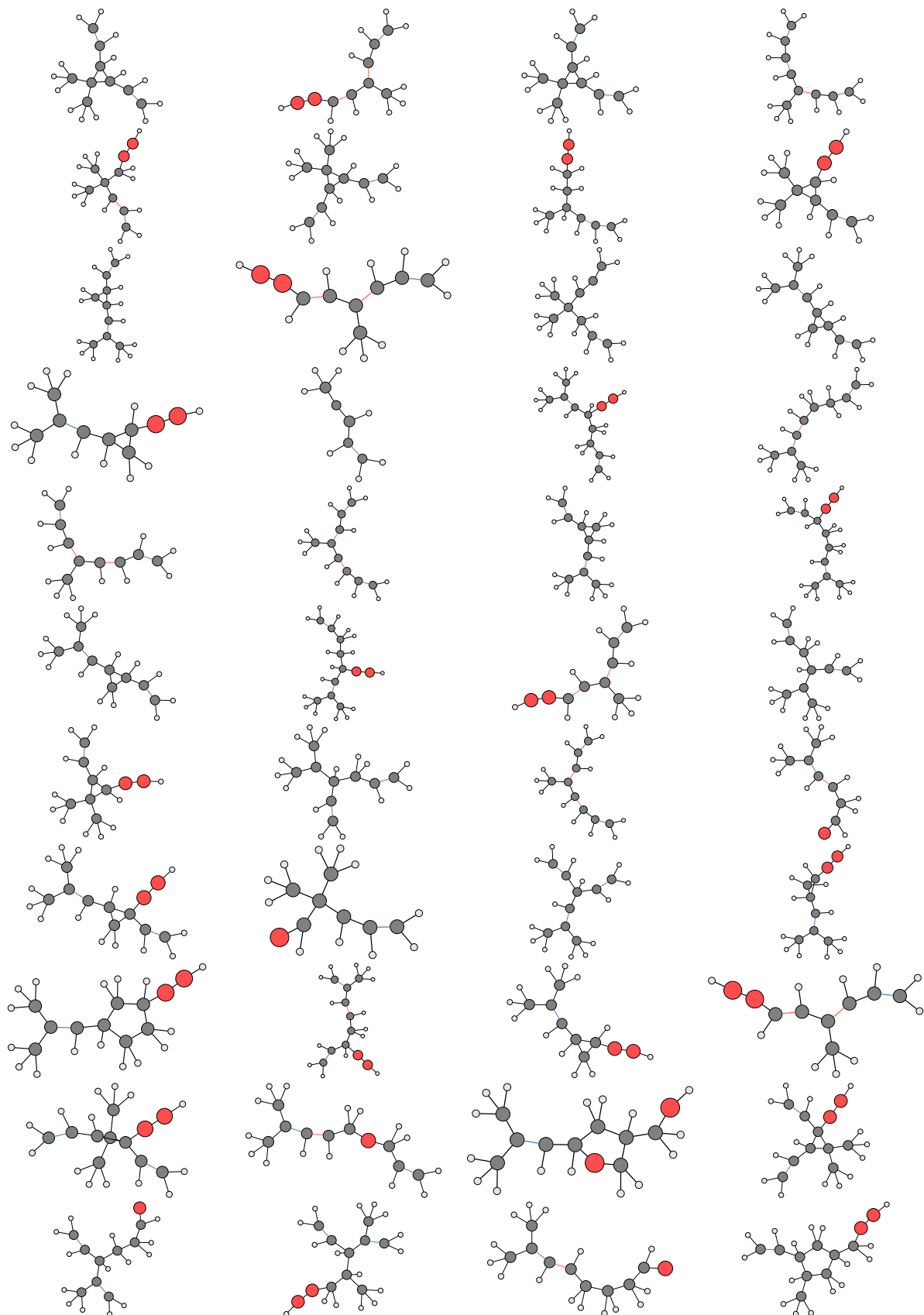

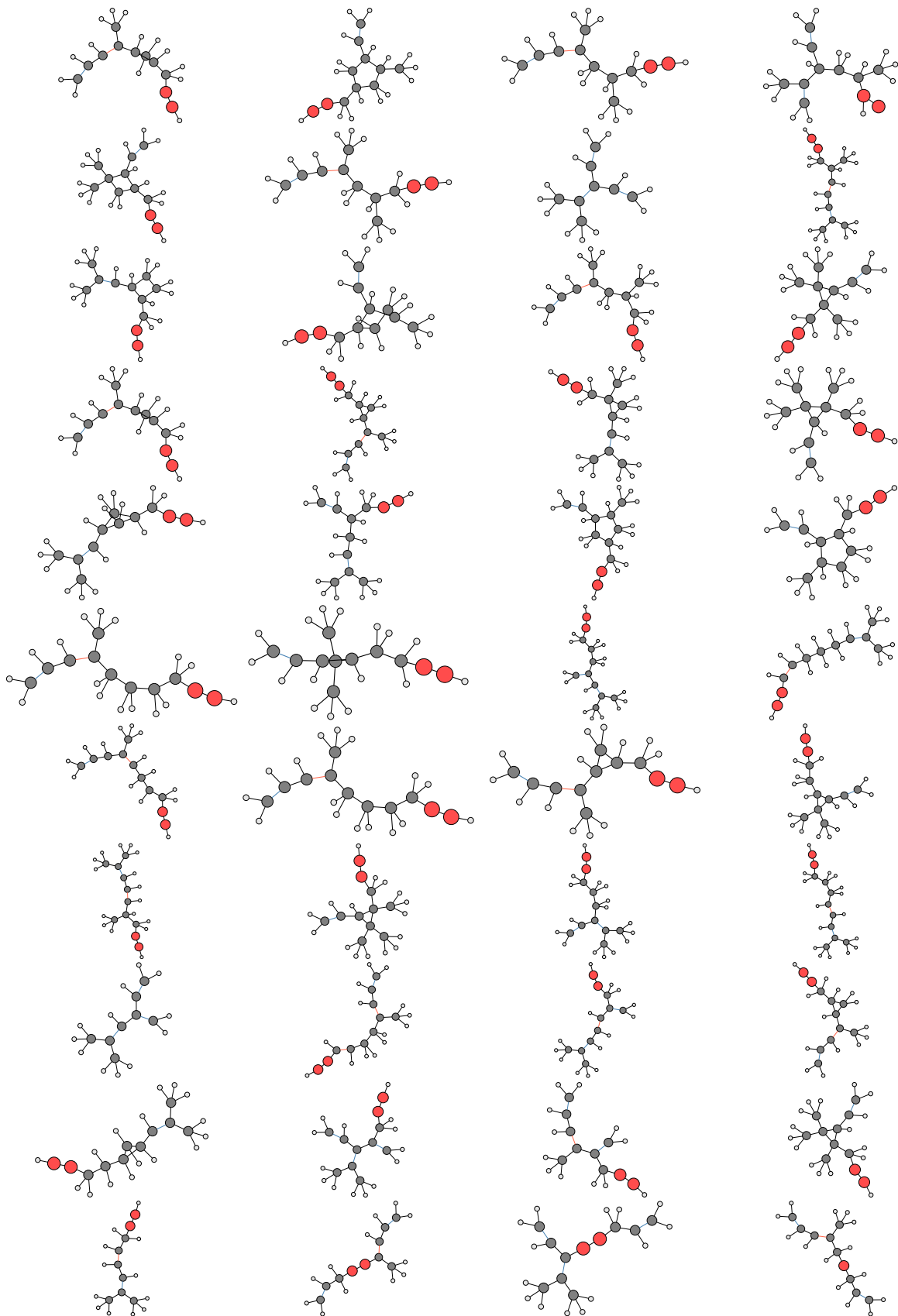

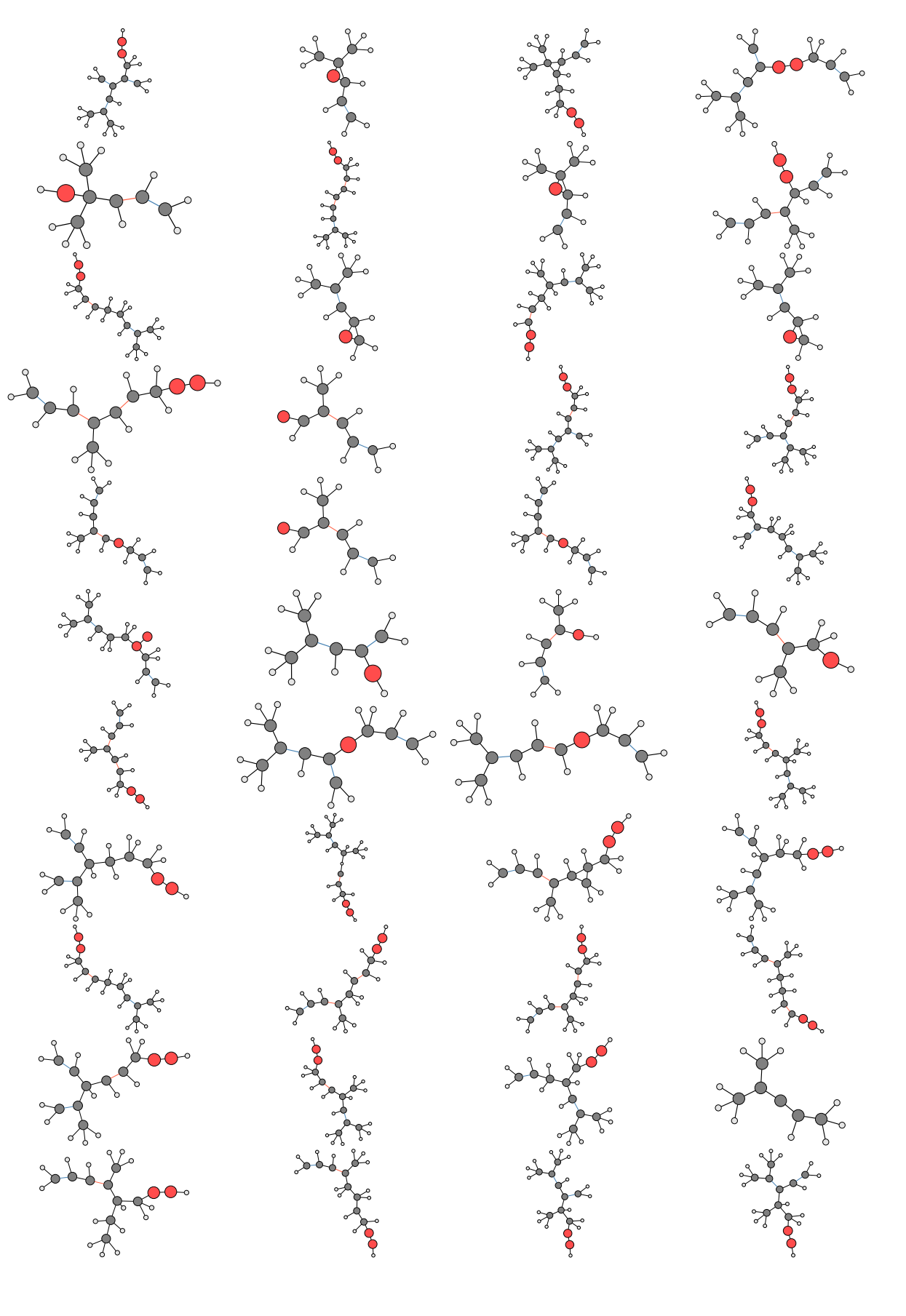

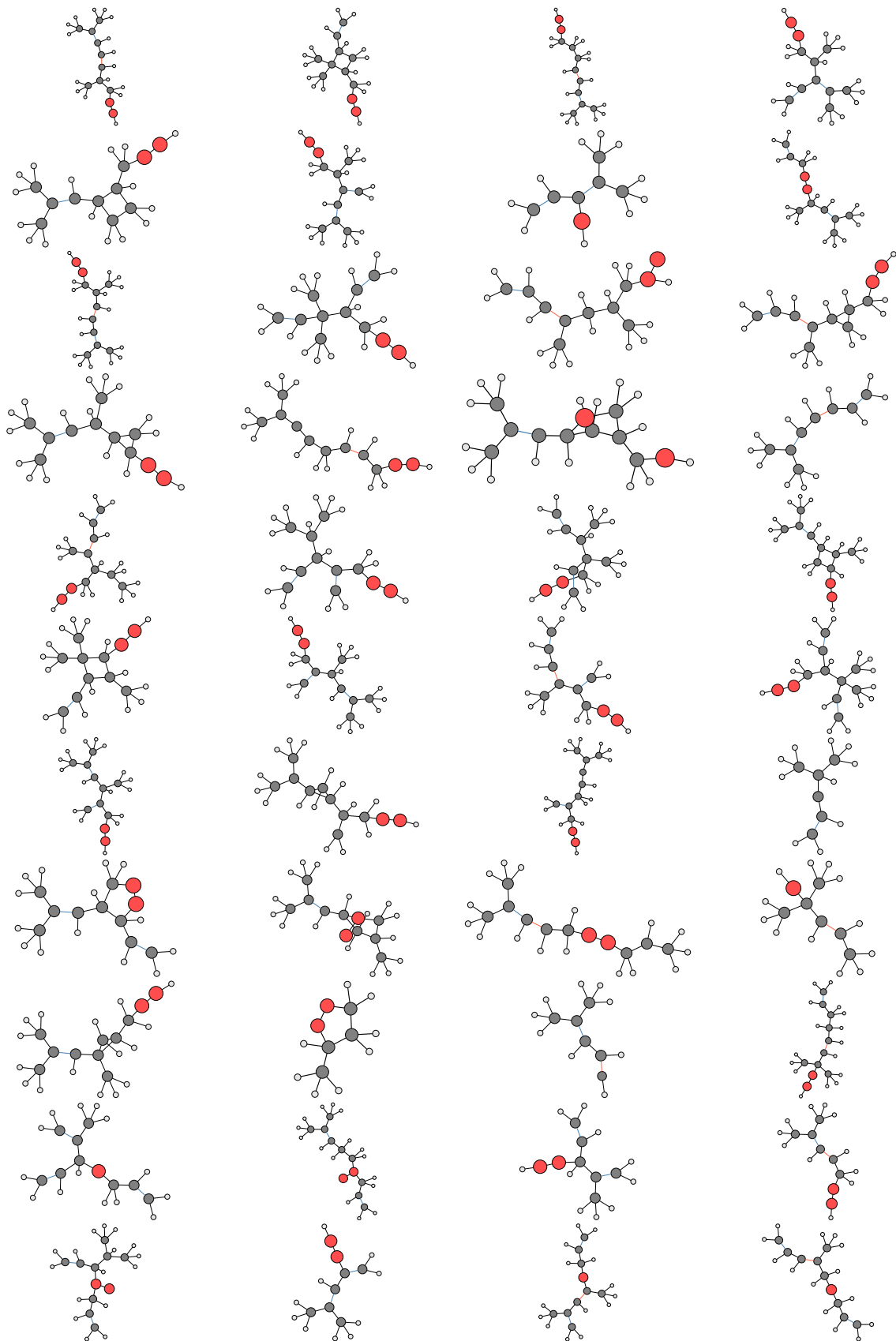

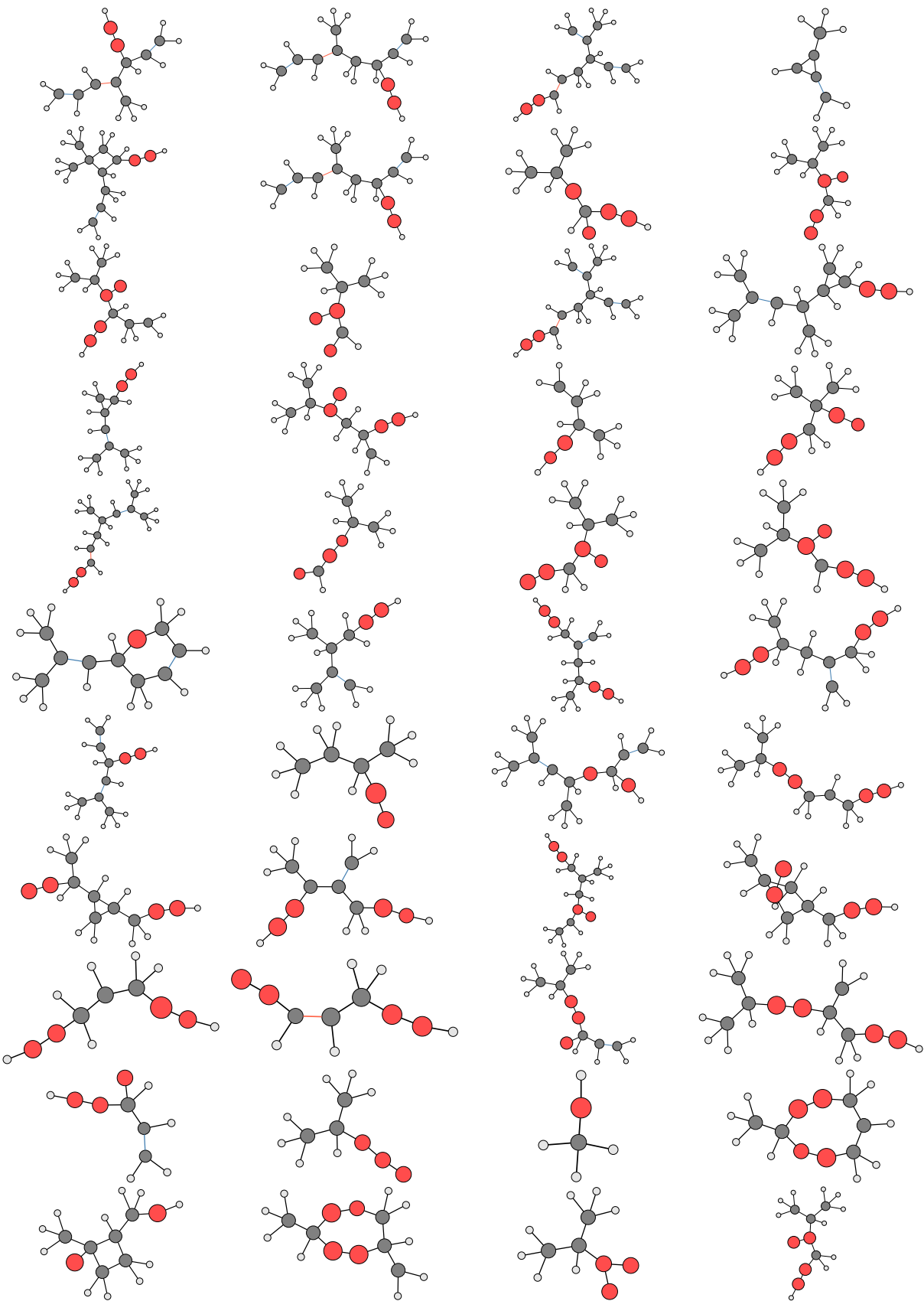

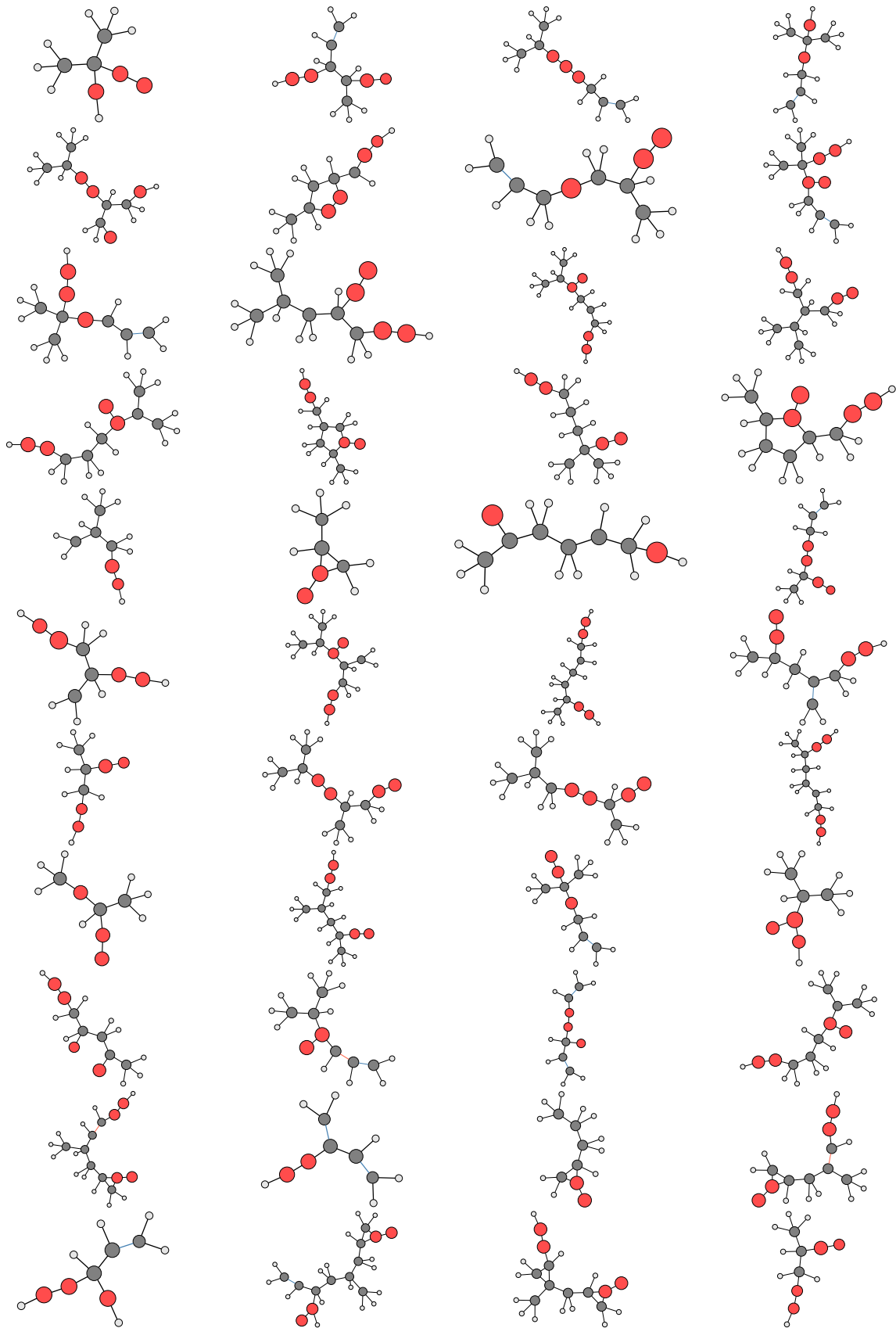

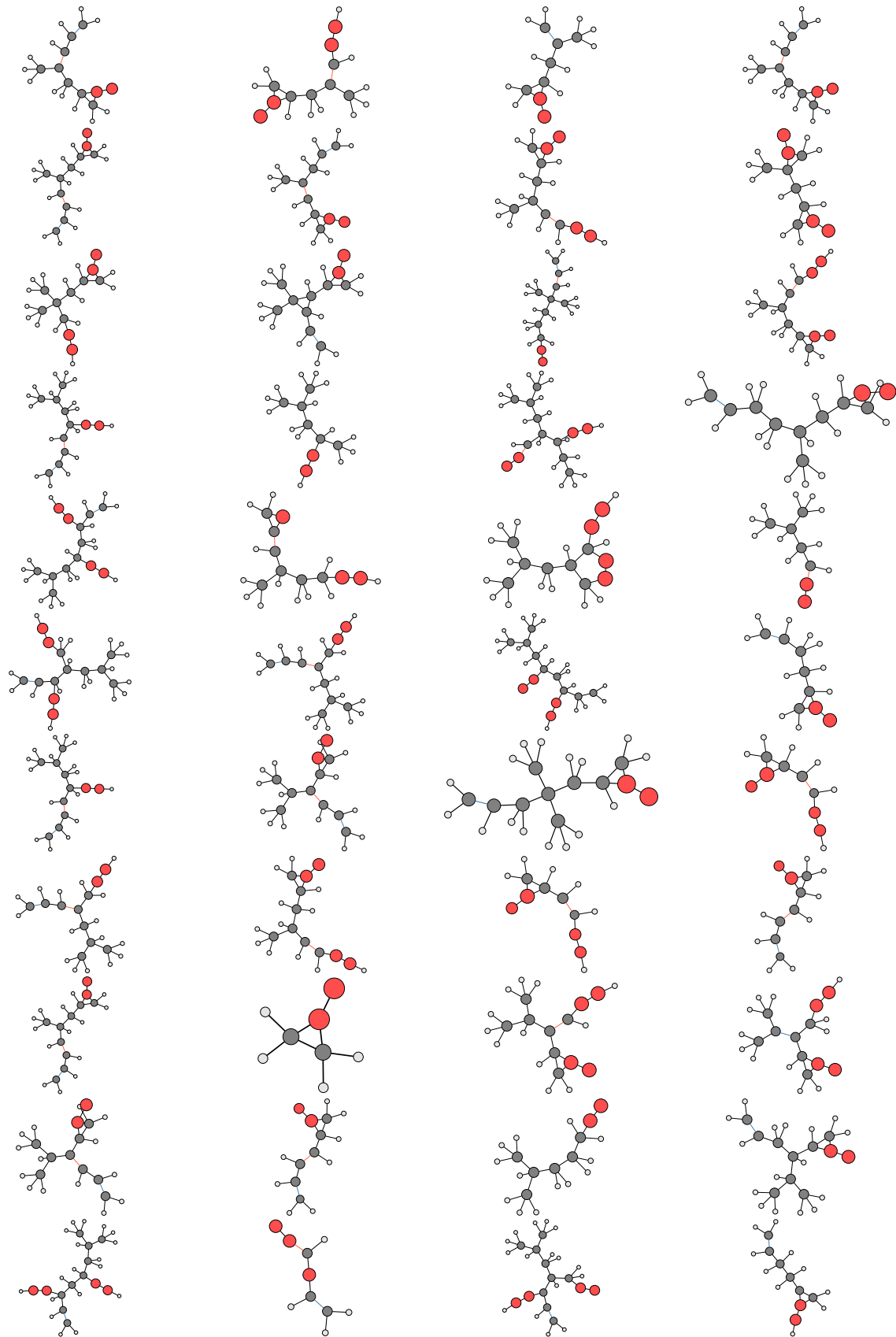

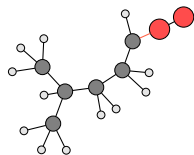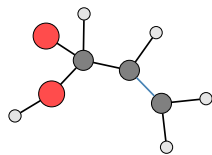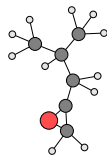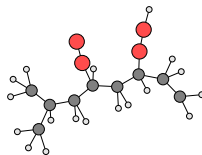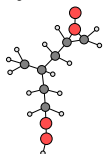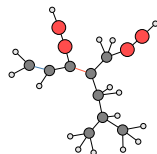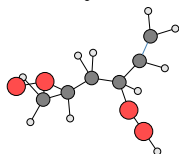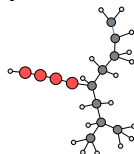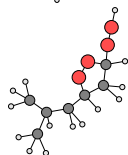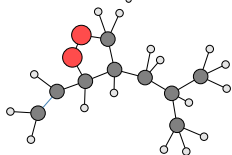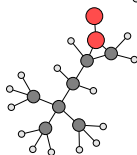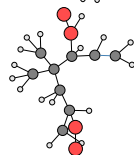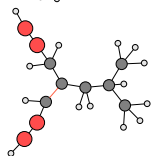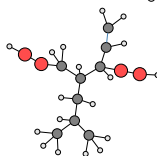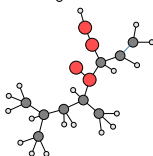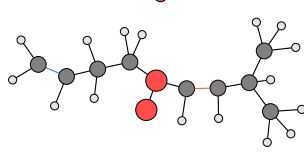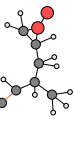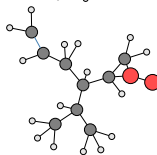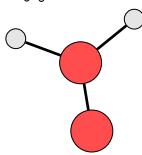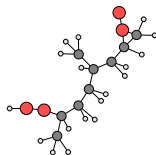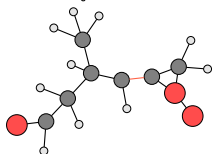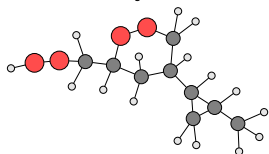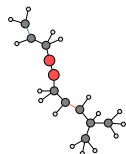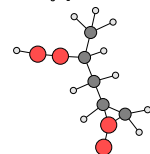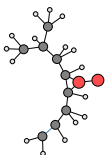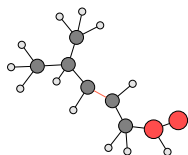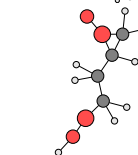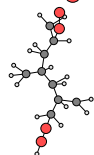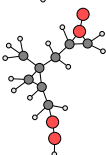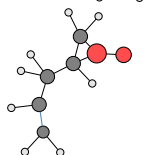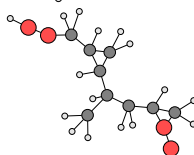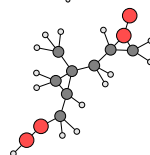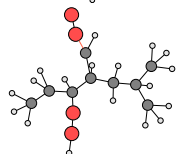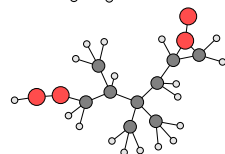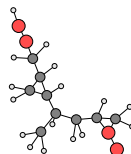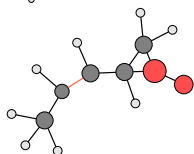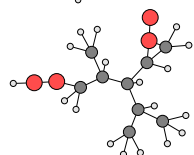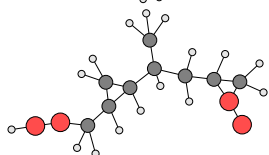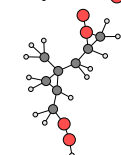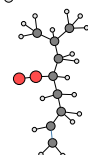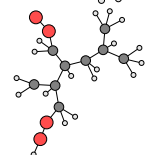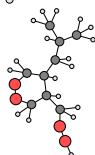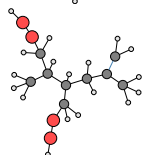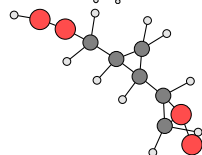

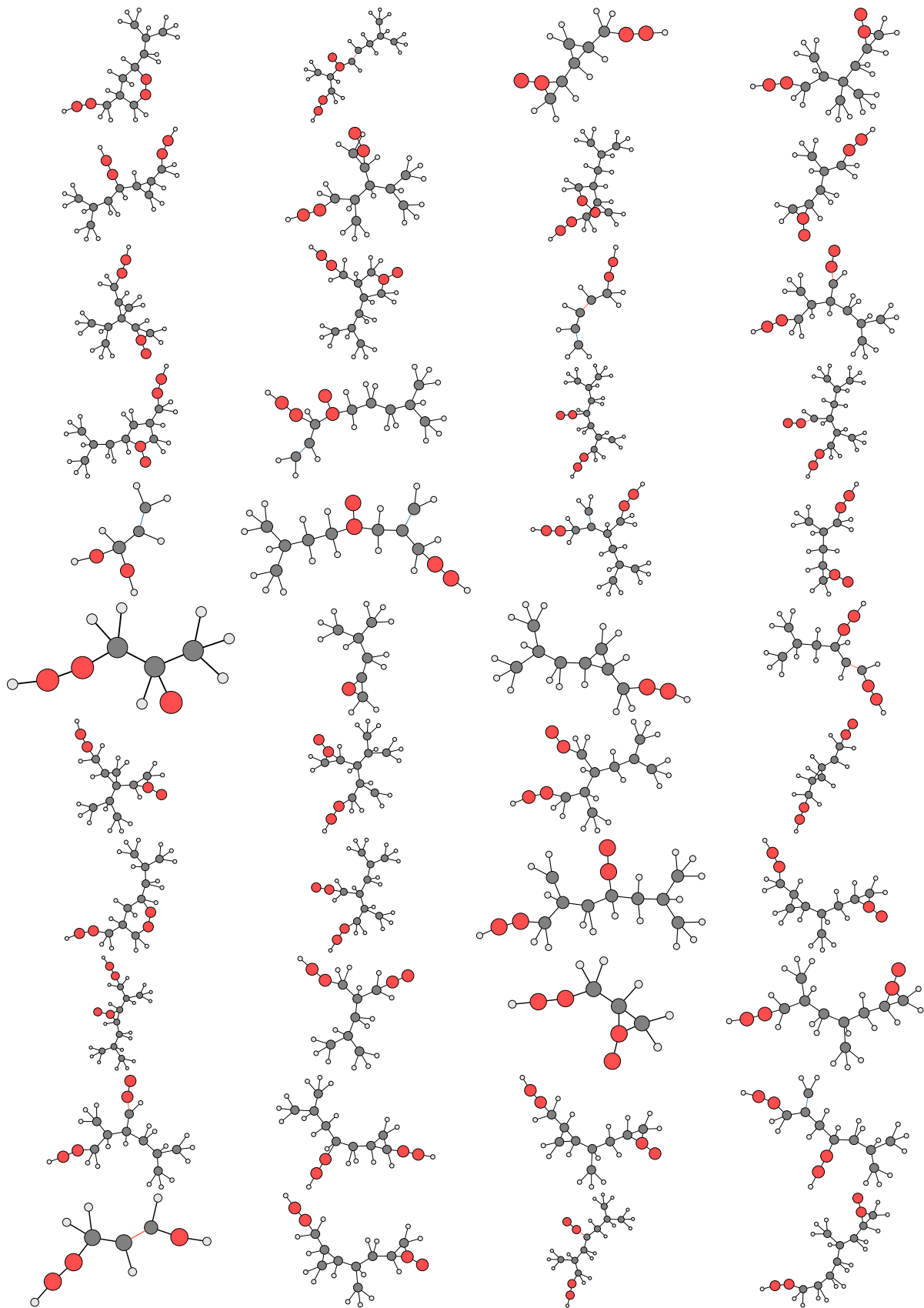

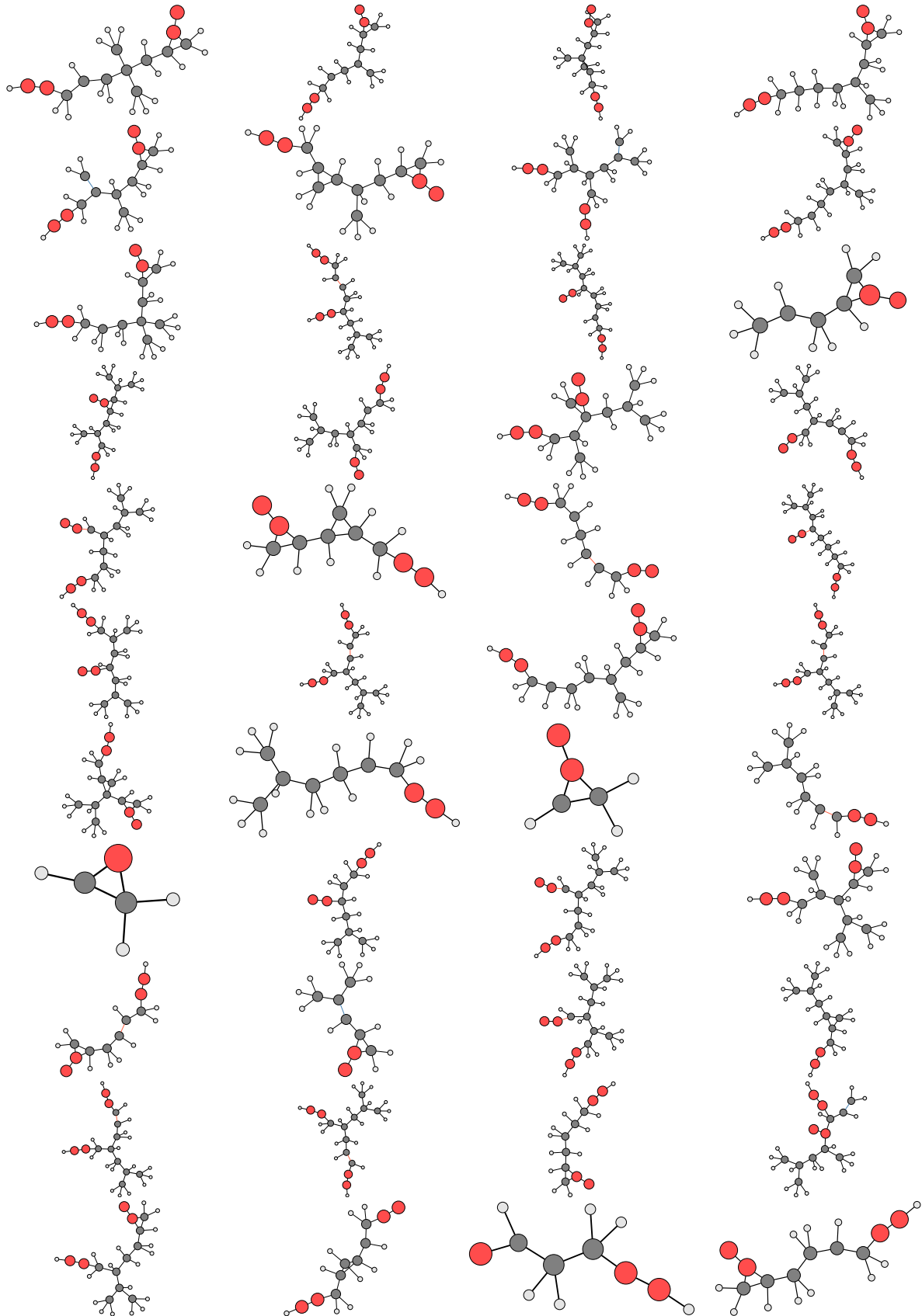

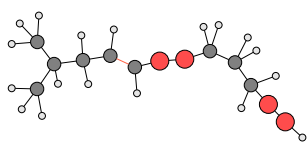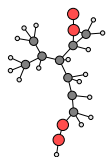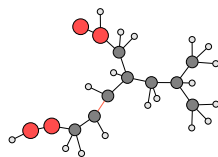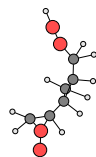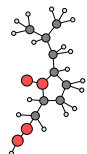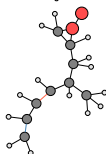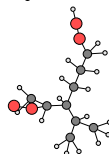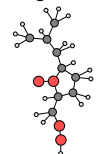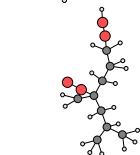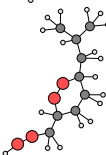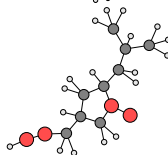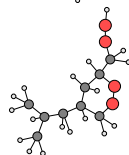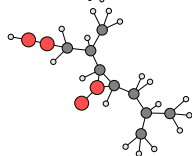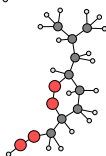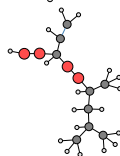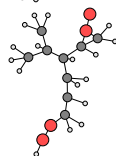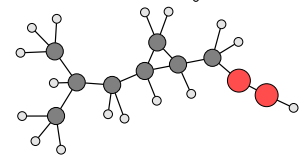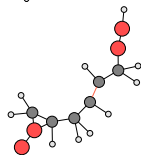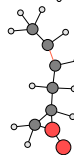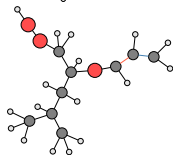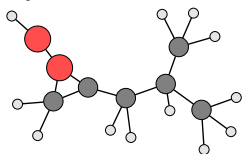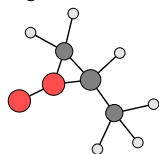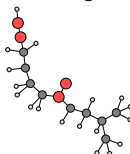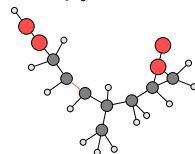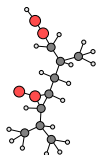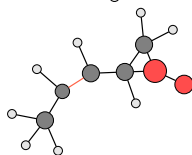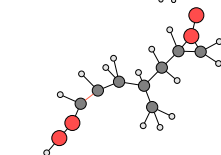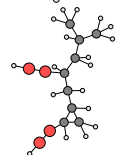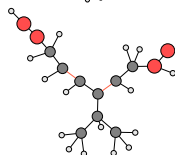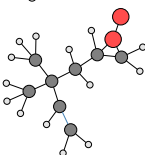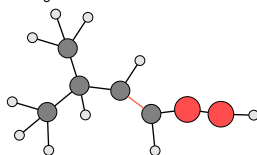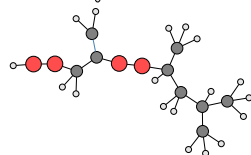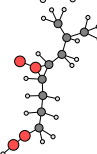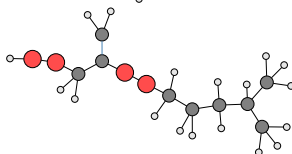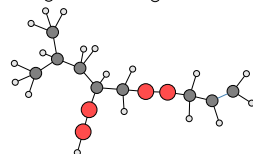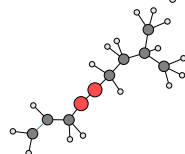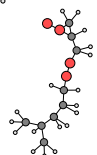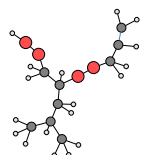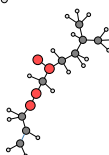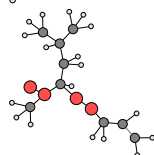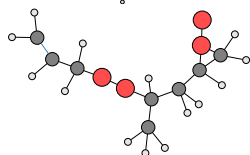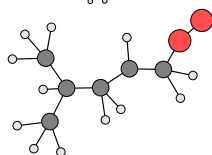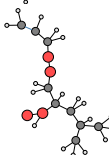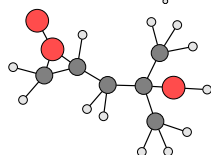

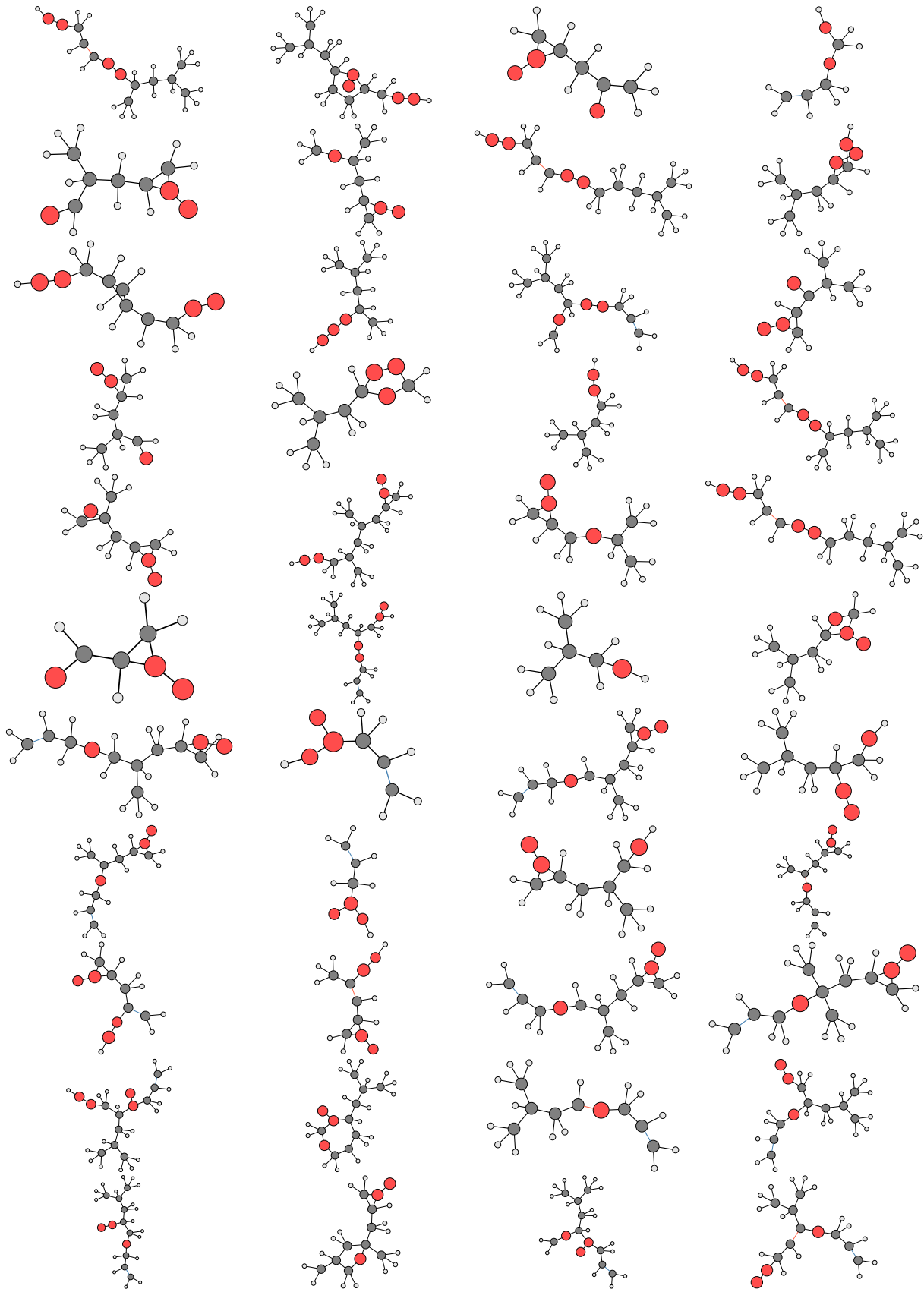

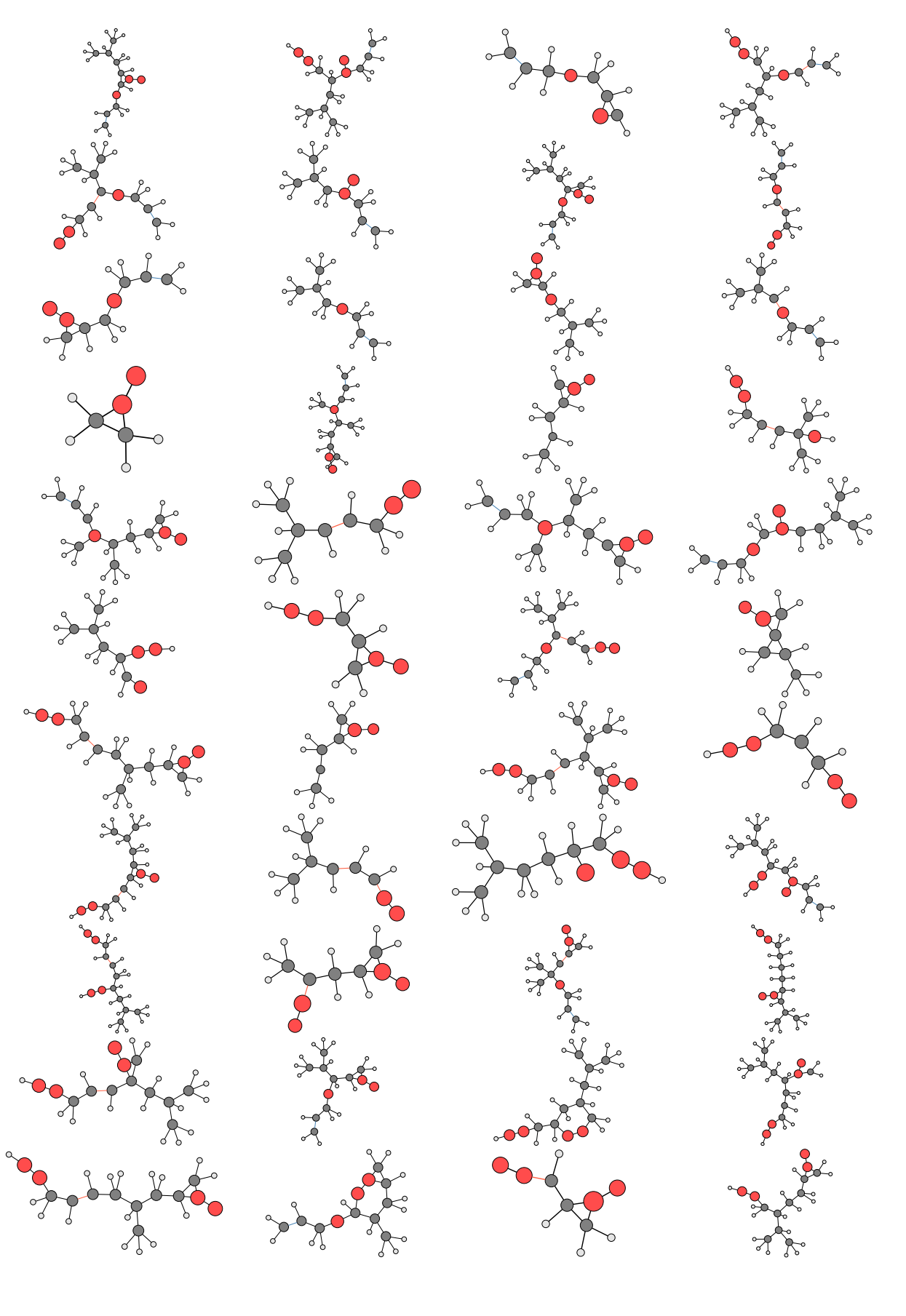

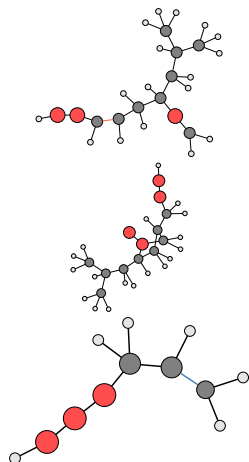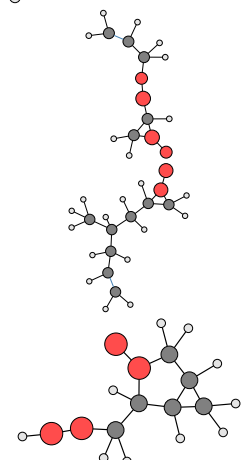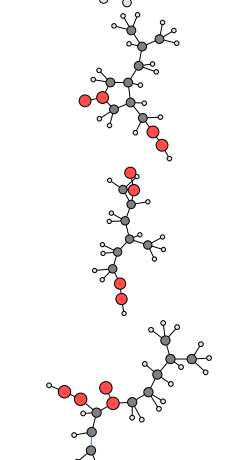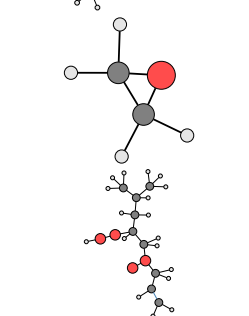

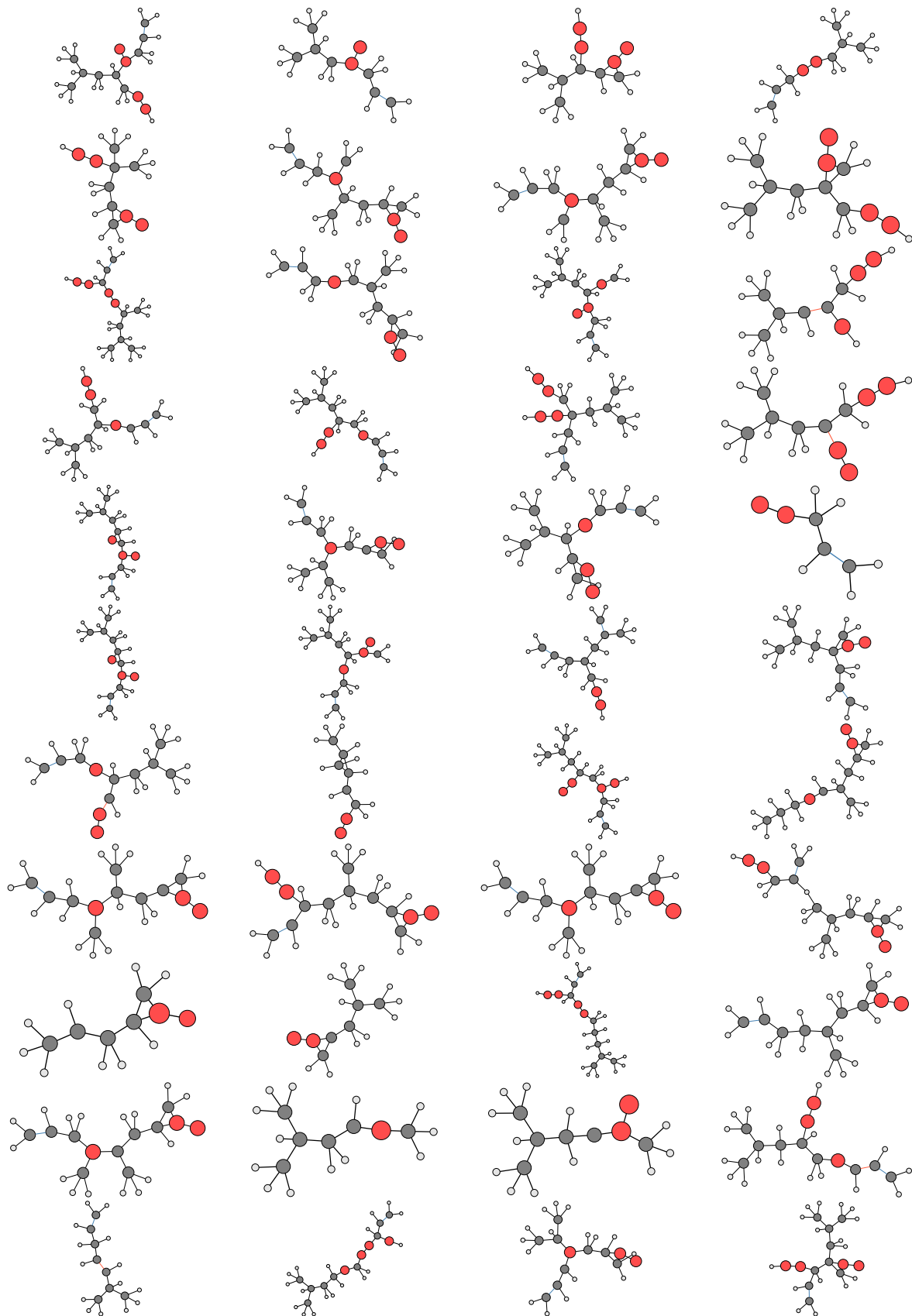

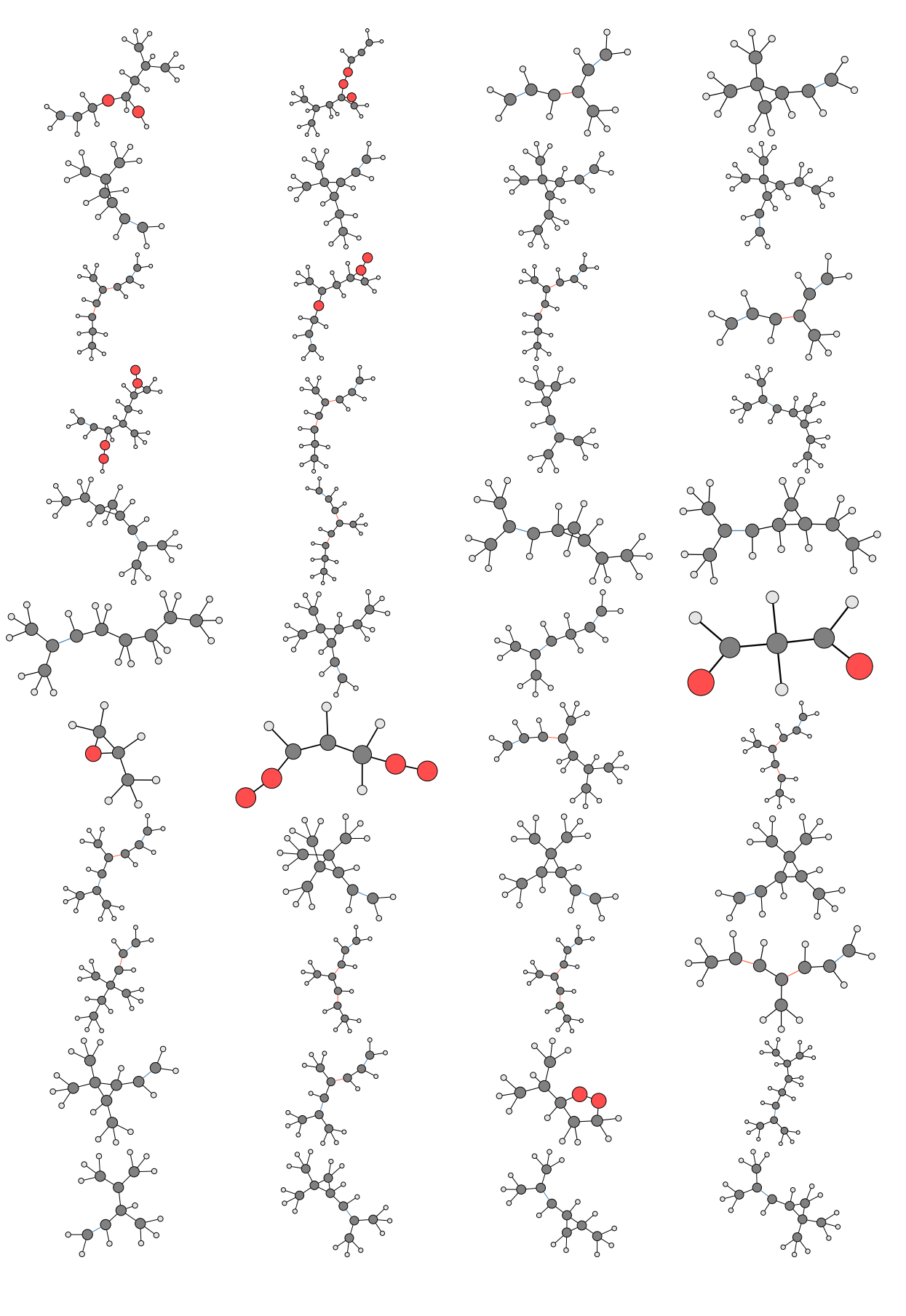

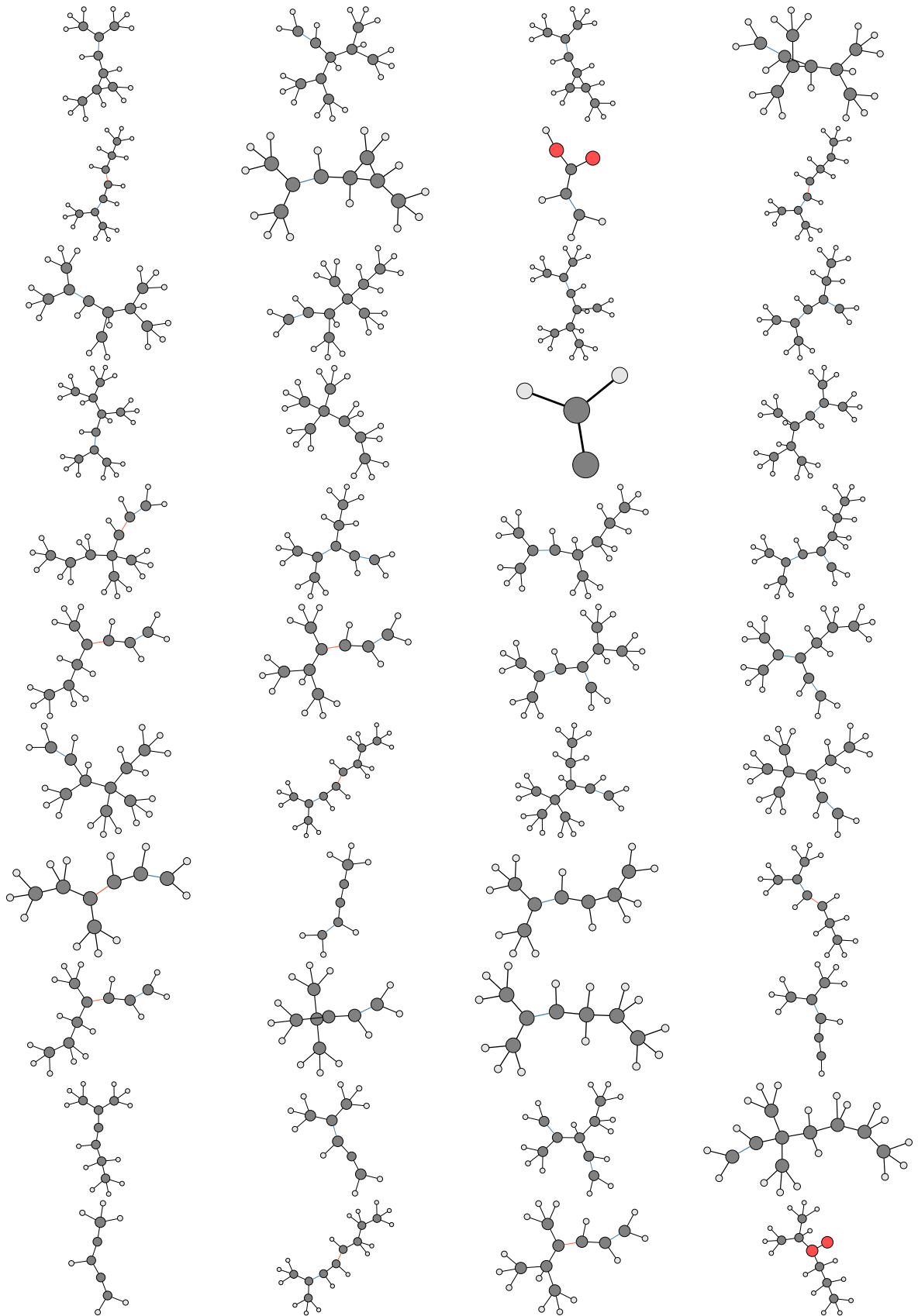

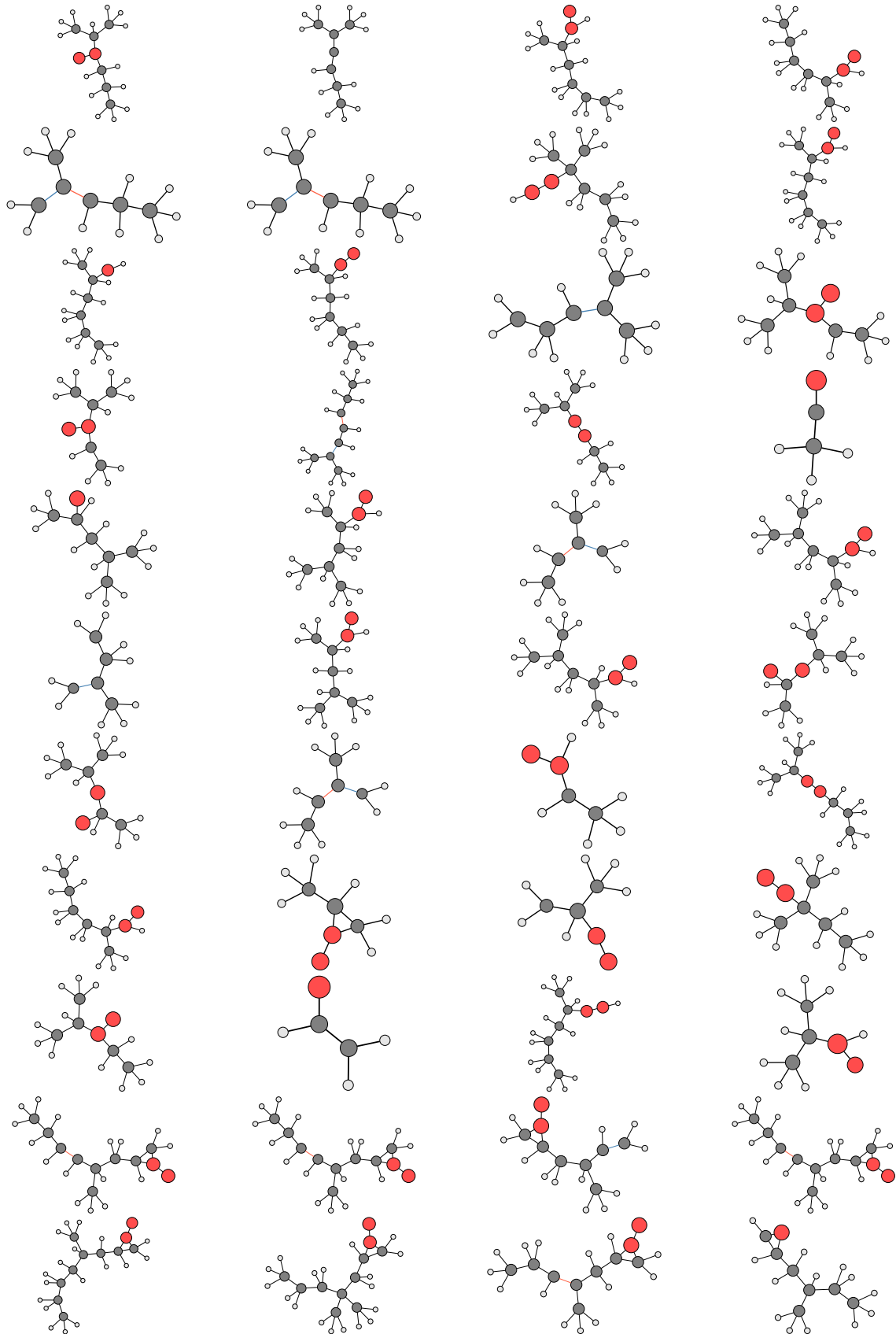

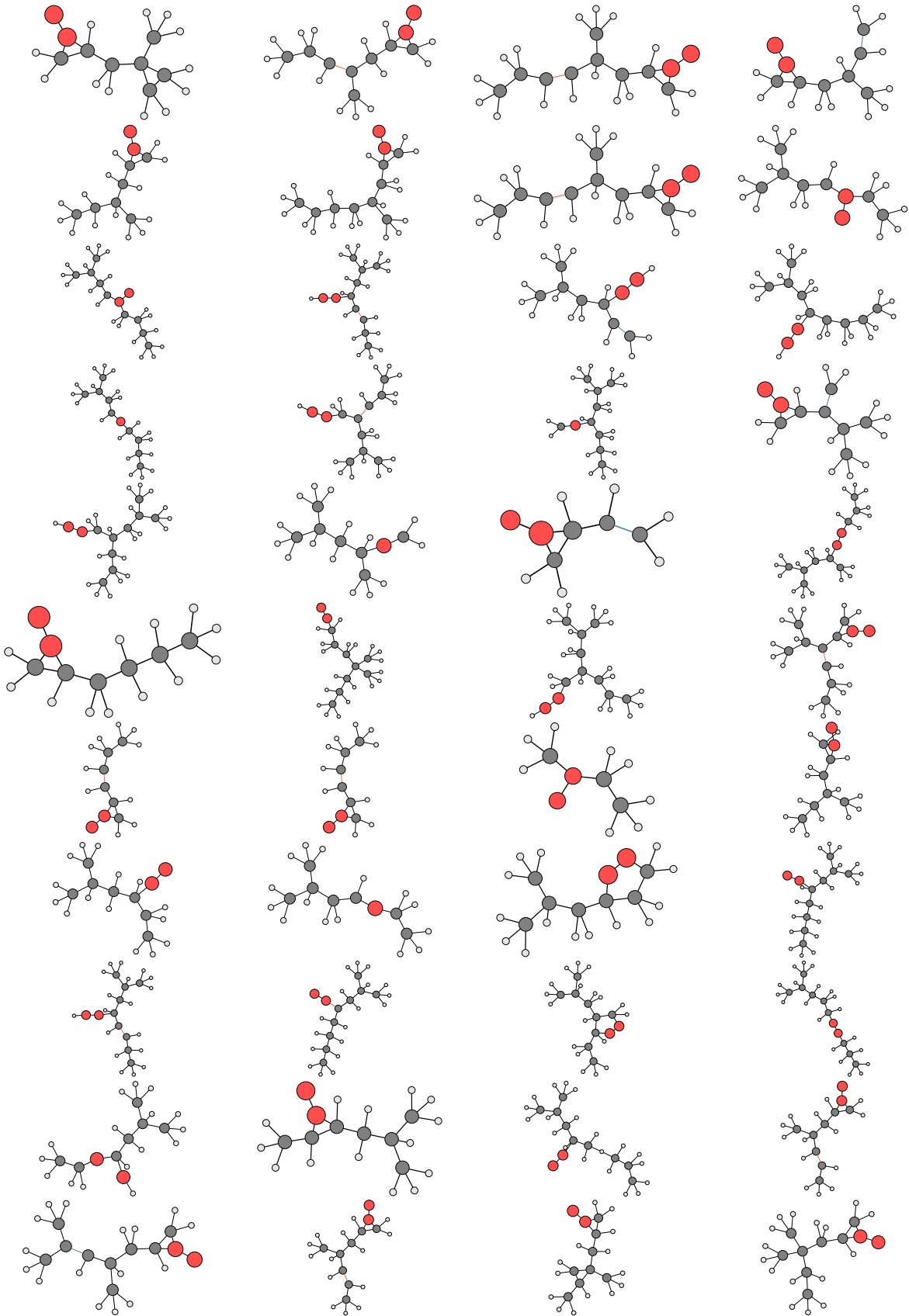

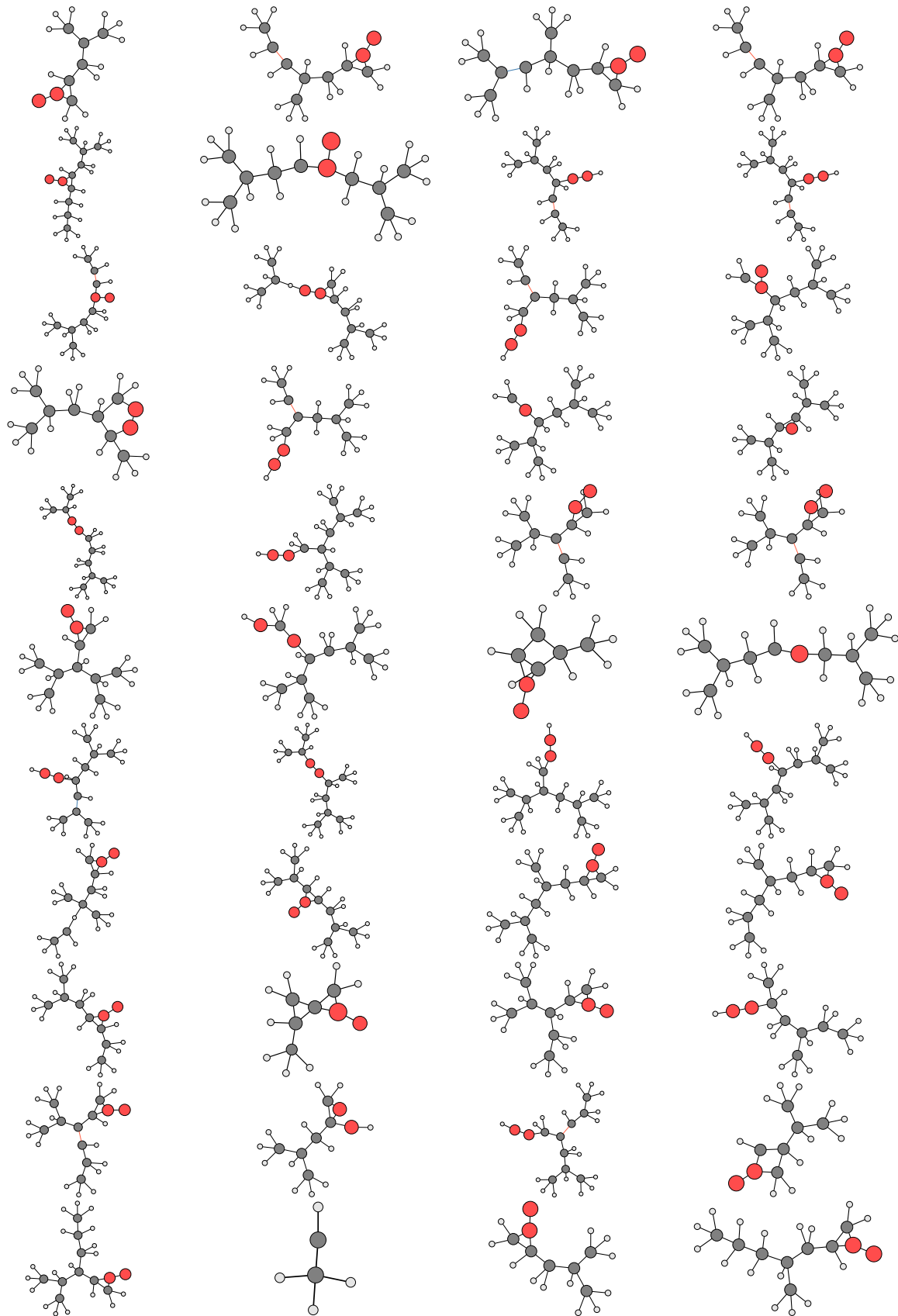

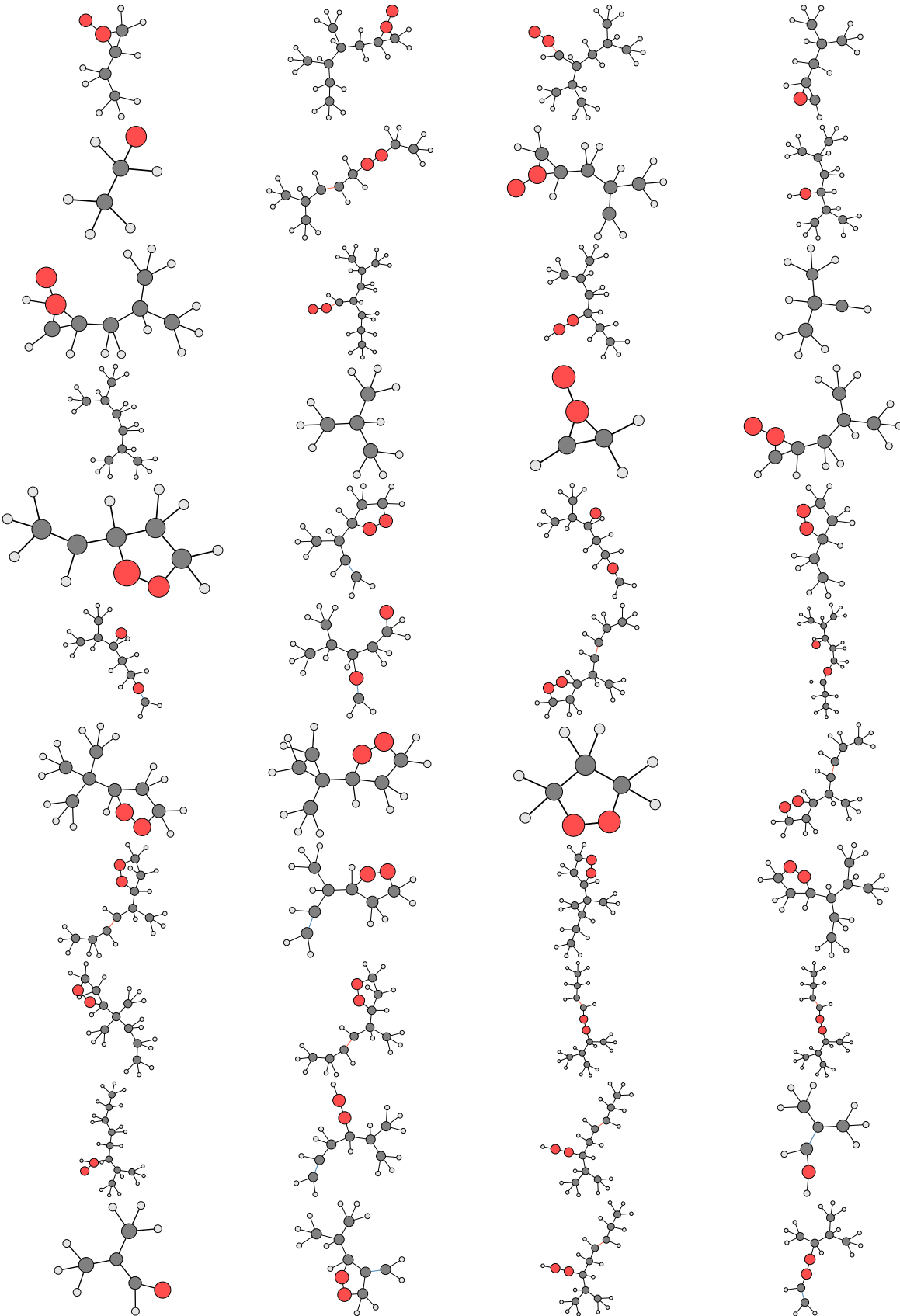

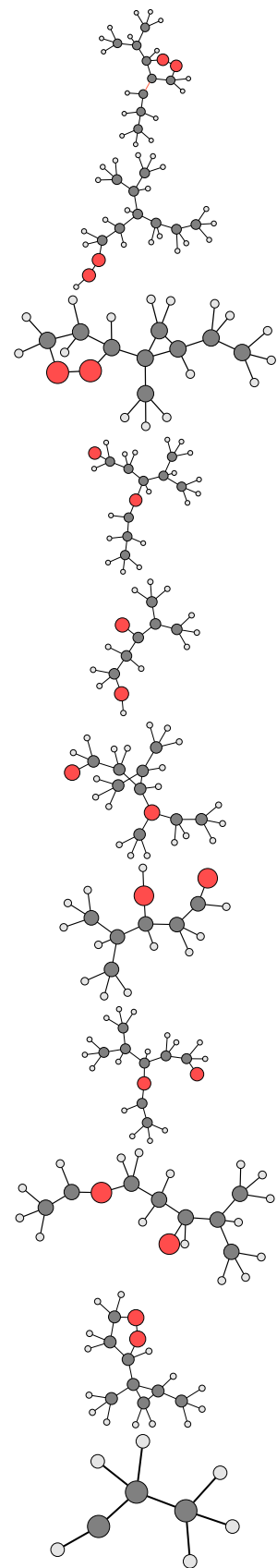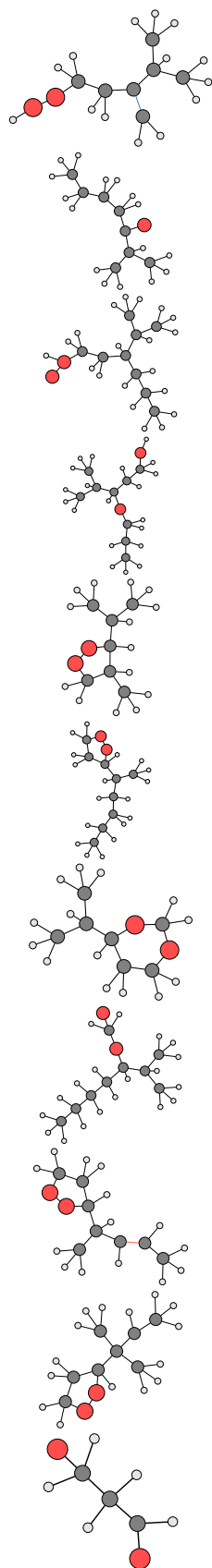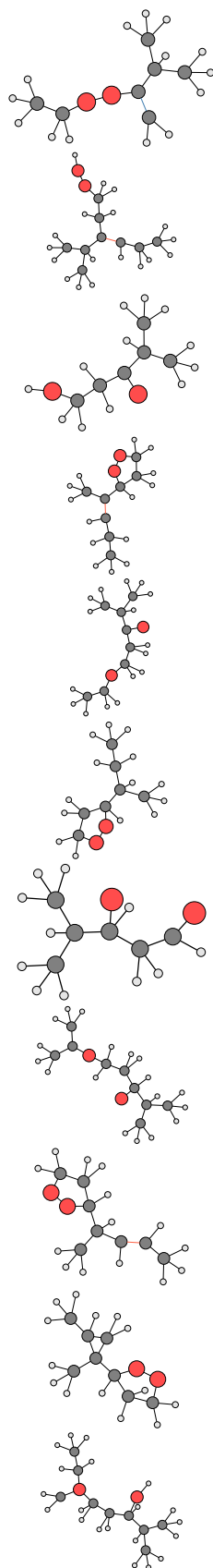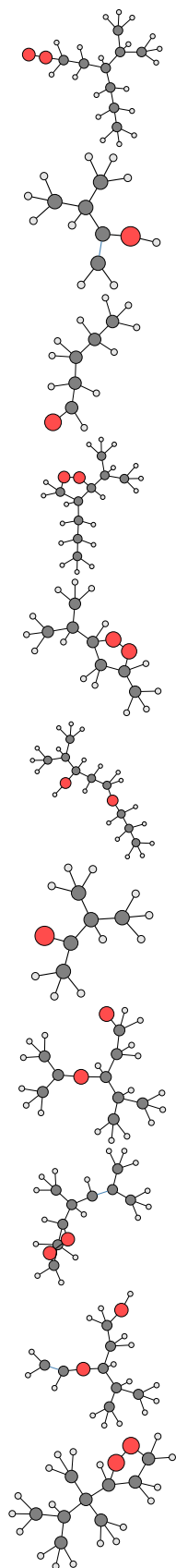

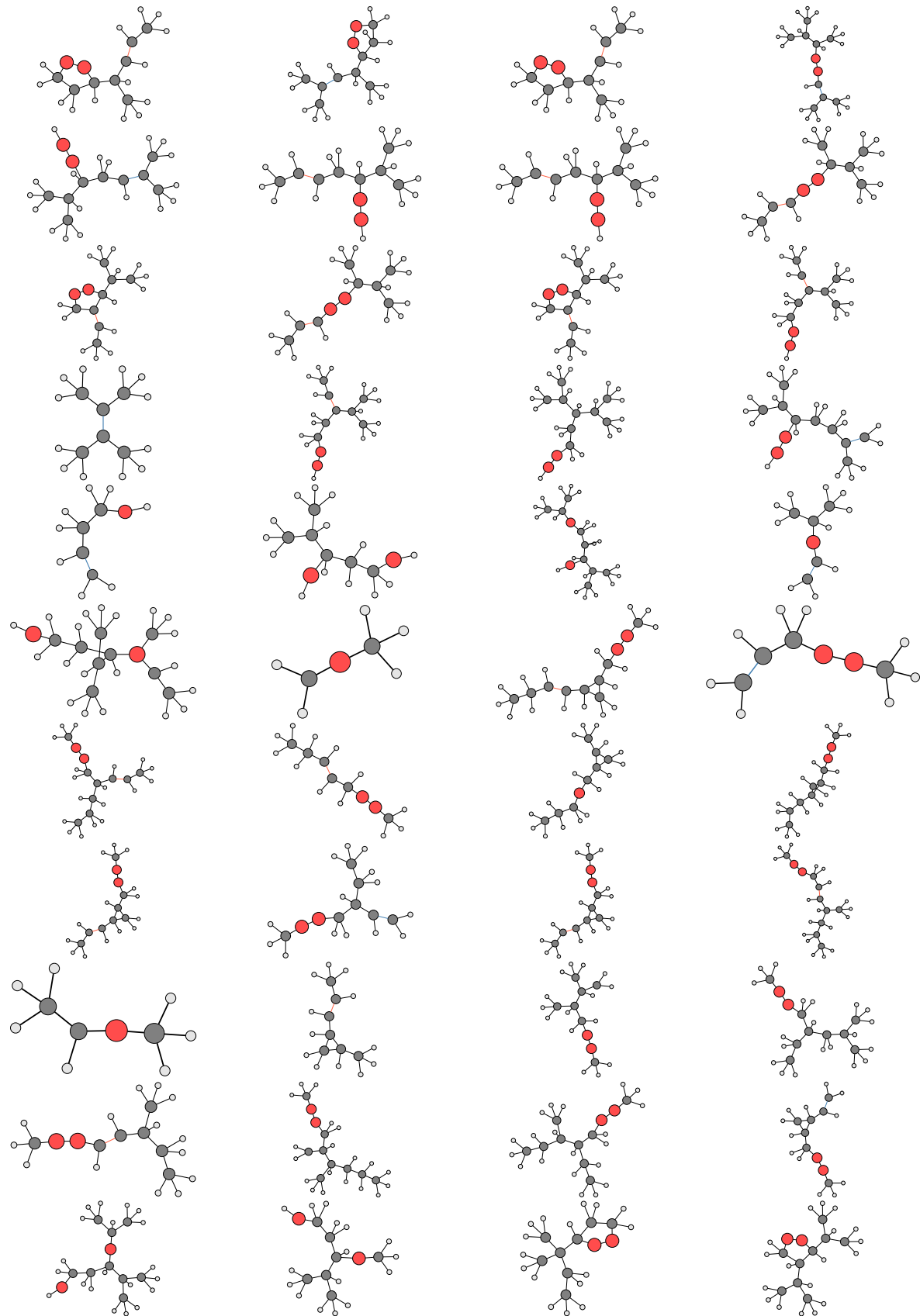

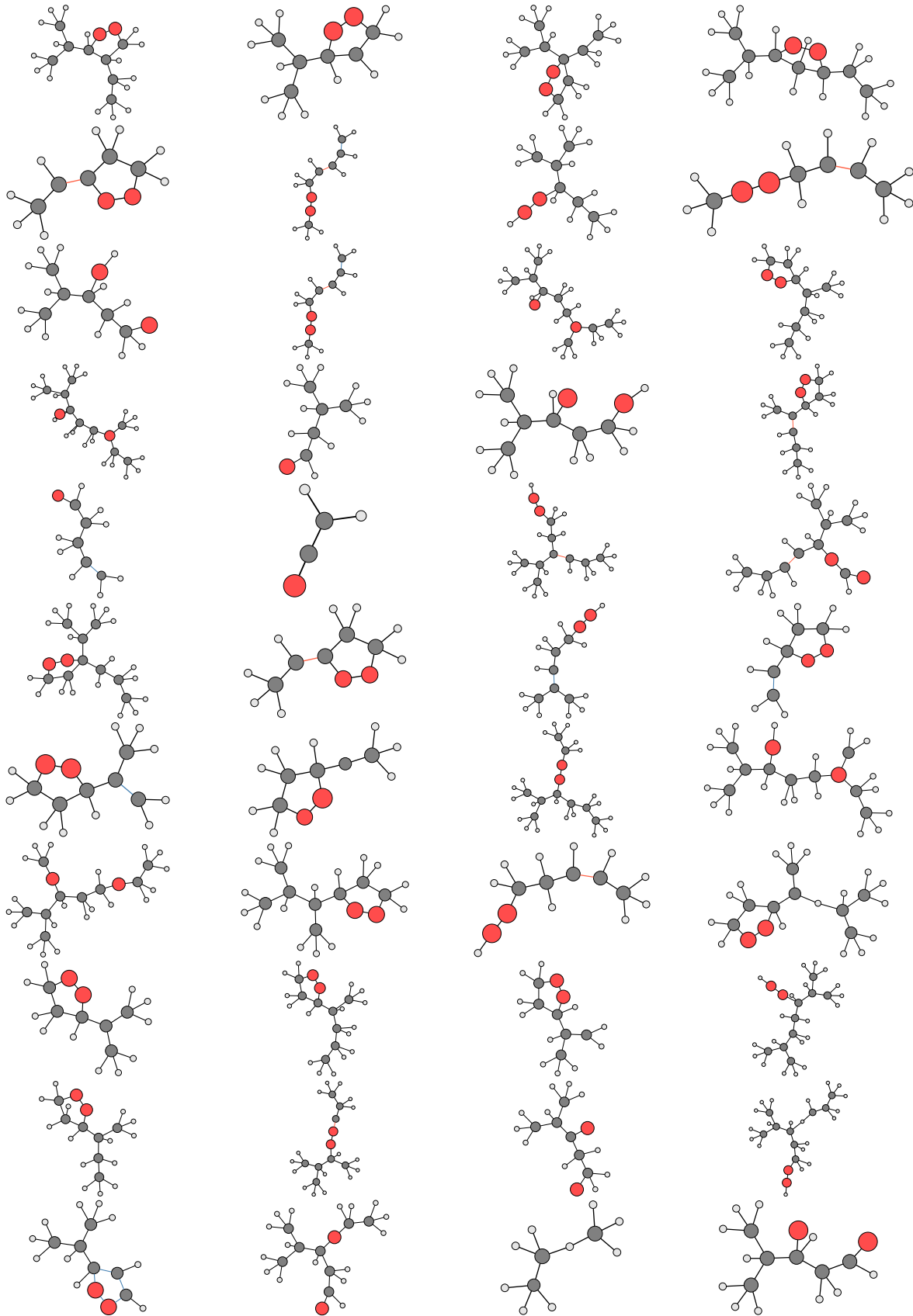

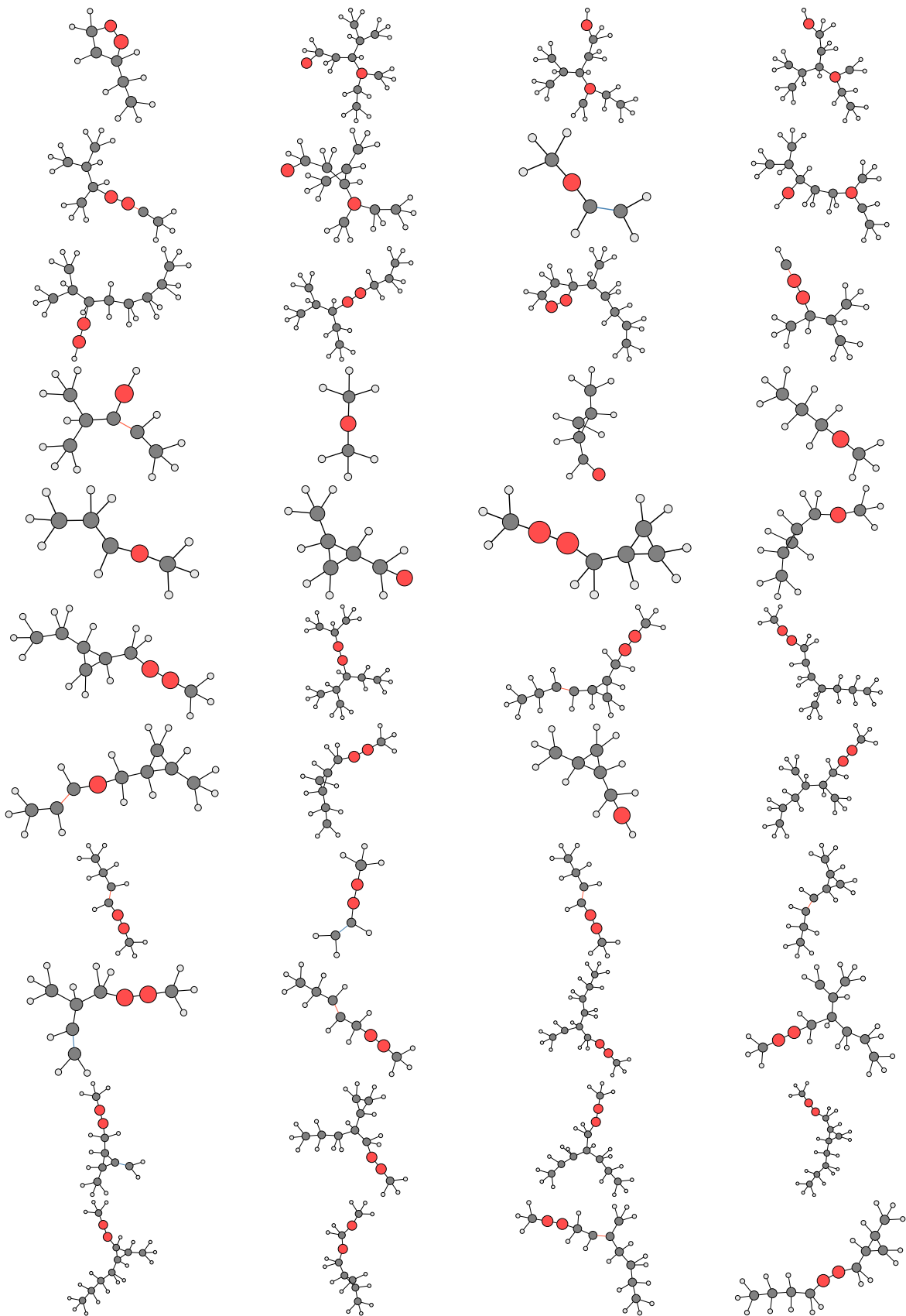

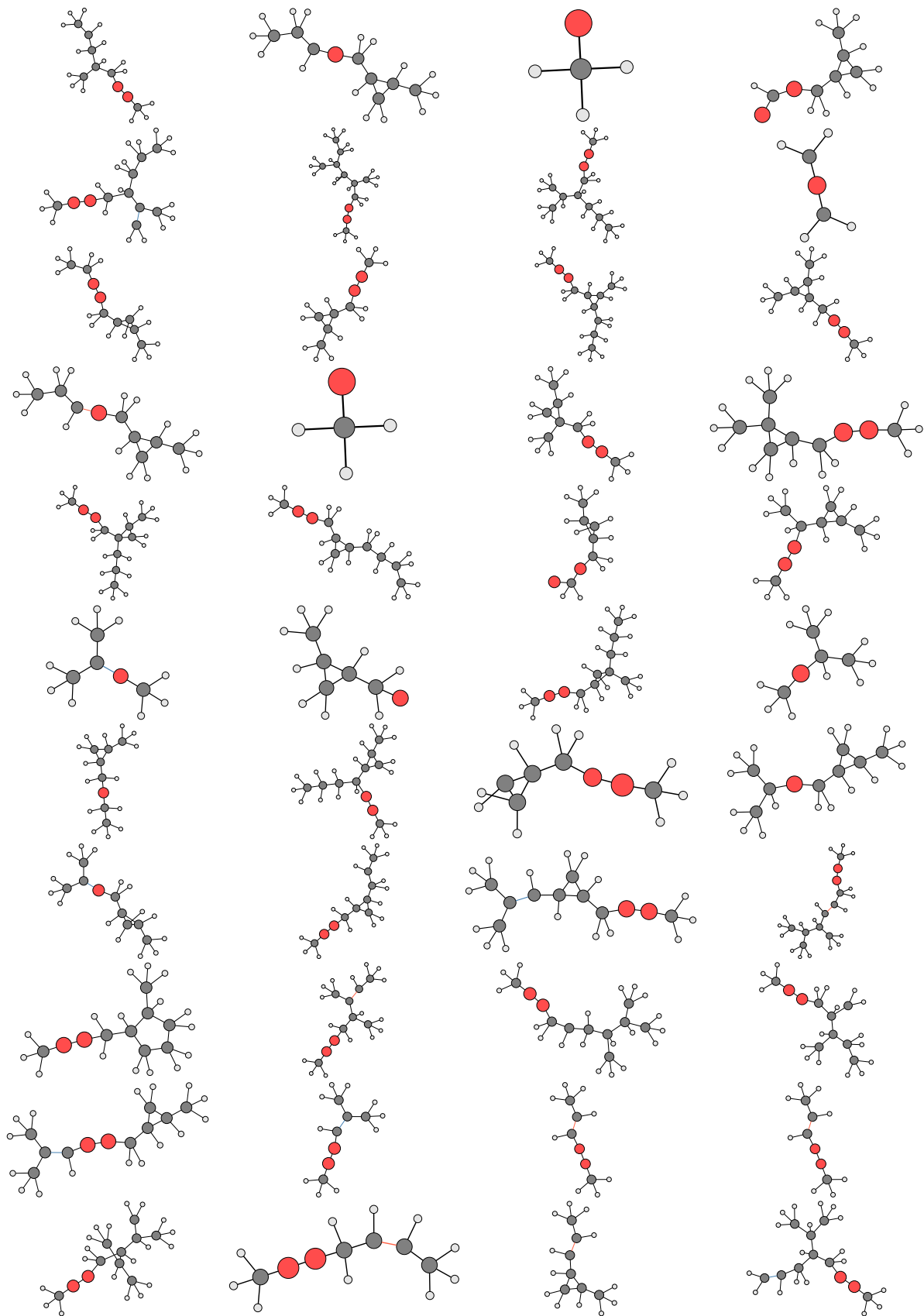

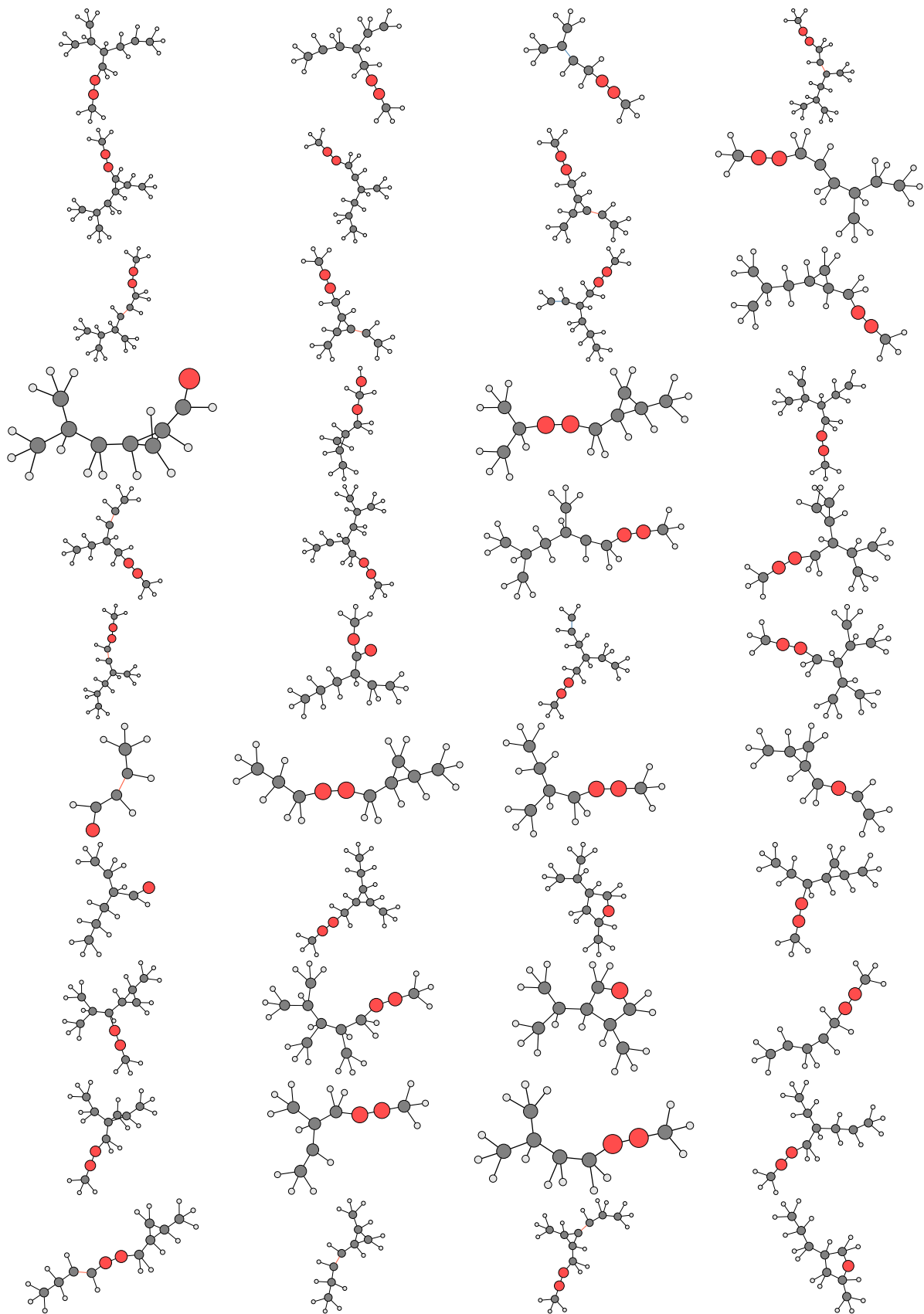

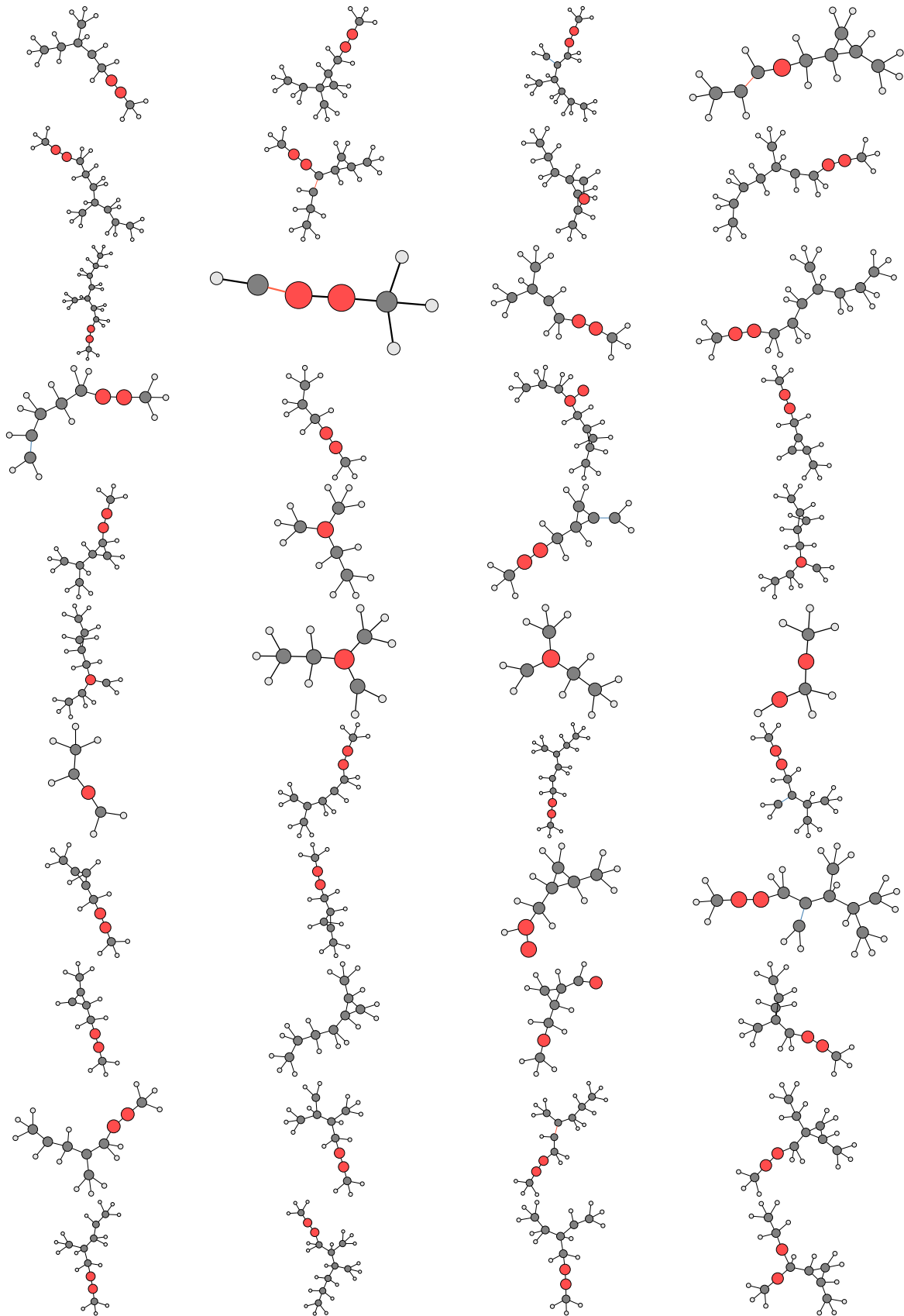

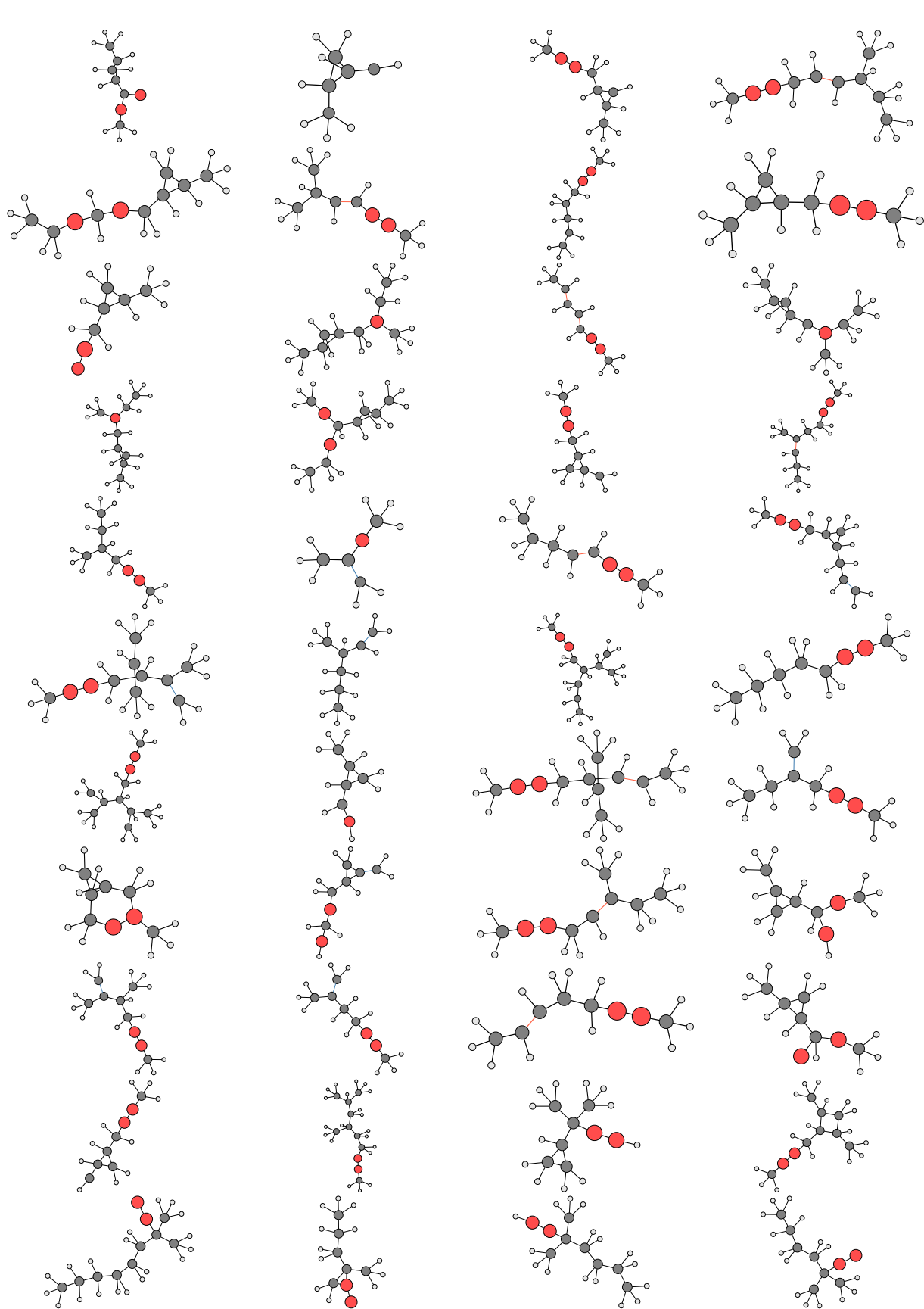

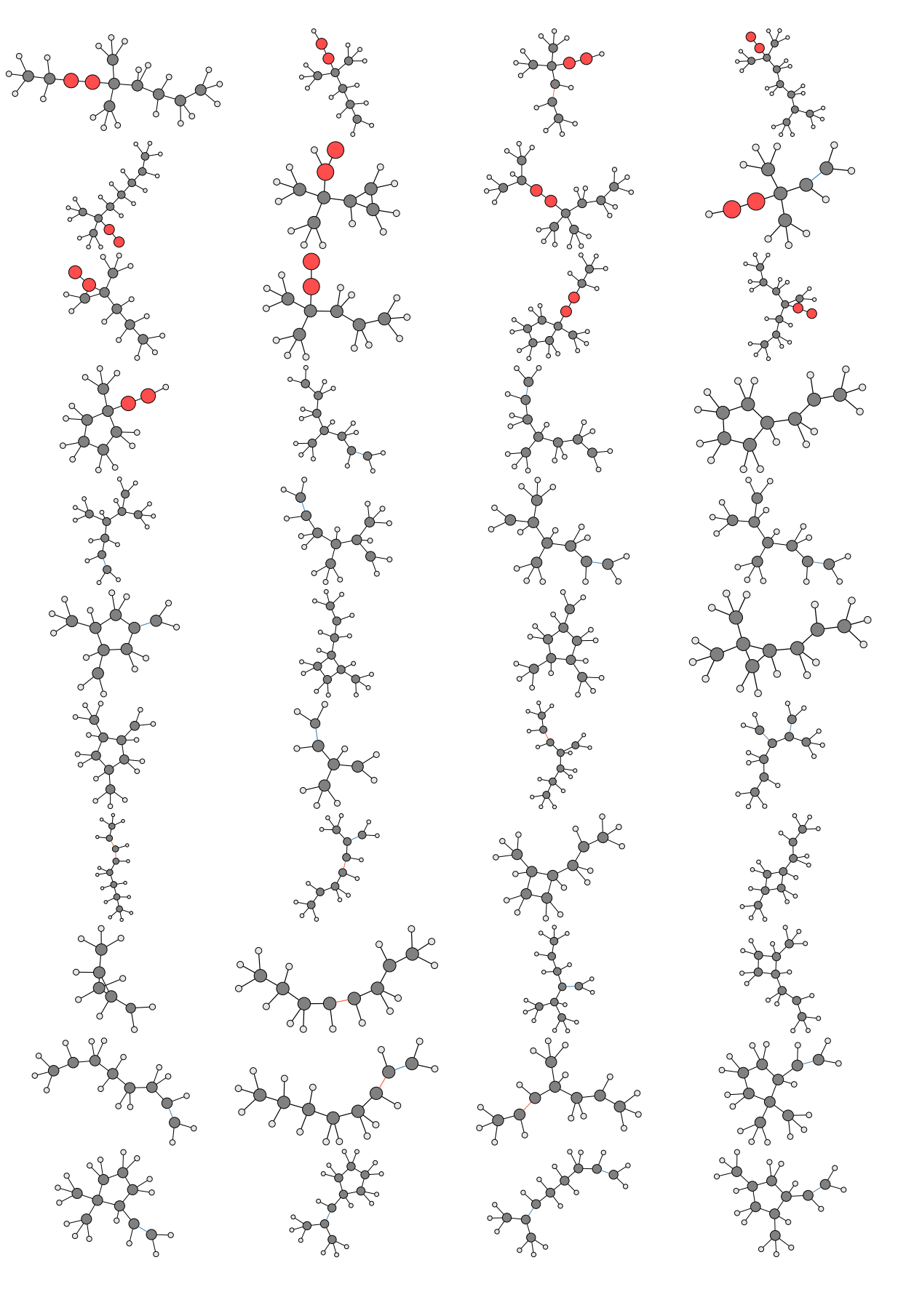

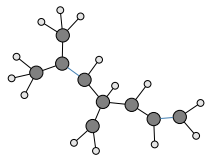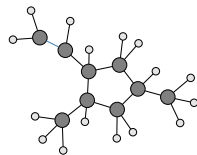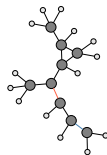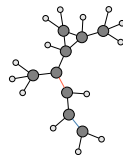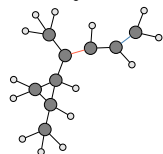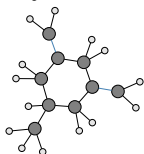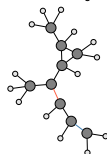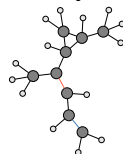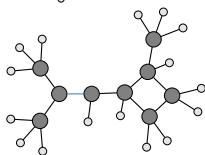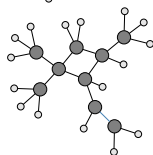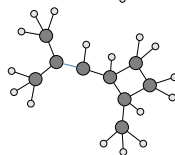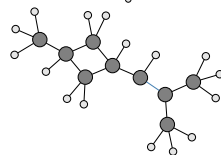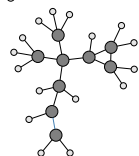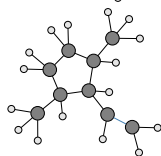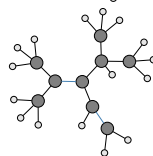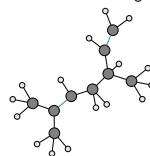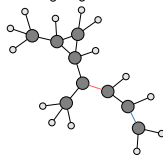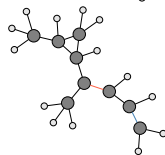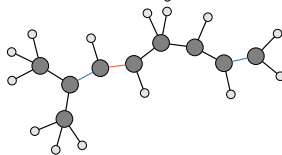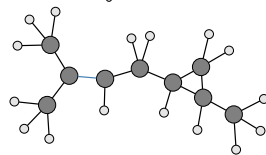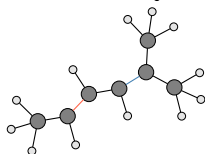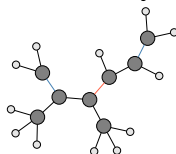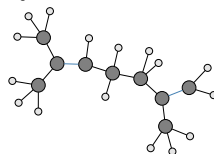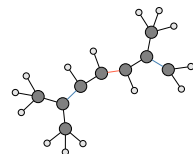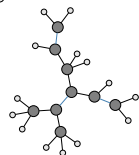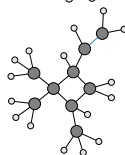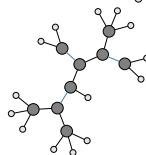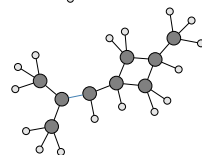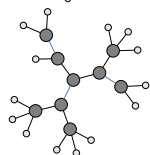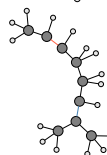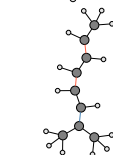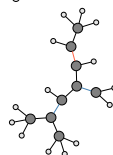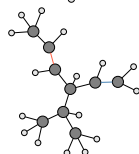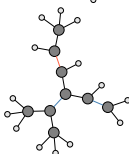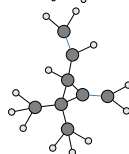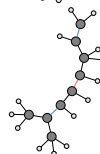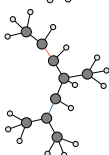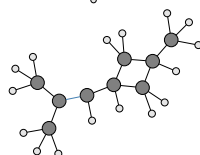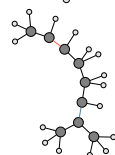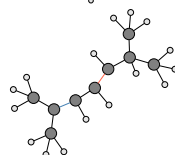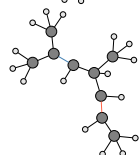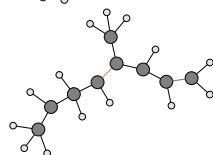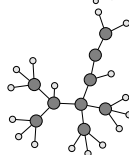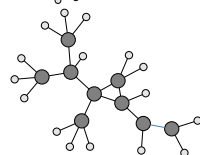

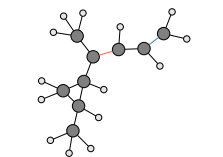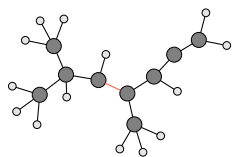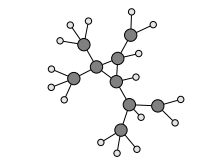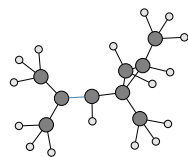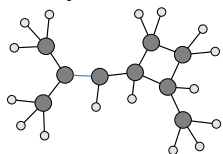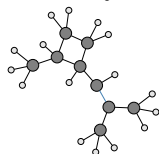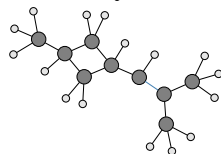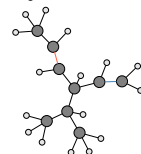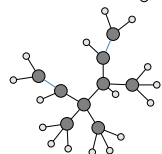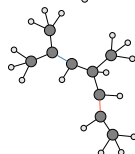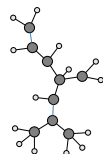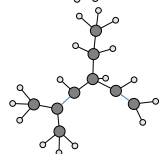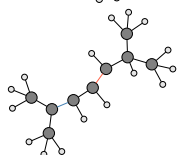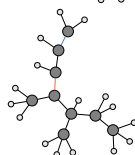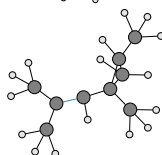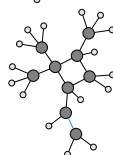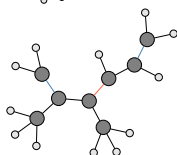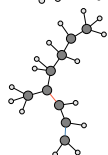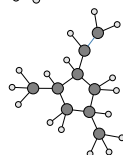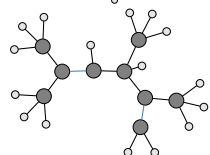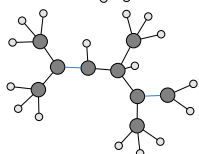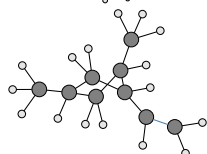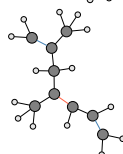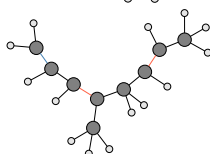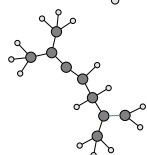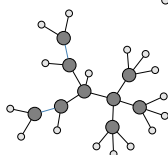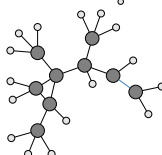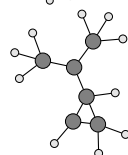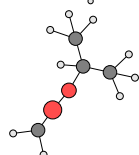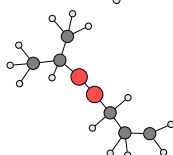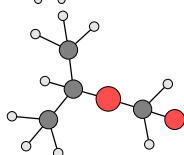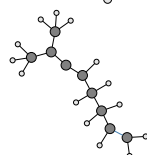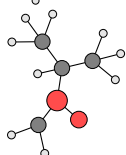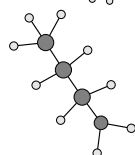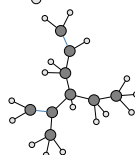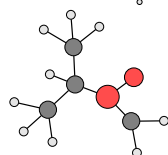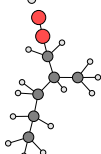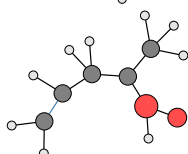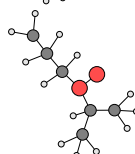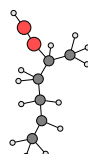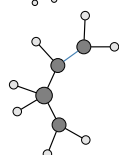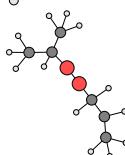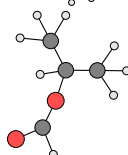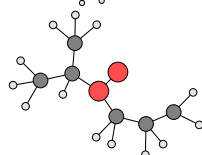

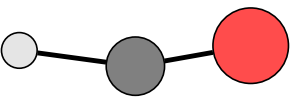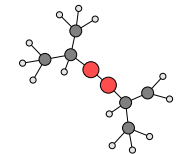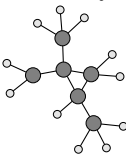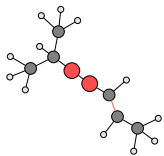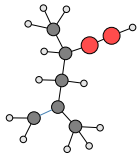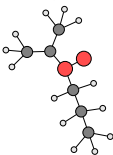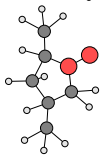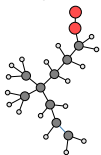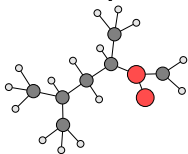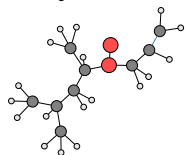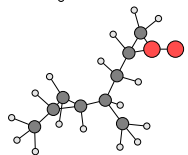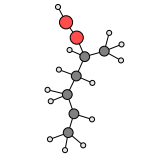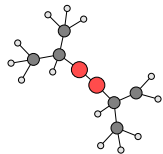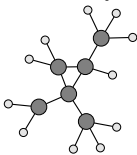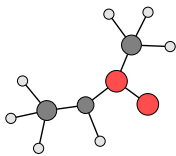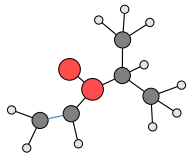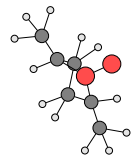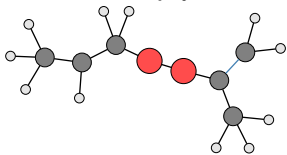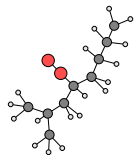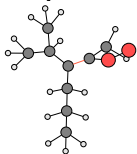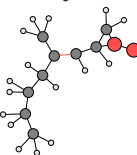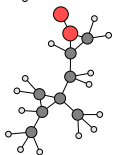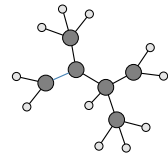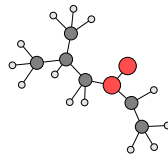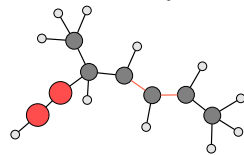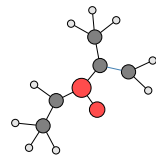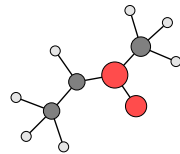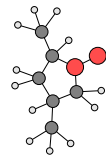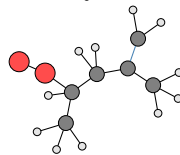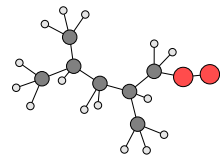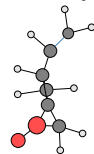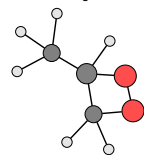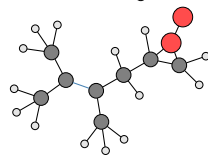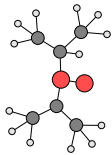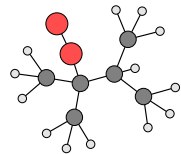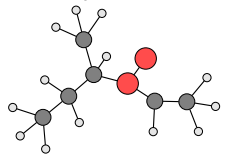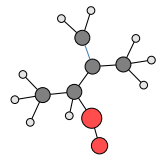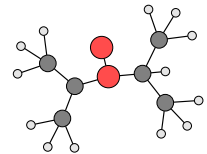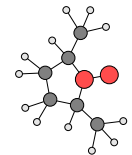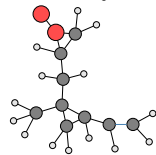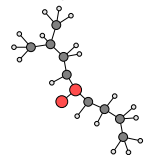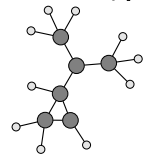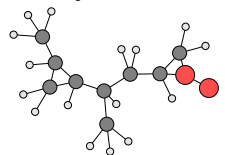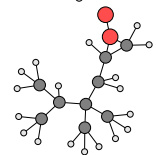

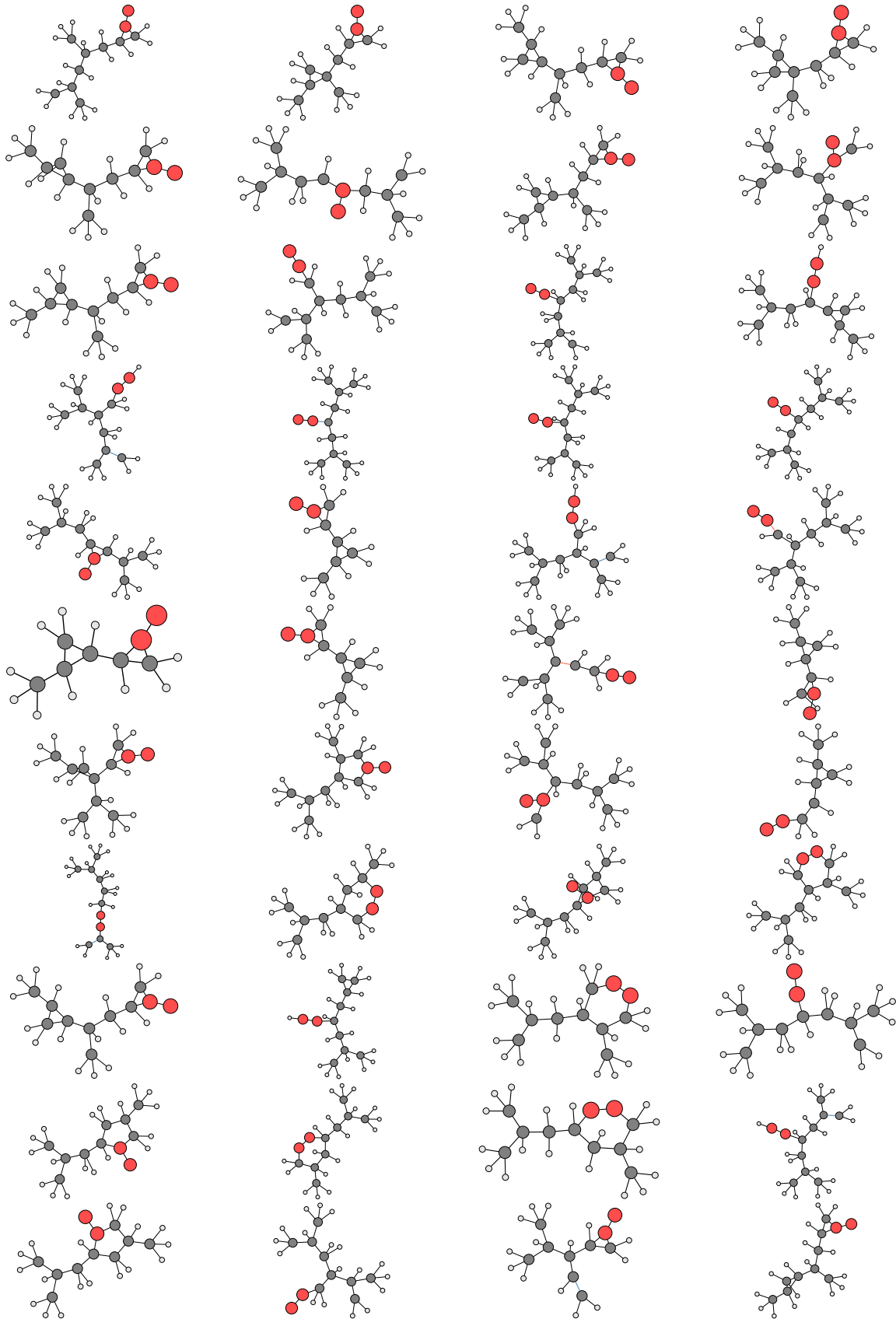

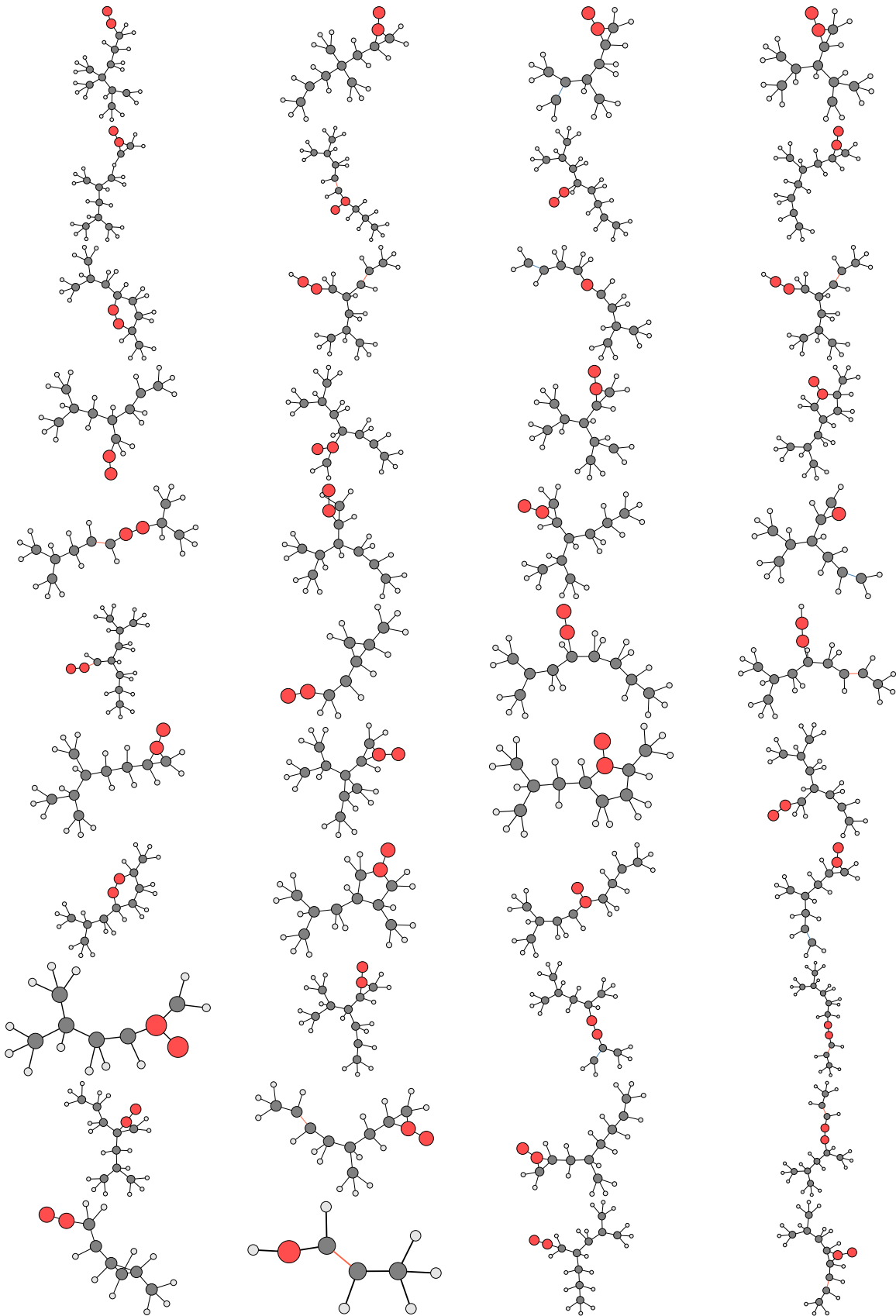

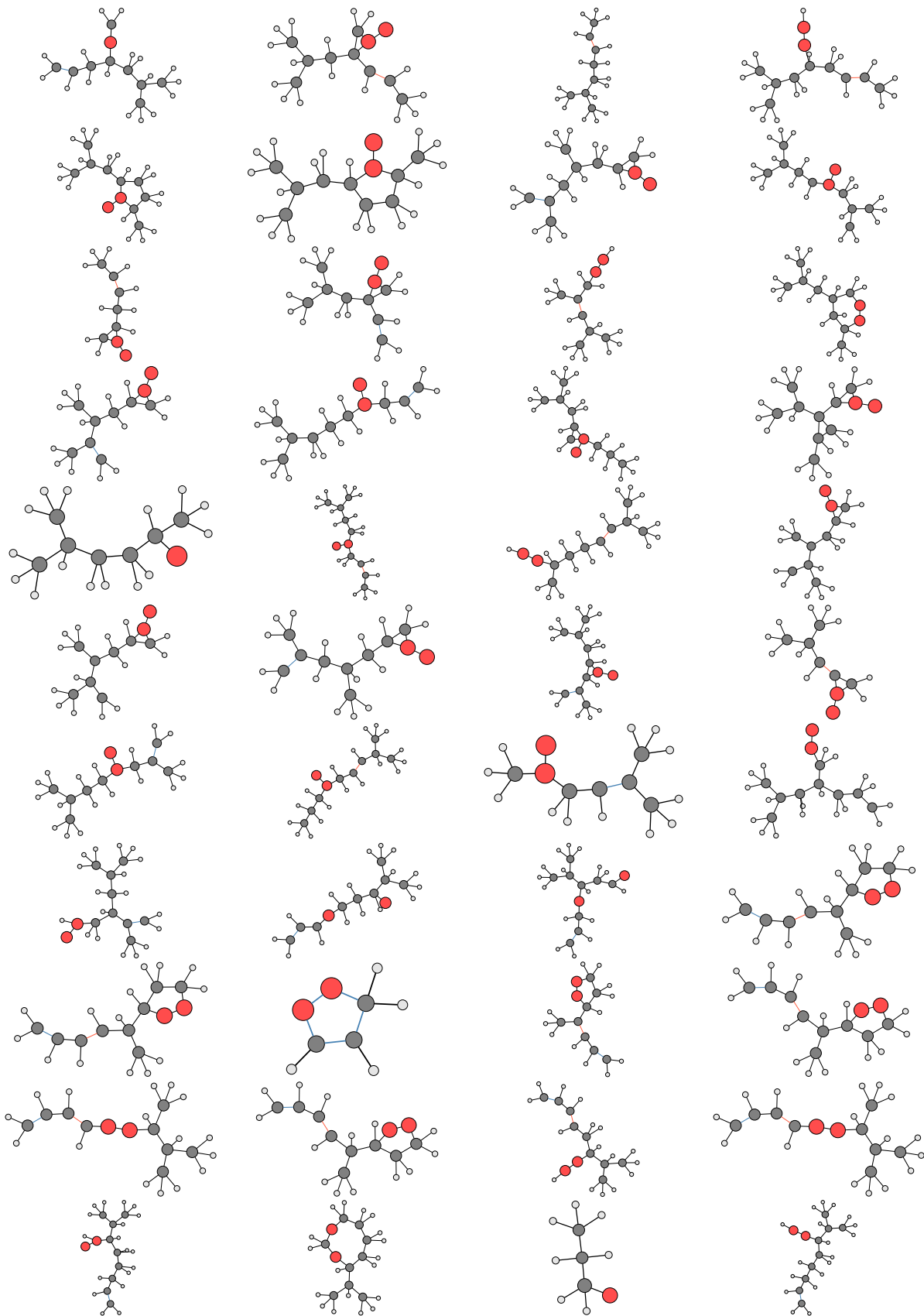

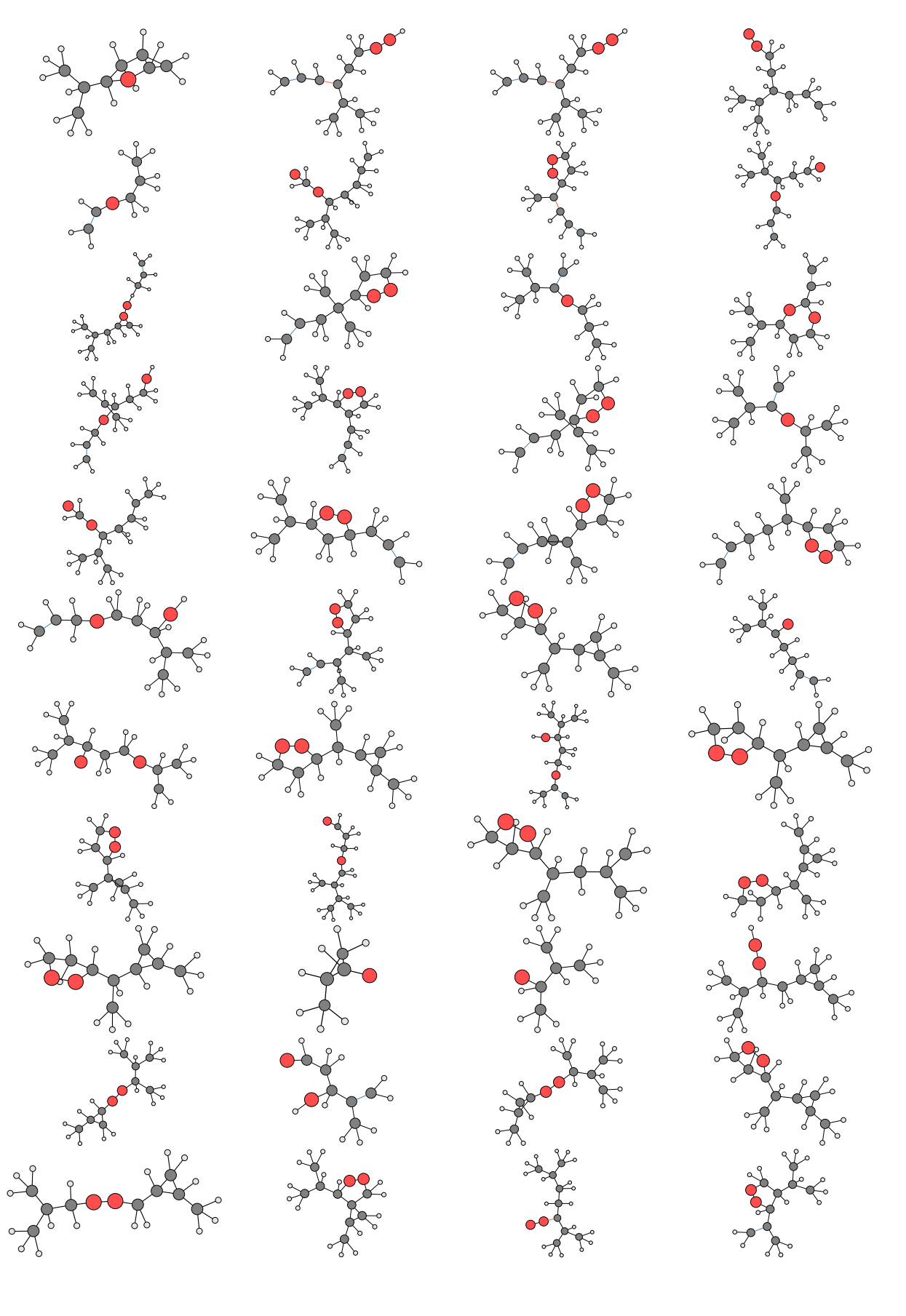

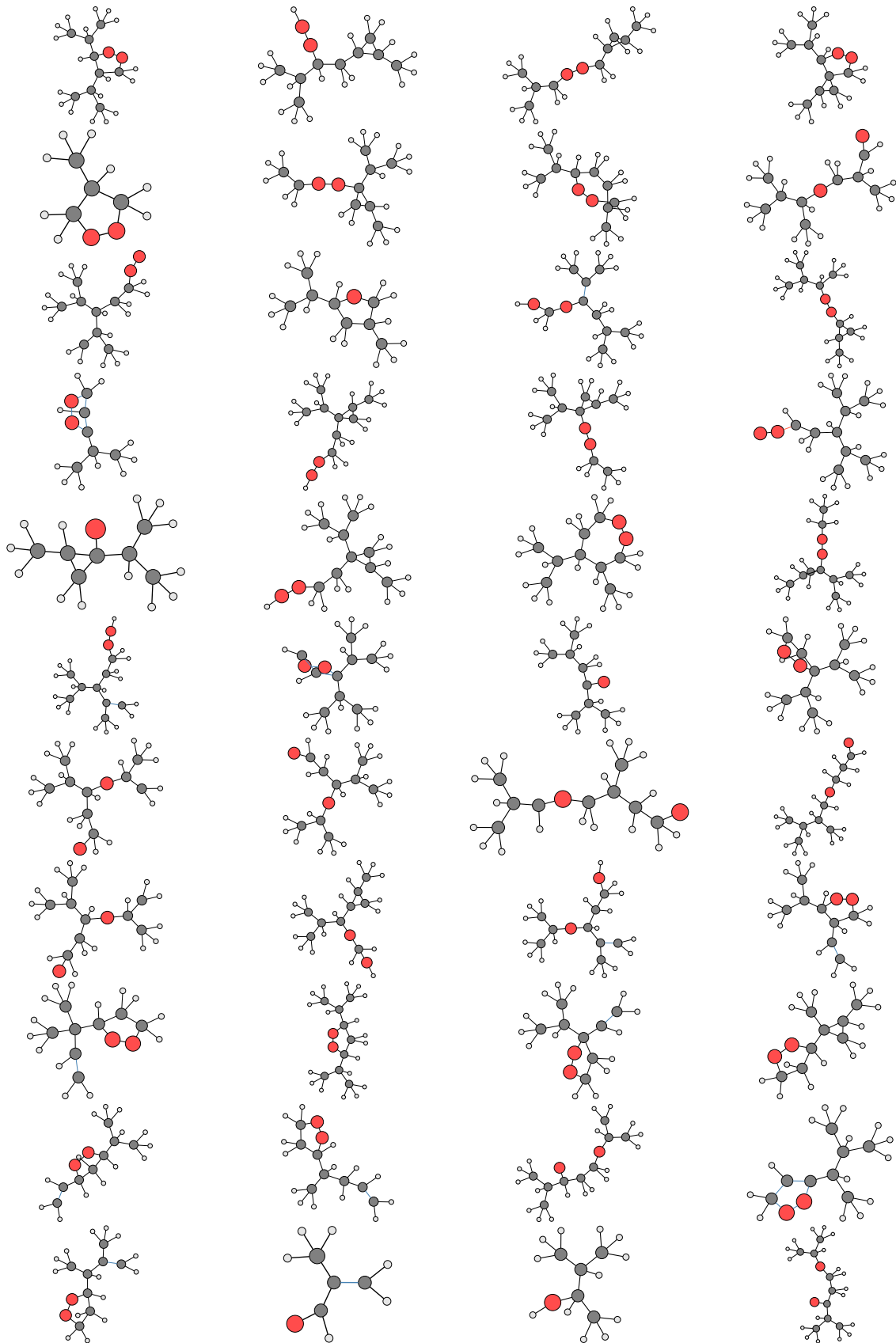

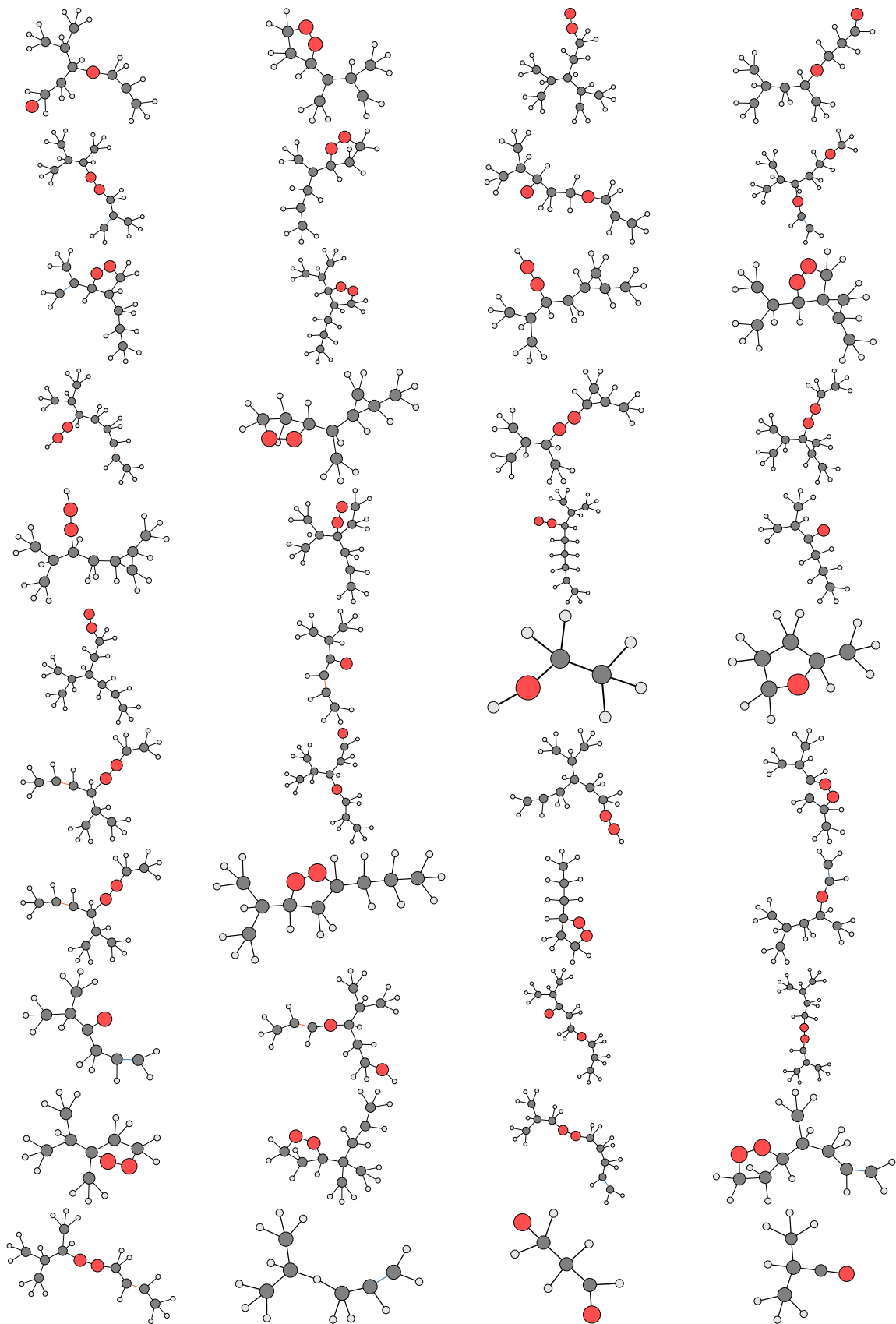

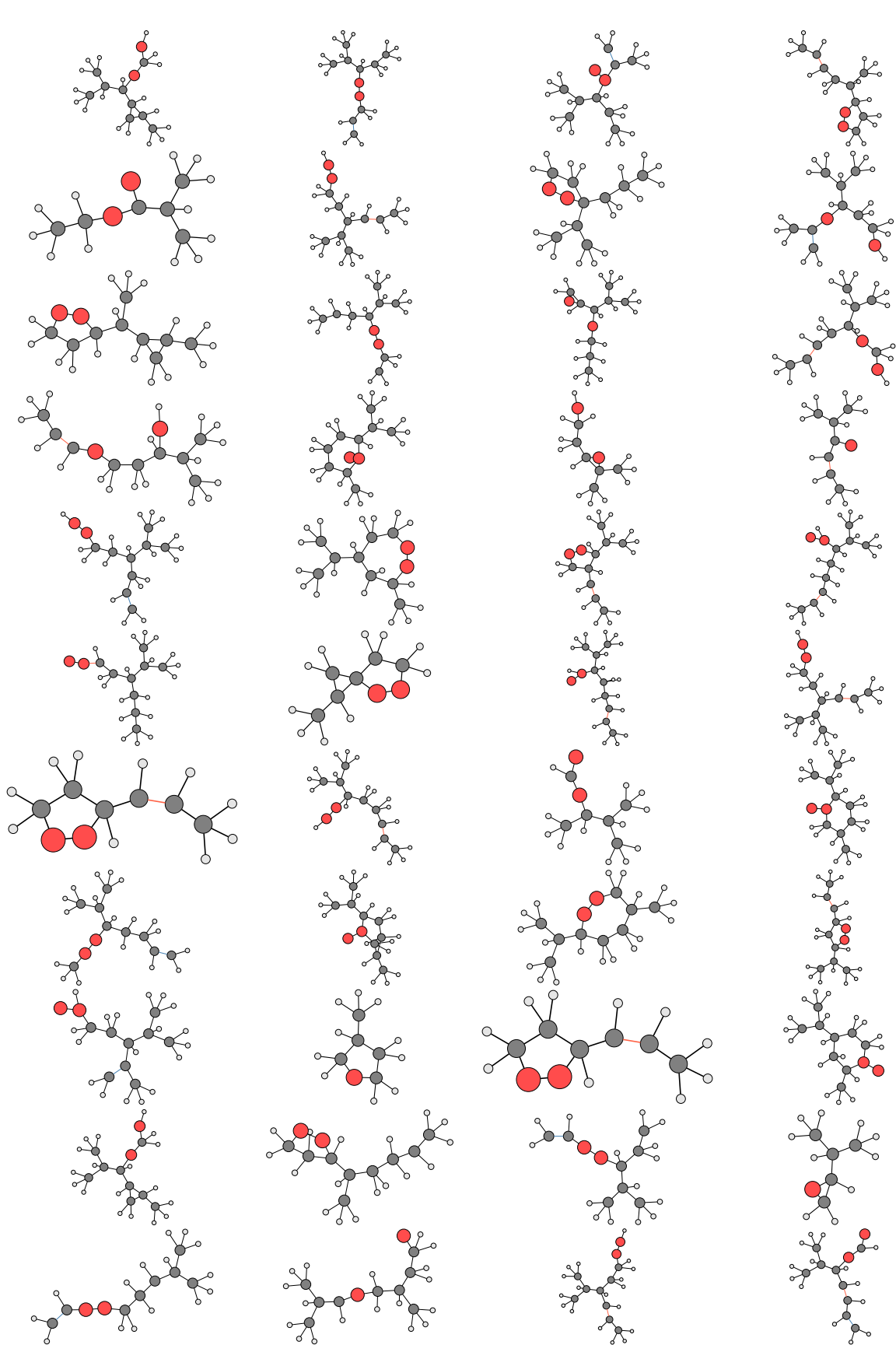

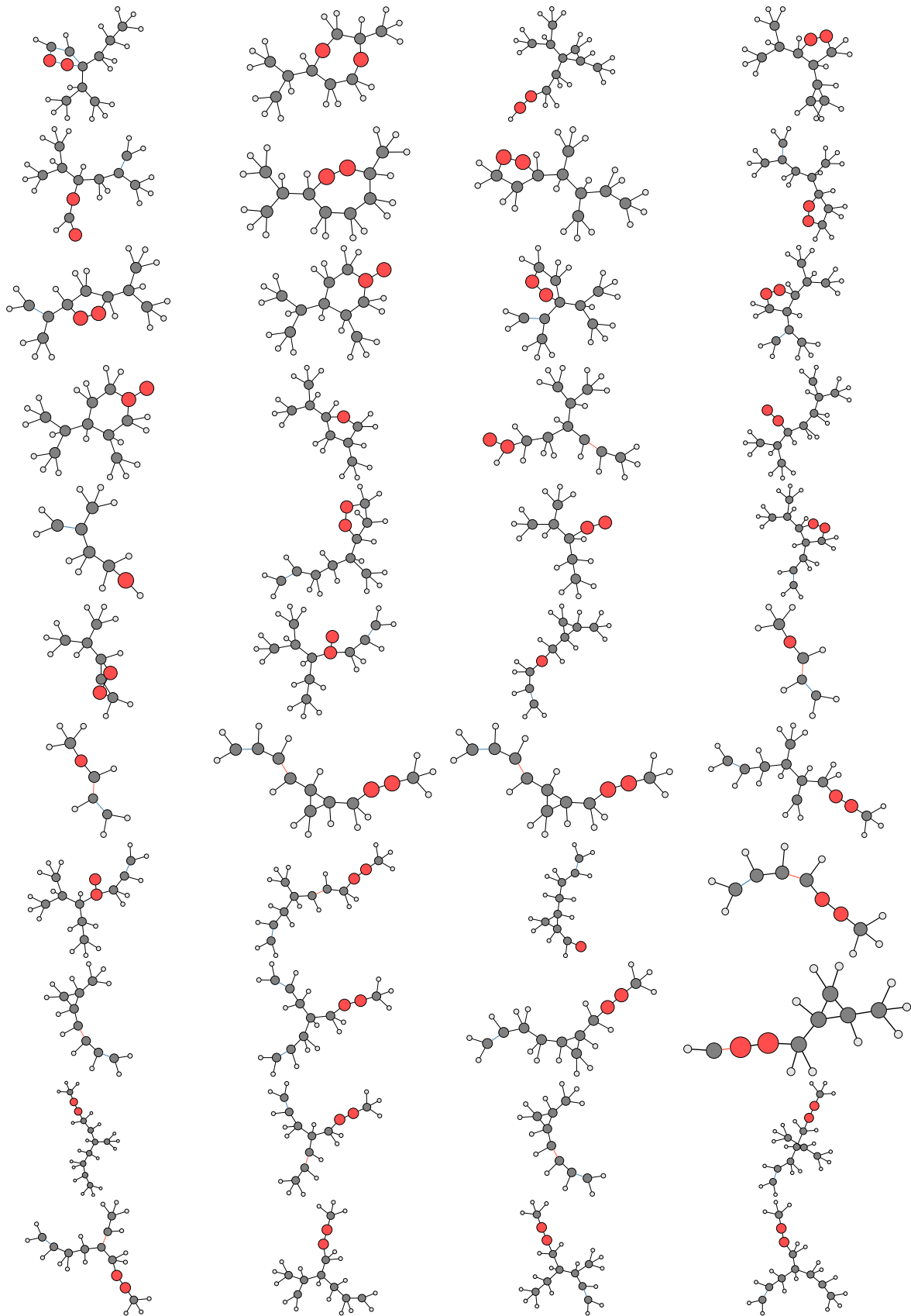



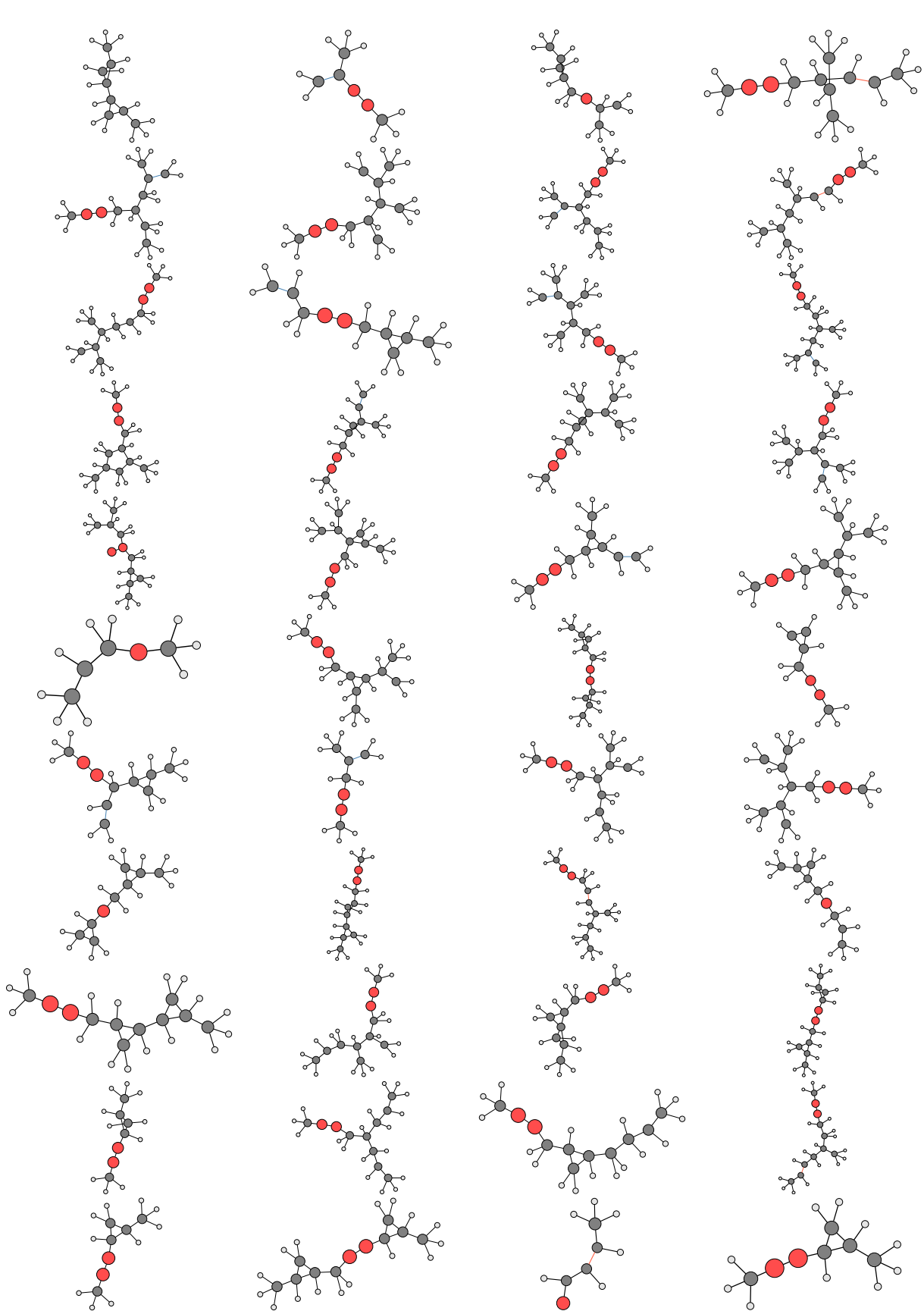

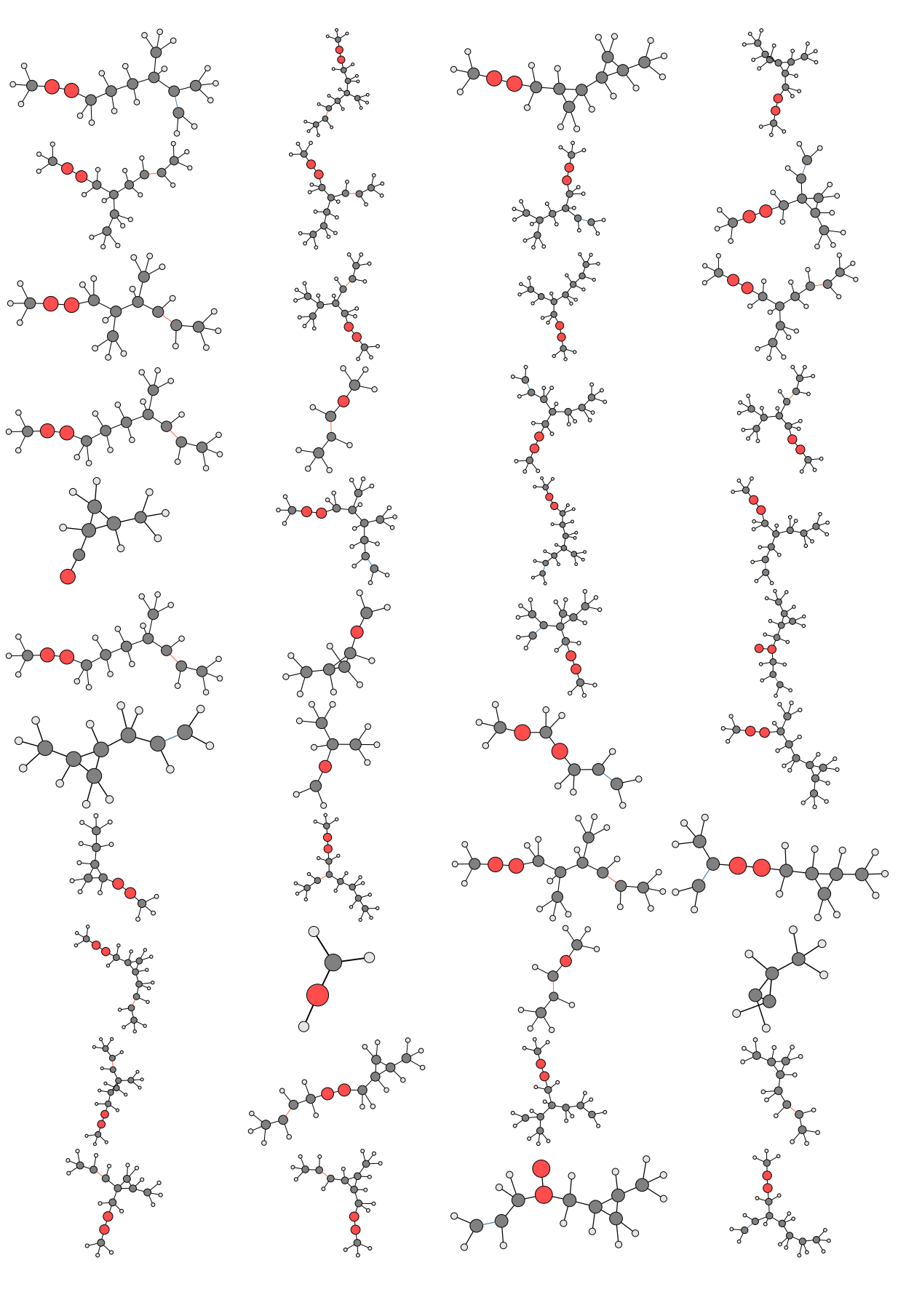

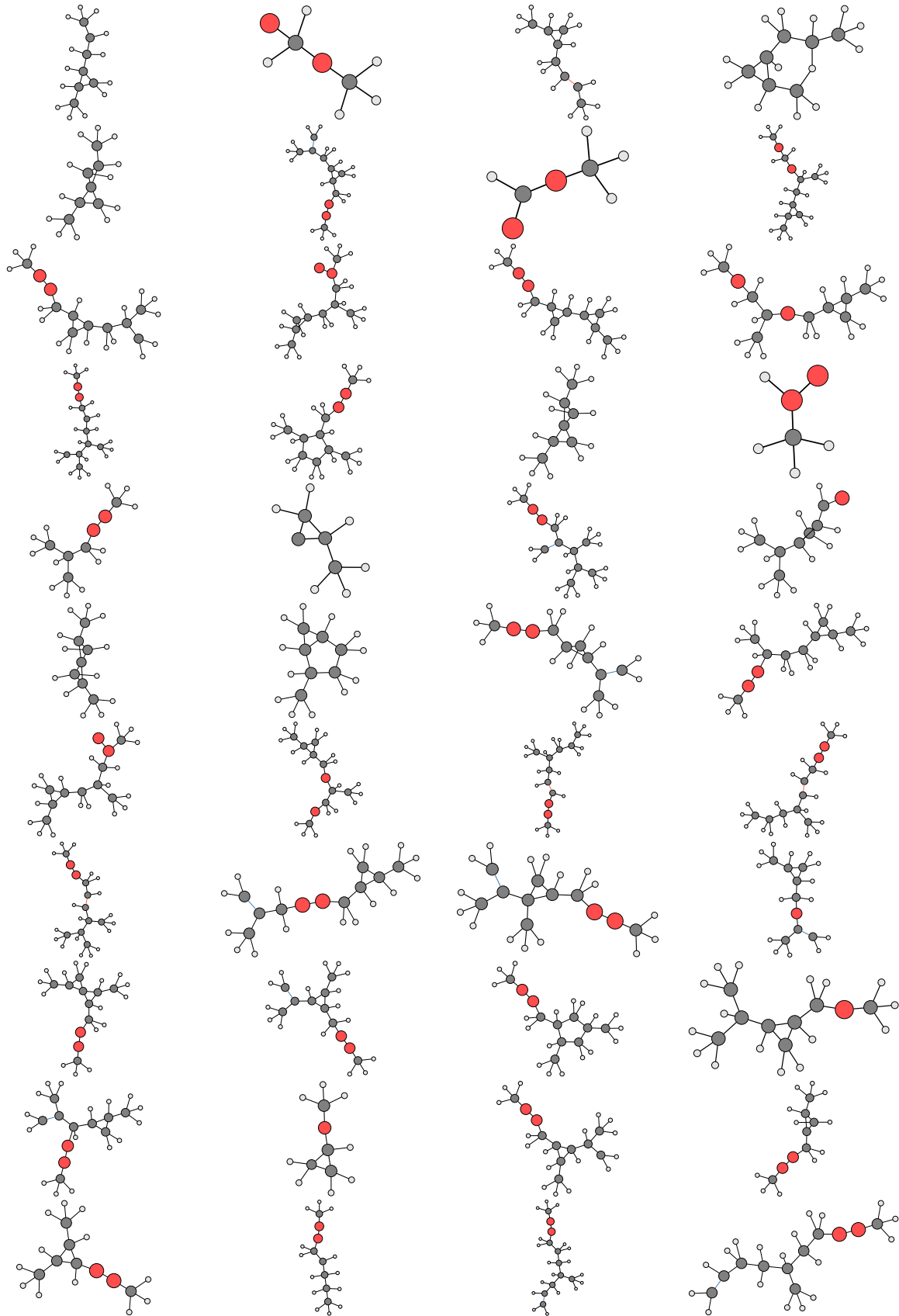

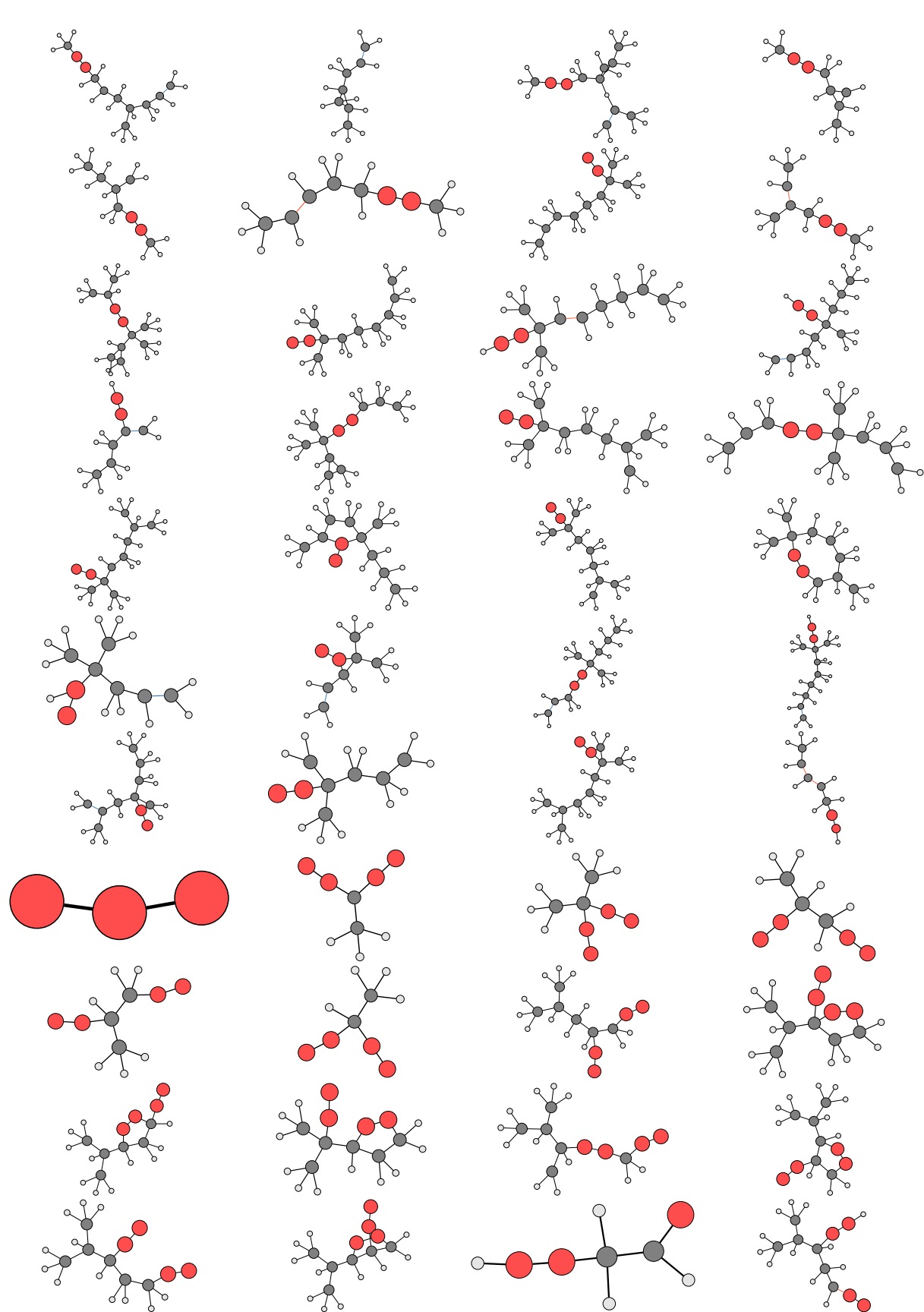

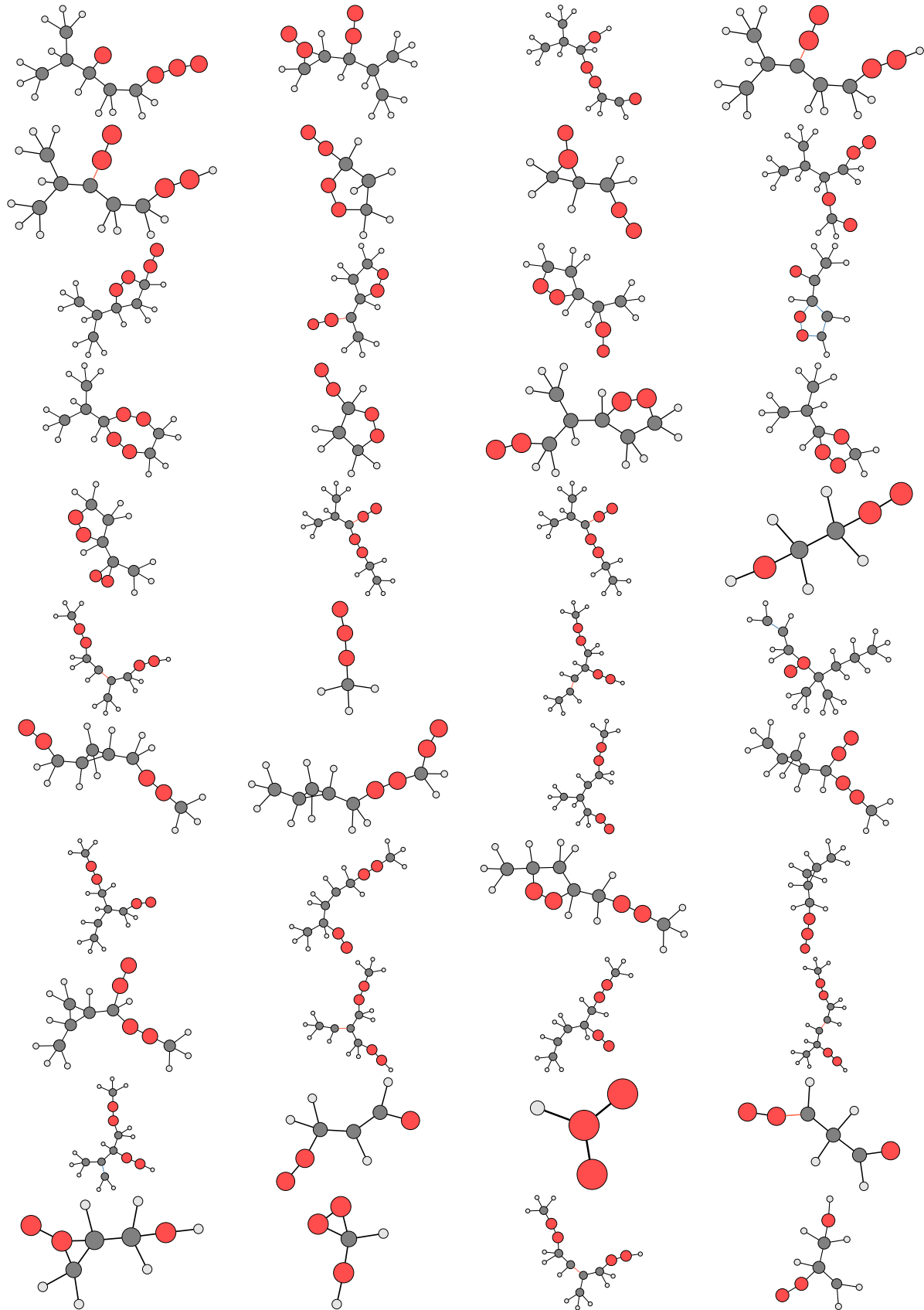

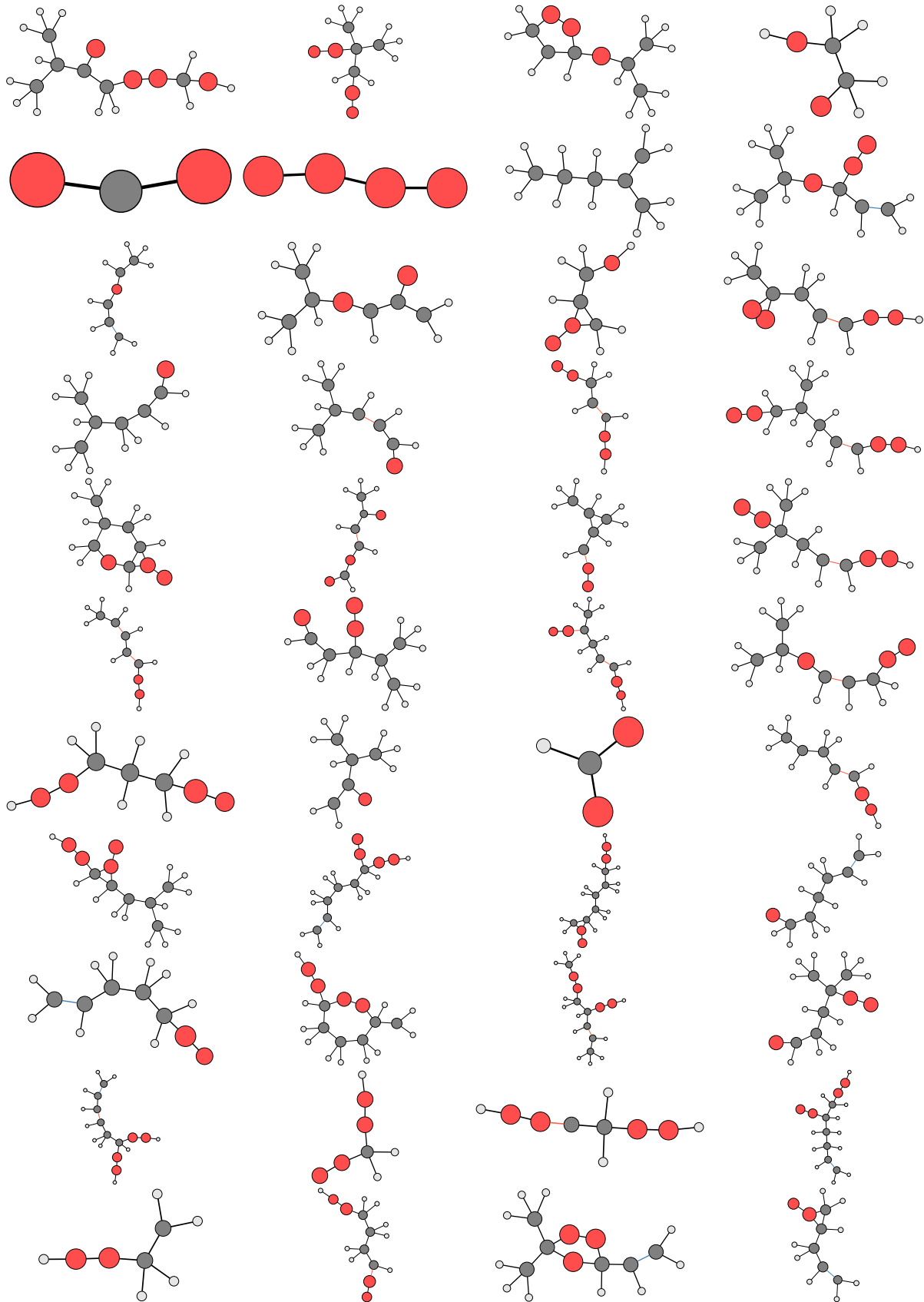

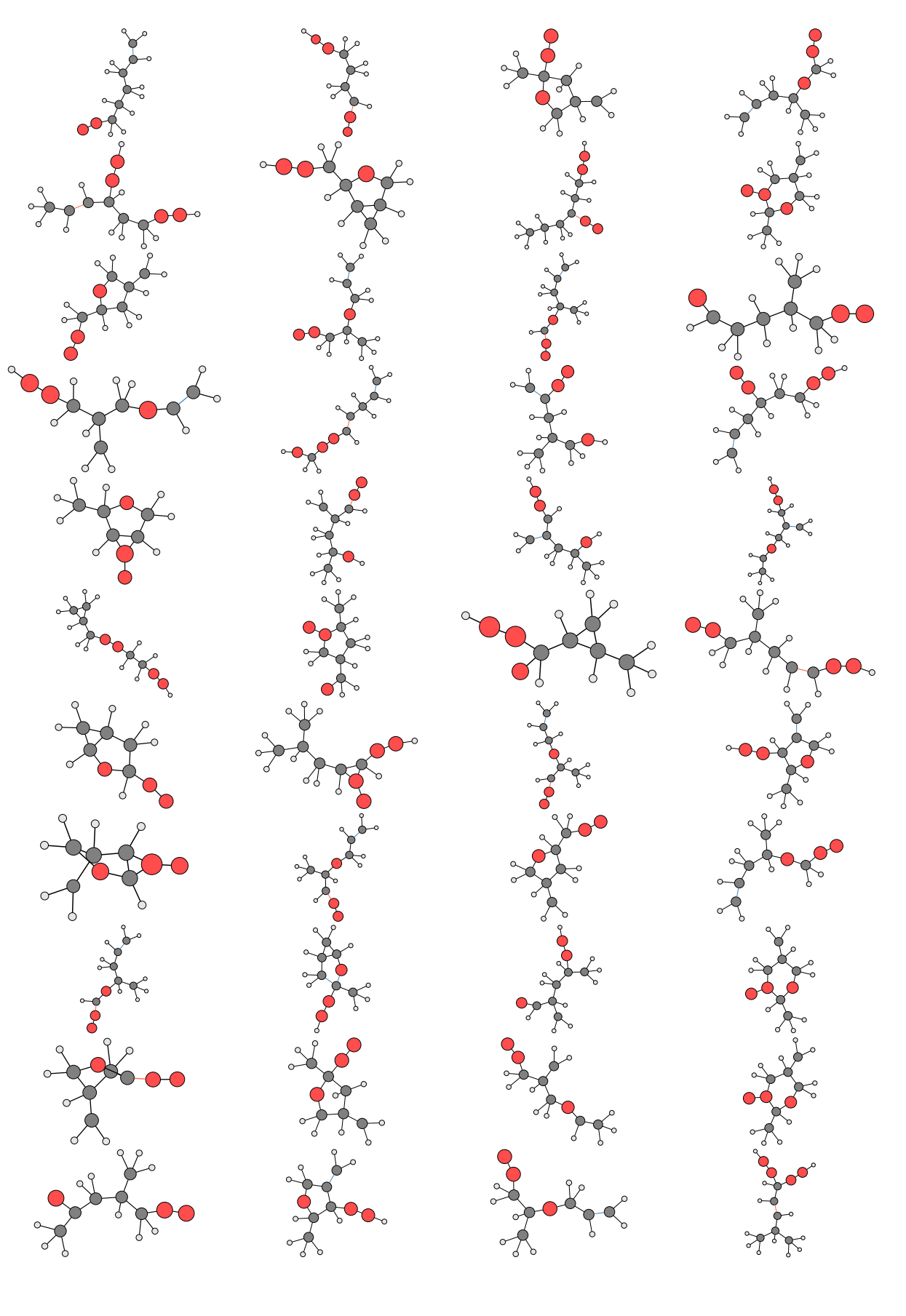

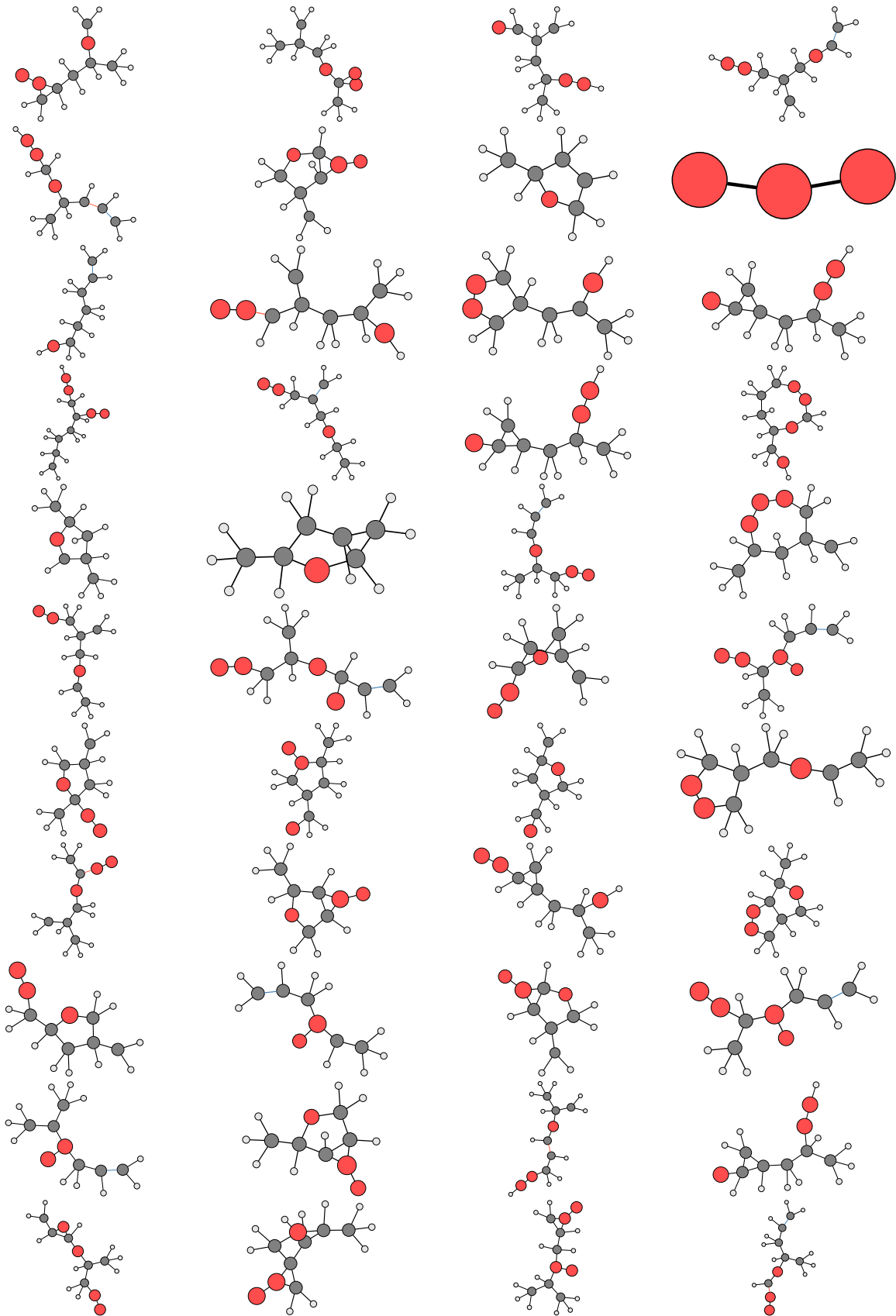

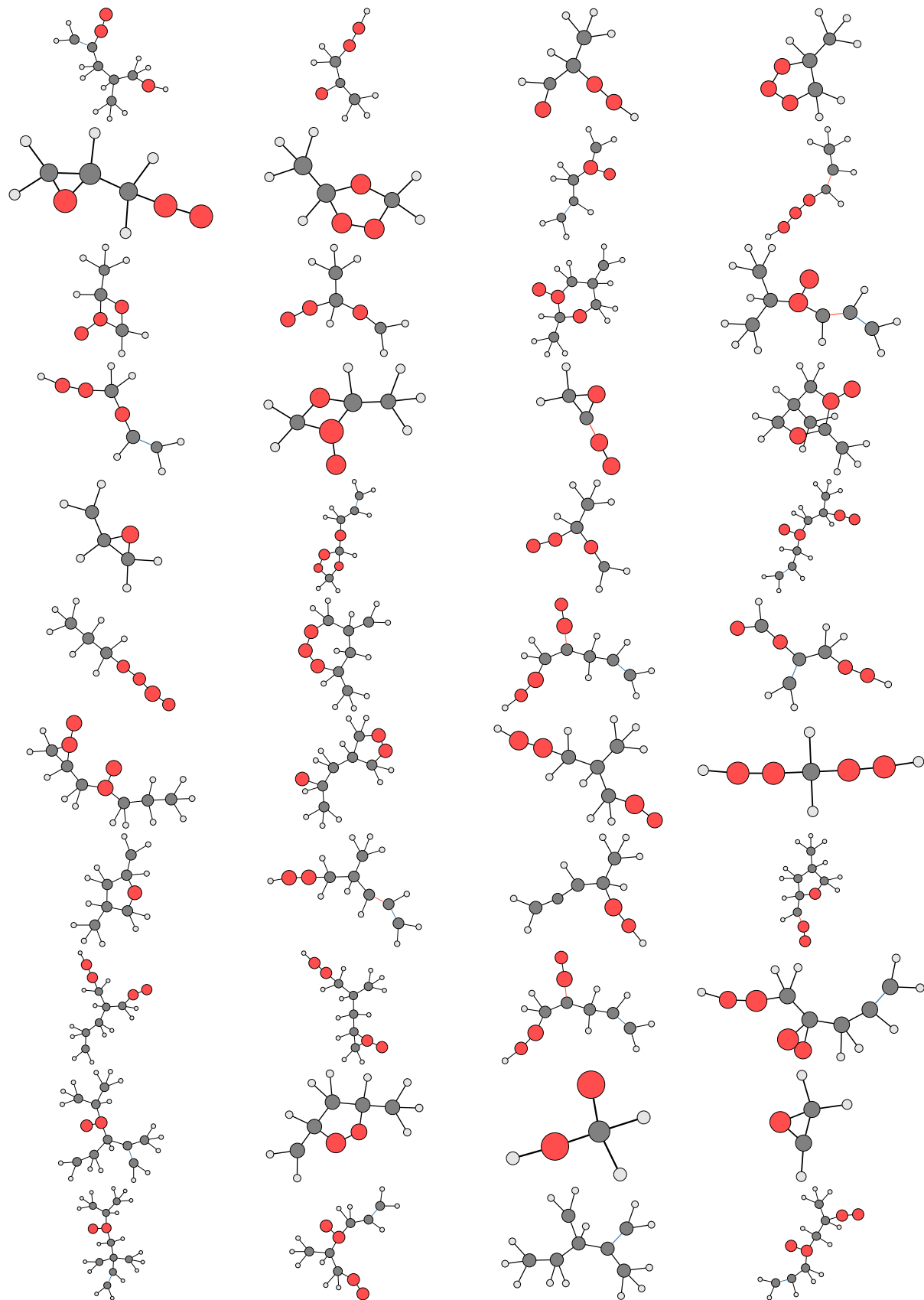

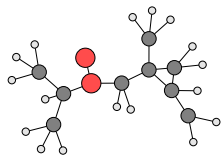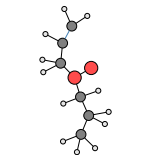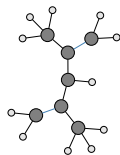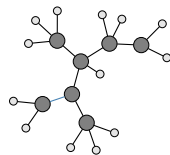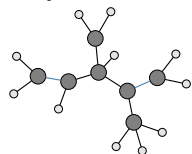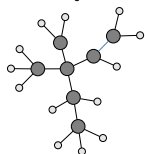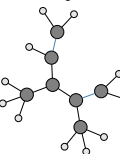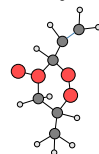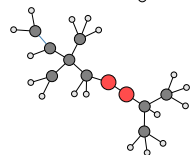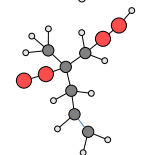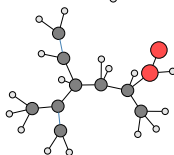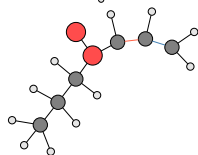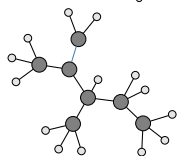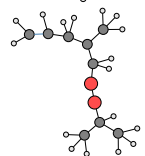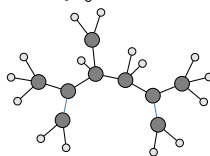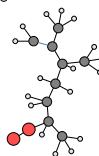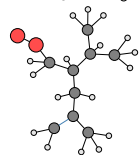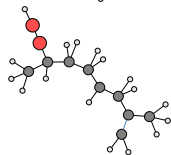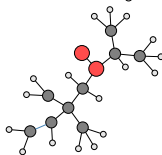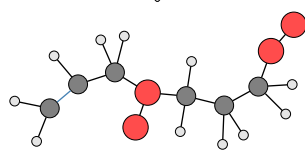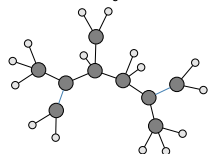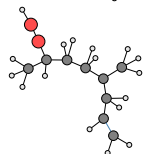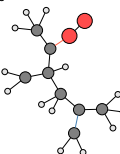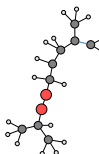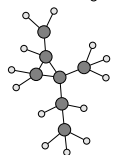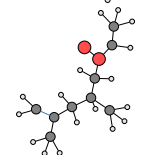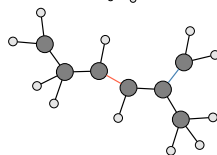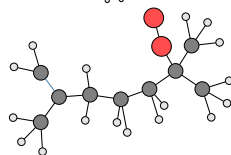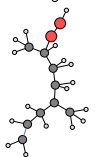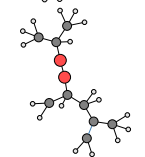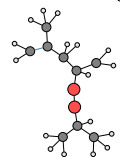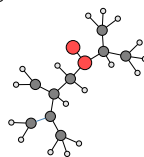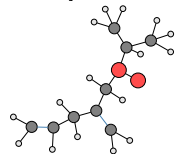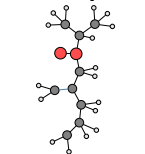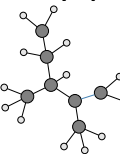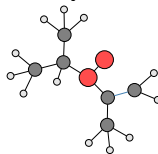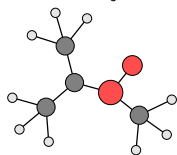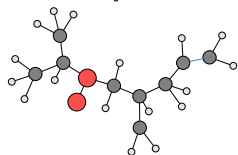

Supplement: Supplementary file 1 — Supplementary file1 (PDF 10017 kb) [file 11244_2021_1543_MOESM1_ESM.pdf]
